# Using Explainable Machine Learning to Interpret the Effects of Policies on Air Pollution: COVID-19 Lockdown in London

**DOI:** 10.1021/acs.est.2c09596

**Published:** 2023-08-11

**Authors:** Liang Ma, Daniel J. Graham, Marc E. J. Stettler

**Affiliations:** Department of Civil and Environmental Engineering, Imperial College London, London SW7 2AZ, United Kingdom

**Keywords:** Air pollution, Public health, COVID-19, Causal analysis, Explainable machine learning

## Abstract

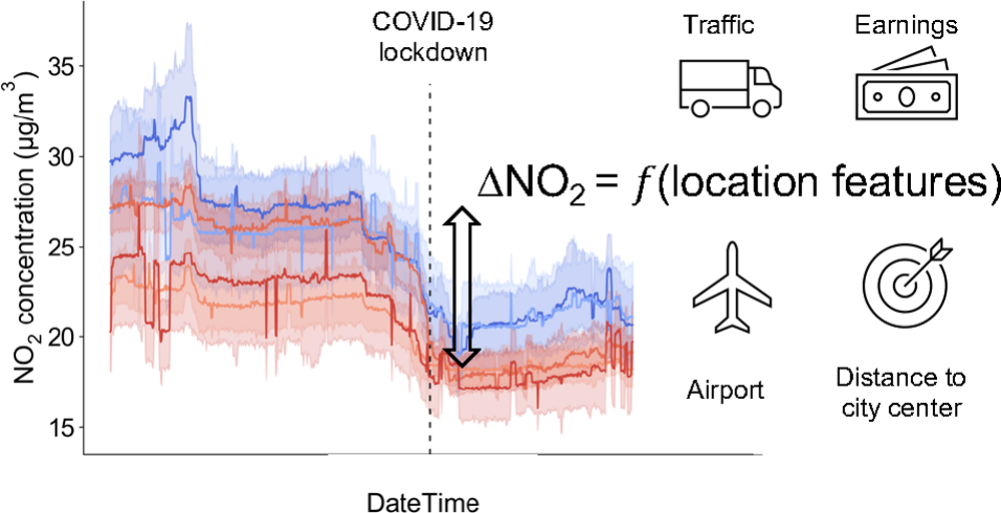

Activity changes during the COVID-19 lockdown present an opportunity to understand the effects that prospective emission control and air quality management policies might have on reducing air pollution. Using a regression discontinuity design for causal analysis, we show that the first UK national lockdown led to unprecedented decreases in road traffic, by up to 65%, yet incommensurate and heterogeneous responses in air pollution in London. At different locations, changes in air pollution attributable to the lockdown ranged from −50% to 0% for nitrogen dioxide (NO_2_), 0% to +4% for ozone (O_3_), and −5% to +0% for particulate matter with an aerodynamic diameter less than 10 μm (PM_10_), and there was no response for PM_2.5_. Using explainable machine learning to interpret the outputs of a predictive model, we show that the degree to which NO_2_ pollution was reduced in an area was correlated with spatial features (including road freight traffic and proximity to a major airport and the city center), and that existing inequalities in air pollution exposure were exacerbated: pollution reductions were greater in places with more affluent residents and better access to public transport services.

## Abstract 2

We use causal inference and explainable machine learning to identify, quantify, and interpret heterogeneous changes to air pollution in response to the first COVID-19 lockdown in London.

## Introduction

Air pollution exposure is the second leading cause of noncommunicable diseases worldwide.^1^ Particularly, over half of the world’s population living in urban areas was exposed to ambient air pollution levels at least 2.5 times higher than the World Health Organization safety standard.^2^ As a result, cities have implemented various interventions to mitigate air pollution. However, as a city is a complex network, the impact pathways from intervention to air quality can incorporate various factors (such as land use and demographics) and complicated interactions,^3^ which present challenges to isolating the intervention effect and quantifying the contribution of factors to the net effect. The recent development of interpretation methods for machine learning (ML) models provides an opportunity to gain insights into complicated relationships among high-dimensional variables. Unlike “black-box” ML, explainable ML seeks to make the processes and outputs of the model more interpretable and understandable to humans.^4^ These methods have been used to support decision-making in various fields, including healthcare,^5^ transport safety,^6^ and epidemiology.^7^

Coronavirus disease 19 (COVID-19) has rapidly spread worldwide since it was initially identified in late 2019. In response, several countries implemented lockdown measures to limit nonessential activities and restrict close contacts to varying degrees. The lockdown requirements, ongoing pandemic, and global economic recession caused activity changes in various sectors.^8−11^ Specifically, the UK’s first compulsory national lockdown on 23 March 2020 led to an immediate decrease in transport demand and public transport provision in London: by up to 65% for road traffic, 15% for bus service provision, and 59% for London Underground service provision.^8^ Meanwhile, both residential activities and home delivery services in London increased: the share of workers working from home full-time increased from 4% prepandemic to 54% in March and April 2020, and the proportion of retail sales completed online increased from 20% in 2019 to 33% by May 2020.^8^ While overall trends in activities were evident, heterogeneous responses were observed across vehicle type, spatial location, and population group.^8^ Societal trends such as remote working and e-commerce are expected to persist even after the pandemic.^8,12−14^

Activity changes during the COVID-19 lockdown provide an unprecedented opportunity to understand the likely effectiveness of prospective emission control policies and mobility patterns (such as higher levels of fleet electrification and more remote working) in improving air quality. A growing number of studies have focused on evaluating the air quality impacts of COVID-19 lockdowns. Most results show a significant reduction in pollutants that are largely affected by transport activities, such as NO_2_, due to the stringent travel restrictions during the lockdown; however, for other pollutants such as PM_2.5_ and O_3_, the results are less significant and more mixed.^15−20^ For example, Venter et al.^19^ analyzed the air quality levels across 34 countries worldwide and found that lockdowns reduced NO_2_ and PM_2.5_ concentrations by 60% and 31% on average, with a mixed effect on O_3_, ranging from −2% to 10%. Furthermore, several studies found lockdowns had heterogeneous effects on air quality across industrial structure, population size, transport demand, and geographical location.^21−26^ However, most of these studies focused on depicting the variation in effects across subsamples of cities or countries; few compared effects within a city or further evaluated the contribution of different factors to the heterogeneity of effects.

Several studies reported the lockdown impacts by comparing air quality before and after the lockdown.^15,16,27−29^ However, the impact estimates from this approach are typically influenced by several factors, such as weather conditions, seasonality effects, and a long-term trend in air quality, which has been improving in London.^30^ Some other studies quantified the impacts using a bottom-up simulation.^17,18,31,32^ However, simulation typically requires detailed activity/emission data, and the results are highly dependent on input scenarios, emission factors, and substantial assumptions in variables. Additionally, several studies evaluated the impacts with predictions of business-as-usual concentrations from statistical or ML models.^15,19,33,34^ However, this approach is subject to the generalization performance of the predictive model.

Causal inference methods generally have advantages for intervention evaluation, in terms of data requirements, model building, and the interpretation of effect estimates. *Causality* goes beyond statistical *association* by measuring the net effect of an intervention on an outcome through all pathways.^35^ Causal inference methods have already been applied to quantify the effect of COVID-19 lockdowns on air pollution.^20,22,23,36,37^ In these studies, sharp regression discontinuity design (RDD) is one of the main methods applied, and some important confounders (such as weather conditions and seasonality effects) were commonly controlled for by parametrically incorporating relevant variables into the model. However, due to the complexity of atmospheric processes, using a parametric confounding control for weather conditions in an air quality appraisal can be difficult.^38^ An alternative is to first use a nonparametric model to “de-weather” the pollution data.^30,38,39^

In this paper, we provide a causal analysis of the first UK national lockdown with a sharp RDD model to evaluate its causal impacts on London’s air quality. The meteorological normalization technique is applied to provide a nonparametric confounding control for weather conditions and seasonality effects. To further understand the spatial heterogeneity shown in the lockdown impacts, we additionally evaluate the contribution of different factors to the magnitude of lockdown impacts by interpreting a ML model with Shapley Additive exPlanations (SHAP) values.^40,41^ Various factors are considered, such as demographics, economy, transport demand, public transport supply, and geographical location. The identification of key factors affecting the level of pollution reduction within a city can provide further guidance in improving air quality and help shape our city with better and more equitable urban design, planning, and management.

## Materials and Methods

### Case Study Specification and Data Description

To quantify the causal effects of the first UK national lockdown on London’s air quality, a sharp RDD model is applied at individual air quality monitoring sites within the geographical extent of the Greater London Authority (GLA), for regulated air pollutants including NO_2_, O_3_, PM_2.5_, and PM_10_, and a nonregulated pollutant, nitrogen oxides (NO_*x*_ = NO + NO_2_).

Hourly air pollutant concentrations from 2016-01-01 to 2020-12-31 at monitoring sites are downloaded from the London Air Quality Network (LAQN) and the Air Quality England Network (AQE).^42,43^ Hourly meteorological observations for the same period are from the U.S. National Oceanic and Atmospheric Administration (NOAA).^44,45^ Details on data description, data merging, and data quality control are provided in SI §S1. Roadside and background monitoring sites are distinguished in the analysis. A roadside site is generally installed close to a busy road to represent roadside public exposure. A background site is located away from major emission sources to represent general public exposure at the city level.^46^ After data merging and quality reviewing, 42 background sites (LAQN: 29; AQE: 13) and 50 roadside sites (LAQN: 37; AQE: 13) are included in the study. The spatial distribution of these sites is illustrated in SI §S1.

To further interpret the spatial heterogeneity of lockdown impacts, various spatial features are evaluated based on their contribution in predicting the pollution reduction caused by the lockdown, including the distance to town centers and London Heathrow Airport (LHR), population, employment, median earnings, business count, business turnover, public transport accessibility level, annual average daily flow (AADF), and motor vehicle traffic. Complete information for the features, relevant data, and methods to assign feature values to different spatial locations can be found in SI §S1. 124 of the 136 features are included in the analysis after applying the data quality criteria (see SI §S1). The feature evaluation is conducted at the Middle Layer Super Output Area (MSOA) level. The GLA covers 983 MSOAs. MSOAs are small enough to indicate the spatial variation of features, and the total number is large enough to build a predictive model using ML methods.

### Model for Air Quality Impacts

The methodology to quantify the causal air quality impacts of an intervention with an explicit start time follows Ma et al.^30^ and sequentially combines meteorological normalization, change point detection (CPD), and a sharp RDD model. The research framework and a pseudo code outlining this method are shown in SI §S2. The sharp RDD is a causal inference approach for nonrandomized interventions, which assumes that the intervention is assigned by the value of a forcing variable on either side of a threshold.^47^ The core of the methodology in Ma et al.^30^ is a sharp RDD with time as the forcing variable and the start of the intervention, *T*_0_, as the threshold.

First, meteorological normalization is applied to control for variables (weather conditions and seasonality effects) that may violate the continuity assumption of a valid RDD.^39,48^ A normalized air pollutant concentration time series is derived by removing the variation in the observed concentrations that can be explained by these factors. Meteorological variables included in the model include temperature, wind speed, wind direction, atmospheric pressure, relative humidity, rainfall, and Monin–Obukhov length. Seasonality variables include the hours of the day, days of the week, and days of the year. A time variable is additionally included to represent the long-term concentration trend. A nonparametric model is used to consider the complex relationship between air pollution and model variables. Further details on model training and normalizing are provided in SI §S3. We additionally utilize a bootstrapping approach to evaluate the uncertainty in air quality effect estimates on meteorological normalization; see SI §S3. For most of the randomly selected test cases, the baseline effect estimate is generally similar to those from bootstrapping, indicating the robustness of our effect estimates.

Second, CPD is conducted to detect structural changes in the normalized concentration time series. Structural changes are identified as a change in the slope of the linear trend or an abrupt discontinuity in normalized concentrations. The detected change points are used to test the discontinuity in the normalized concentration (outcome) time series around *T*_0_ (threshold), justifying the use of sharp RDD. Particularly, a normalized concentration time series is considered to have responded to the lockdown if it has detected change point(s) that lie within a short period around *T*_0_, referred to as the “margin period” (MP). The MP is specified based on an analysis of the timing of response for different air pollutants (SI §S7). Moreover, CPD results are also used to support the research period specification of the RDD models. To mitigate influences from unobservable confounders and unrelated interventions, the normalized concentration time series are segmented by the detected change points, and only the data in the segments near *T*_0_ are used to estimate the RDD model. Detailed discussions on the CPD method and research period of RDD models are in SI §S4 and §S6, respectively.

Finally, a sharp RDD model is specified on the daily average normalized concentrations where a monitoring site showed a response. The model specification follows Ma et al.^30^ with further details in SI §S5. The model is based on a trend function approximation and quantifies the causal effect at the discontinuity of a trendline either side of *T*_0_, making it less vulnerable to random noise and unrelated events.^30^ Moreover, to account for any anticipation, adaptation, or delay in response around *T*_0_, the model is specified as a “donut” RDD, where the data within a short period around *T*_0_ (“donut hole”) are excluded from the RDD model estimation. For simplicity, the donut hole is specified as the same as MP. A summary of the key dates used in our study is provided in SI §S2. The interaction among different steps of this method is also illustrated in SI §S2.

The derivation of the lockdown effect, τ, is based on the estimated coefficients of the RDD model, which incorporates the impact from the current daily period and those from previous daily periods (i.e., a total effect); see SI §S5. By using the natural logarithm transformation of the outcome variable as the dependent variable, τ can be interpreted as the percentage change in daily average concentration caused by the lockdown.^39^ The interval estimates of τ are provided with a Monte Carlo simulation in Ma et al.^39^ to account for the uncertainty in estimating model coefficients. The statistical significance of τ at the 10%, 5%, and 1% levels is, respectively, determined if the corresponding confidence interval (CI) does not contain zero. This paper mainly discusses the statistical significance of τ at the 10% level.

Furthermore, we use a bootstrapping approach in Ma et al.^39^ to aggregate the effect estimates for each air pollutant across different monitoring sites to assess the overall air quality effects of the lockdown in London. We distinguish between roadside and background sites during the aggregation and further present results for the aggregation of only those sites that showed a response to the lockdown.

### Model for Feature Evaluation

To provide insight for future public policies, we identify the important contributing factors to the magnitude of lockdown impact at different locations with an explainable ML.

In this paper, we focus on the changes in long-term average (annual mean) background concentrations of NO_2_ in 2020 that can be attributed to the lockdown. We particularly focus on the reductions in background concentrations, as they are more representative of general public exposure compared to roadside concentrations.^46^ The European Union has established a limit value for annual average NO_2_ concentrations, which the member states should not exceed, and this limit value has been transposed into UK legislation.^49^ Although substantially reduced, the number of people in London living in areas exceeding the annual average legal limit for NO_2_ was still 119,000 in 2019.^50^ We note that ground-level O_3_ and PM_2.5_ also have noticeable adverse effects on human health.^51^ However, the sites that had measurements for these pollutants are relatively limited and spatially sparse compared with NO_2_, making it difficult to conduct a robust feature evaluation using a ML approach. Therefore, these pollutants are not included in further analysis. Moreover, our results show that concentrations of O_3_ and PM_2.5_ were less affected by the lockdown compared with NO_2_.

#### Observed and Counterfactual Concentrations

The annual average NO_2_ concentrations in 2020 at individual monitoring sites are estimated using observed concentrations for the with-lockdown scenario and counterfactual concentrations for the without-lockdown scenario. The counterfactual concentrations after *T*_0_ are scaled from the observed concentrations with the effect estimate τ̂. For the sites that showed a response to the lockdown, τ̂ uses the central estimate of the interval estimate of τ with the statistically insignificant τ̂ (at the 10% level) adjusted to zero; for the sites that showed no response, set τ̂ = 0. We denote *Y̅*_*s*_^obs^ and *Y̅*_*s*_^counter^ as the annual average NO_2_ concentration at site *s* with and without the lockdown, respectively. We denote *φ*_*s*_ = *Y̅*_*s*_^obs^ − *Y̅*_*s*_^counter^ and φ_%,*s*_ = (*Y̅*_*s*_^obs^ – *Y̅*_*s*_^counter^)/*Y̅*_*s*_^counter^ as the corresponding lockdown impact in absolute and relative terms, respectively.

#### Mapping Lockdown Impacts

To estimate the lockdown impacts at individual MSOAs, a mapping method is applied to separately interpolate {*φ*_*s*_} and {φ_%,*s*_} to the 1 × 1 km grids, which are then averaged to the MSOA level. The mapping method mainly follows Horálek et al.^52,53^ Ultimately, it combines a linear regression and ordinary kriging of the residuals from the linear regression (“residual kriging”). Further discussions on the mapping method and mapping results are listed in SI §S8 and §S9, respectively.

#### Measuring Feature Contribution

Tree-based ML models are typical ensemble methods for feature importance evaluation and have been applied in air quality analysis.^30,34,38^ In this paper, we build a gradient boosting decision trees (GBDT) model on the estimated pollution reductions in annual average NO_2_ concentrations attributable to the lockdown at different MSOAs, separately for absolute and relative reductions. Each GBDT model includes 124 features, covering various aspects of specific MSOAs (cf., SI §S1). The GBDT model training and hyperparameter tuning are discussed in SI §S10.

Some widely used feature importance metrics for a tree-based model, such as total gains and total splits, either rely on the frequency of usage in the model or focus on the contribution of a particular feature to error reduction.^54^ However, the value of feature importance provided by these metrics does not directly indicate how the magnitude of the model output variable is affected, which is also of great interest to policymakers. To address this question, we use the SHAP values (an approximation of the Shapley values) to interpret our GBDT models. For comparison, we also provide the results for measuring feature importance with the total gains of splits that use the feature. The top features selected by using total gains are generally consistent with those identified by using SHAP values (see SI §S10).

The Shapley value is a key solution method in cooperative game theory to allocate the total payoff generated by a coalition of players to individual players according to their contributions.^55^ This method can also be classified as an *additive feature attribution method* for a local explanation of complex predictive models,^40^ where the input features act as the players and the output prediction as the total payoff. Specifically, an additive feature attribution method is characterized by assigning a contribution *ϕ*_*i*_ to each feature, with the sum of {*ϕ*_*i*_} approximating the original model prediction.^40^ Therefore, *ϕ*_*i*_ is in the same unit as the model’s output and, consequently, can provide a better interpretation compared with the use of total gains. Moreover, several methods for interpreting complex ML models have been recently developed,^56−58^ and many of them also fall into the class of additive feature attribution methods.^40^ Previous studies have shown that Shapley values have advantages over the other methods of this class, as they can provide a single unique solution to the problem of assigning contributions with desirable properties.^40,41^

As estimating Shapley values is computationally expensive,^41^ we use an efficient algorithm in the *SHAP* library^40,41^ in python to approximate the Shapley values, and the results are called “SHAP values” hereafter. Notably, {*ϕ*_*i*_} is a local explanation of the GBDT model, meaning that it is unique to a single instance *x* in the model (i.e., an MSOA).^40,41^ Therefore, we additionally estimate the global importance of a particular feature by the mean absolute SHAP value across all MSOAs.^54^ Further discussions on the Shapley values, the algorithm for computing SHAP values, and an illustrative interpretation of SHAP values are included in SI §S10.

## Results and Discussion

### COVID-19 Lockdown Impacts on Air Quality

**Figure 1:**
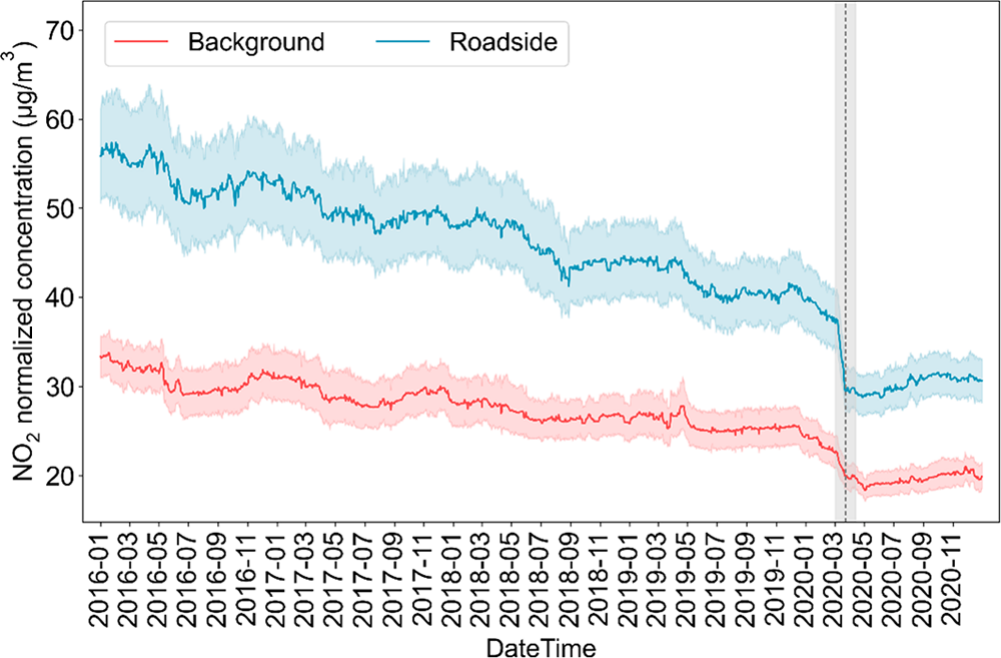
Daily average NO_2_ concentrations in London from 2016 to 2020 by monitoring locations (red: background sites; blue: roadside sites). Concentrations in London and the corresponding 95% CI are estimated by averaging across the concentrations at individual monitoring sites with bootstrapping. Air pollutant concentrations at individual monitoring sites are normalized to remove the influences of meteorological conditions and seasonality effects. The start of the lockdown is indicated by the vertical dashed line. The margin period, which is a symmetric period around the start of the lockdown for response identification, is shaded gray.

Around 50% of NO_2_ pollution in London comes from road transport.^50^ Following a dramatic decrease in road traffic led by travel restrictions, an abrupt decrease in NO_2_ concentrations was clearly observable in London around the start of the lockdown, at both the roadside and background monitoring sites (Figure 1). Examining changes in period mean concentrations at individual change points (see SI §S11), we find that the median of the concentration changes that occurred around the start of the lockdown was greater than the 90th percentile of changes observed at other times since 2016.

To further isolate the changes in air pollution attributable to the lockdown, we specify a sharp RDD model at individual monitoring sites, and the results are summarized across London in SI §S12. Our results indicate that 92% of the monitoring sites (background: 27/33; roadside: 45/45) within London showed a response in NO_2_ concentrations to the lockdown. The lockdown changed the daily average background NO_2_ concentrations by −7% [95% CI, −10%, −5%] on average, ranging from −23% to 0% at different sites, and roadside NO_2_ concentrations by −12% [−15%, −9%] on average, ranging from −50% to −2%.

Compared with background concentrations of NO_2_, at the roadside, the lockdown caused a higher average reduction (12%) and affected a higher proportion of monitoring sites (100%) (background: 7%, 82%). The difference in response between monitoring locations likely reflects how lockdown affected different activities: while roadside concentrations are mainly affected by transport emissions, background concentrations can be affected by various sectors, such as domestic heating, transport, industry, and energy supply.^46^ During the first UK national lockdown, mobility restrictions dramatically reduced London’s road traffic; however, activities in some other sectors in London were less affected (such as power generation) or even increased (such as residential activities).^8,59^

**Figure 2:**
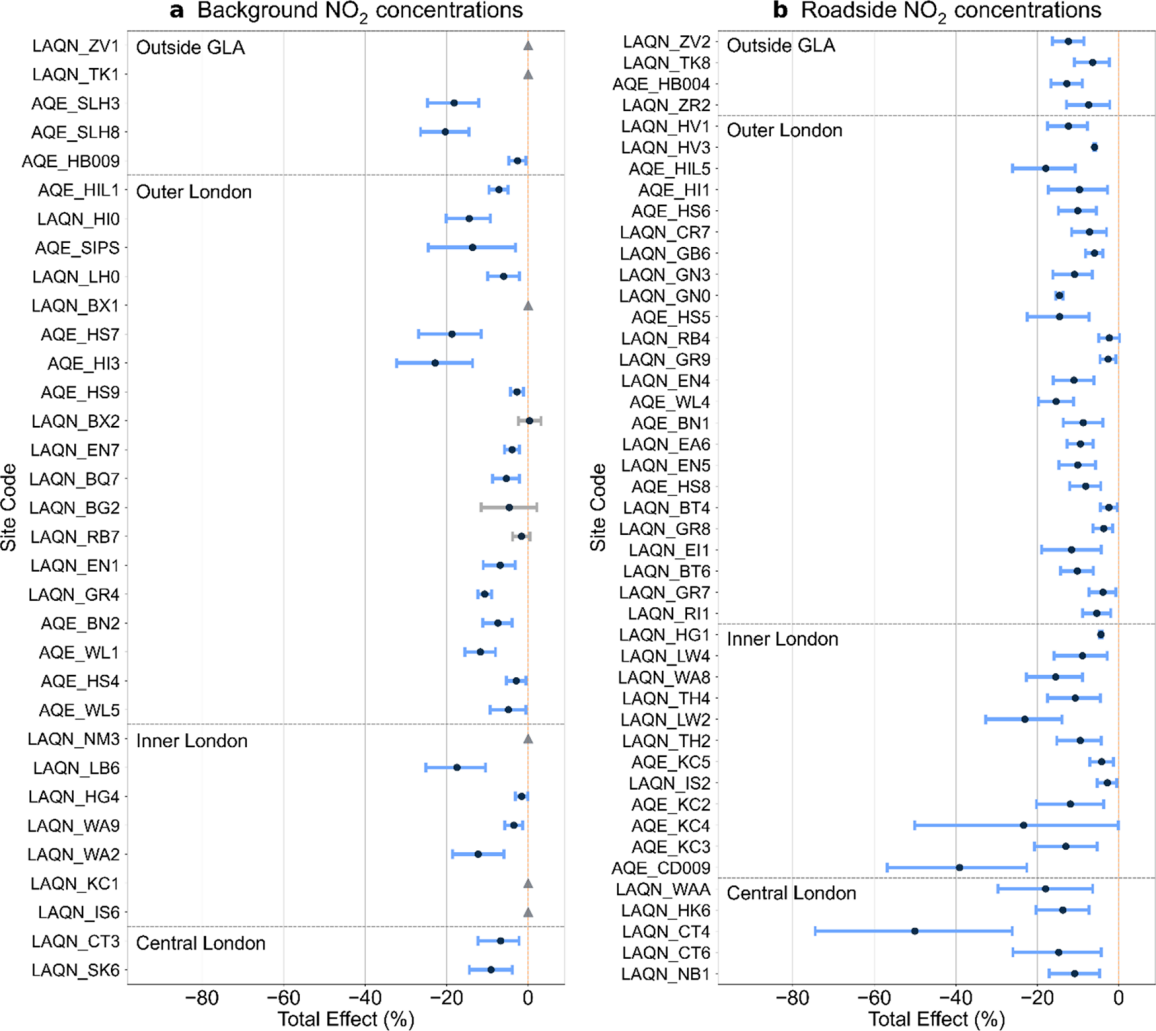
Estimated total effects on NO_2_ concentrations. Central estimates are indicated with black dots. The 95% CI is illustrated with uncertainty bars (blue: pollution reduction; red: pollution increase). Null responses are denoted by gray triangles. A detected response that is statistically insignificant (at the 10% level) is indicated with gray interval bars. Sites within central London, inner London, outer London, and outside GLA (from bottom to top) are separated by the gray horizontal dashed lines. Sites are sorted by the distance to the centroid of central London. Sites are labeled with data source and site code.

The lockdown consistently caused a significant reduction in NO_2_ concentrations at all roadside sites, yet the magnitude of the reduction was spatially heterogeneous (Figure 2). The highest reduction in roadside NO_2_ concentrations (50%; site LAQN_CT4) was observed in central London, and all of the roadside sites within central London showed a pollution decrease of greater than 10%. However, across the rest of London, 50% of the roadside sites showed a smaller pollution reduction of less than 10%. The spatial heterogeneity shown in the impact estimates for the roadside sites is generally consistent with the variation in road traffic reduction among different regions in London. Compared with inner and outer London, road traffic in central London experienced a higher initial reduction (64%) in the first week following the lockdown (inner and outer London: ∼50%) and showed a slower subsequent recovery.^8^

The response at background locations was similarly spatially heterogeneous. While the lockdown caused a large pollution reduction at some locations, the majority (∼70%) of background sites in London showed only a small pollution reduction (<10%) or null response. Moreover, the highest reduction in background NO_2_ concentrations (23%; site AQE_HI3) was in outer London, yet road traffic in outer London was generally less affected during the lockdown compared to central London;^8^ this reemphasizes that reductions in background concentrations of NO_2_ were not solely driven by changes in road traffic. Further discussions on the results for NO_2_ in central, inner, and outer London are provided in SI §S12.

Following the changes in road traffic, the UK reported an overall emission reduction in road transport of 25% for NO_*x*_ and 22% for particulate matter (PM) in 2020 compared with 2019.^60^ However, our results show that the relative change in London’s air pollution caused by the lockdown ranged from −62% to 0% for NO_*x*_, from 0% to 4% for O_3_, and from −5% to +0% for PM_10_, and there was no response for PM_2.5_. The negative correlation in the direction of changes in O_3_ (increase) and NO_2_ and NO_*x*_ (decrease) is consistent with theory and observations;^61^ effect estimates where these three pollutants are simultaneously measured are summarized in SI §S14. Aggregating the effects across London, the lockdown reduced NO_*x*_ concentrations by 11% at roadside sites and 6% at background sites on average and had an insignificant effect on the concentrations of O_3_, PM_10_, and PM_2.5_ concentrations. Unlike NO_2_ and NO_*x*_, the regional contribution to PM and O_3_ is substantial;^50,61^ more than half of the PM_2.5_ concentrations in London are from regional sources outside London.^50,62,63^ Our results imply that reducing transport activities and restricting exhaust emissions are not sufficient to tackle the challenges in reducing concentrations of air pollutants that are largely affected by regional emission sources. Further analysis of the changes in mean concentrations of NO_*x*_, O_3_, and PM at different periods since 2016 is provided in SI §S11, and further discussions on the estimated causal effects for these pollutants at different locations are in SI §S13, §S14, and §S15, respectively.

Our estimated air quality impacts of the lockdown are generally consistent with previous studies in that we find more marked reductions in NO_2_ concentrations yet less significant changes in other regulated pollutants (O_3_, PM_10_, and PM_2.5_). However, our impact estimates are not as large as those of Jephcote et al.,^15^ who reported average reductions of 38% and 17% respectively in NO_2_ and PM_2.5_ concentrations and an average increase in O_3_ concentrations of 8% across 129 monitoring sites in the UK during the first national lockdown. The impact estimates from Jephcote et al.^15^ were derived by comparing the air quality observations between the lockdown period and the same period in previous years. However, this approach may be biased by differing meteorological conditions and long-term trends in air quality. In particular, air quality in the UK (both NO_2_ and PM) has improved year on year in most major cities, including London.^30,49^ Therefore, the approach used in several studies to estimate the lockdown impacts in the UK by comparing air pollution levels across different years^15,16,64^ is likely to overestimate the air quality impacts attributable to the lockdown.

Another study by Shi et al.^65^ reported abrupt but smaller than expected changes in air quality attributable to the COVID-19 lockdown in 11 cities globally. This is in agreement with our results. For London, they found that the lockdown changed background concentrations of NO_2_, O_3_, and PM_2.5_ by −8 ± 8%, −2 ± 8%, and +11 ± 17%, respectively. While our results for NO_2_ and O_3_ are similar, the results for PM_2.5_ differ: we find no response for PM_2.5_. Shi et al.^65^ estimated the lockdown impact by the relative change in air pollution before and after the lockdown in 2020 after subtracting the relative change over the same period in the average concentrations across the previous 4 years. However, their estimated impacts for PM could be biased by PM episodes during their research period, between March and April in 2016–2020. PM episodes are a regular feature in springtime in Western Europe, when pollution from continental Europe is transported by southeasterly winds.^66^ Particularly, two PM episodes due to regional pollution transport were recorded in London in their specified postlockdown period.^67^ Although Shi et al.^65^ applied a meteorological normalization to control for weather conditions, this technique does not always sufficiently account for abrupt natural events or regional pollution transport.^65^ Consequently, the pollution increase estimated in Shi et al.^65^ for PM_2.5_ may incorporate the influence of these recorded episodes. In contrast, our study focuses on the time around the start of the lockdown; particularly, we use change point detection to identify the response and subsequently apply a sharp RDD to normalized concentrations near the start of the lockdown to estimate the effect, which in combination makes our method less susceptible to the influence of pollution episodes.

In addition to sharp RDD, difference-in-differences (DID) and synthetic control (SC) are commonly used causal inference approaches to estimate the air quality effects of COVID-19 lockdowns.^20,22,23^ Both DID and SC require defining treatment and control groups and pre- and postintervention periods, assuming only the treatment group in the postintervention period is exposed to the treatment.^47,68^ A crucial assumption in both methods is the parallel trend assumption, assuming the outcome of interest would follow the same trend in the treatment and control groups in the absence of the intervention.^47,69,70^ However, due to spatial correlations among different regions and pollution dispersion, changes in activities and pollution may extend beyond the area where the transport intervention is actually implemented.^30,71,72^ Therefore, selecting an appropriate control group for air quality appraisals using DID or SC can be challenging in practice, particularly in the case of nationwide COVID-19 lockdowns. In contrast, our research framework strengthens the justification of the key model assumptions of sharp RDD through the use of meteorological normalization and change point detection. These additional steps improve the validity of our method, resulting in a more reliable estimate of the causal effect.

Our methodology was designed to overcome existing limitations by enhancing the confounding control, improving the validity of causal effect estimates, and ensuring internal consistency within the research framework. However, it should be noted that this research framework is intrinsically a stochastic process; each component within the research framework may introduce uncertainties in the effect estimates. Sources of uncertainty can include air quality and weather observations, model fitting at different steps, and the meteorological normalization process. Future work should include a comprehensive quantification of uncertainties across different components within the research framework, in particular those associated with the meteorological normalization, as its output is specified as the outcome variable in the sharp RDD model and, consequently, directly influences the magnitude of effect estimates.

### Factors Affecting Lockdown Effects

The first UK national lockdown is estimated to have caused more significant, yet spatially heterogeneous, impacts on NO_2_ concentrations in London compared with other regulated pollutants included in our study (O_3_, PM_10_, and PM_2.5_). We now discuss the factors that contributed to the heterogeneous reductions in NO_2_ concentrations at different locations. All the factors refer to the spatial characteristics before the lockdown, as behavioral responses to the lockdown are more likely to be constrained by pre-existing conditions of the urban environment.

**Figure 3:**
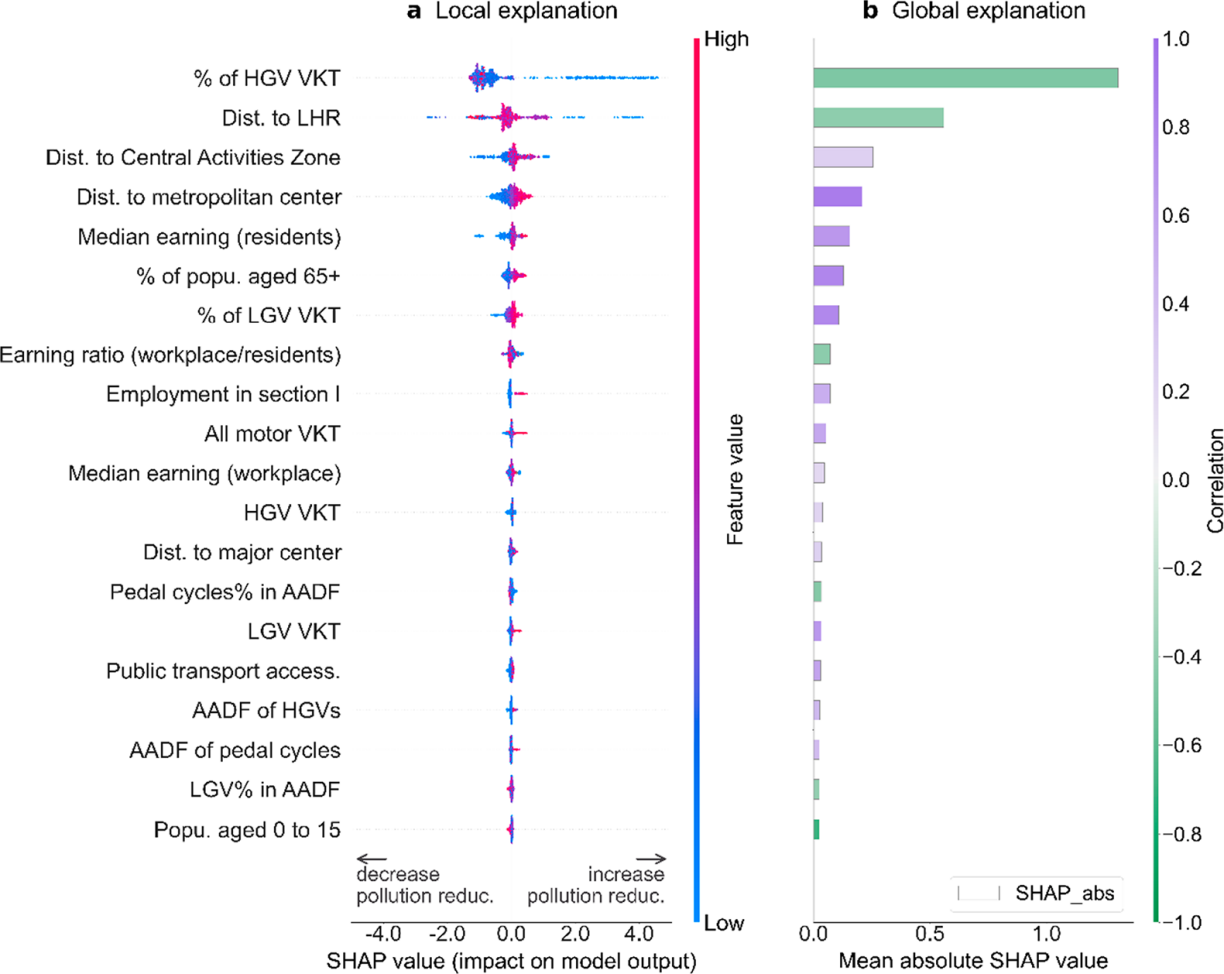
Estimated local SHAP values (left) and global feature importance (right) quantifying the contribution of each feature to the relative reduction (pp) in NO_2_ concentrations due to the COVID-19 lockdown. (**a**) Each point is a SHAP value specific to a feature and an MSOA; the color of a point indicates the feature’s value for that MSOA, and points are spread out in the y-dimension to avoid overlap. (**b**) The global importance of a particular feature is calculated as the mean absolute SHAP value across all the MSOAs; the color of bars indicates the Pearson correlation between the value of a feature and the corresponding SHAP value across different MSOAs: a negative correlation (green) indicates that an MSOA with a higher value of that feature is generally associated with a smaller pollution reduction, while a positive correlation (purple) indicates that an MSOA with a higher feature value is generally associated with a larger pollution reduction. Features are sorted by the global feature importance; the top 20 features with the most significant global importance value are plotted.

The estimated SHAP values are summarized in Figure 3 with respect to the relative pollution reduction and in SI §S10 for the absolute pollution reduction caused by the lockdown. In these figures, feature importance and feature effect are combined in the local SHAP values: a positive SHAP value of a feature at an MSOA indicates that this feature contributed to increasing the pollution reduction at this location, while a negative value implies a decrease in pollution reduction associated with this feature; the magnitude of SHAP values indicates how much the contribution was, and they are in the same units as the model output. For example, a value of −2 in Figure 3a would indicate that for that MSOA, this feature *decreased* the relative pollution reduction by two percentage points (pp).

**Figure 4:**
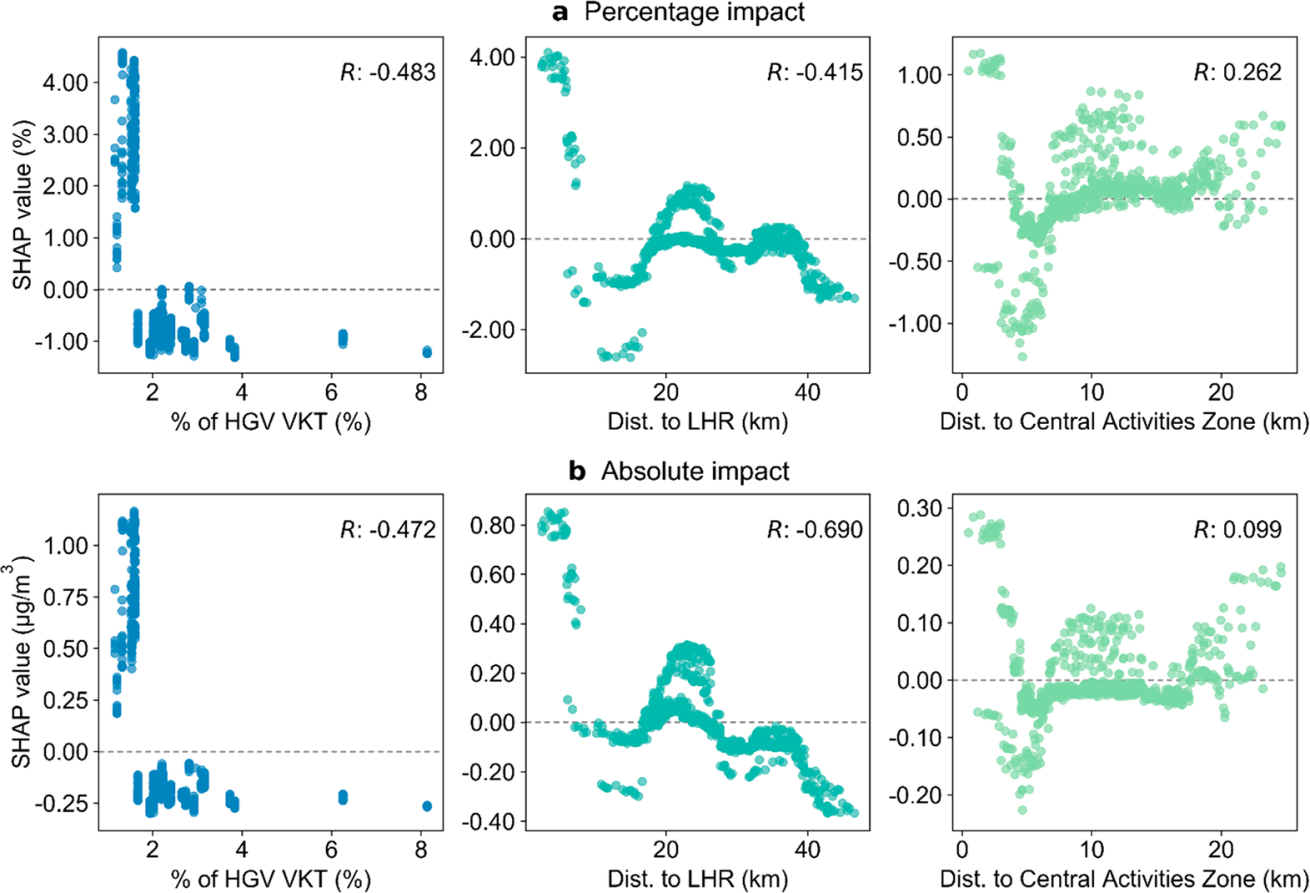
Relationship between the proportion of HGVs in road traffic, the distance to LHR, the distance to the Central Activities Zone, and the SHAP value for (**a**) relative lockdown impact (pp) and (**b**) absolute lockdown impact (μg/m^3^). Each point shows the value of the feature (*x*-axis) and the corresponding SHAP value (*y*-axis) at a particular MSOA. The sign of the SHAP value indicates how the value of that feature contributes to the prediction of the lockdown impact at that MSOA (positive: increasing the pollution reduction; negative: decreasing the pollution reduction). The Pearson correlation (*R*) between the value of the feature and the corresponding SHAP value across different MSOAs is labeled on each plot.

Our results show that the degree to which NO_2_ was reduced in an area was mostly correlated with the proportion of heavy goods vehicles (HGVs) in road traffic before the lockdown, the distance to LHR, and the distance to the Central Activities Zone (city center) (Figure 3 and SI §S10). This finding is generally consistent with Yang et al.,^34^ who found the NO_2_ reduction in Los Angeles during the lockdown was primarily due to the changes in HGVs’ activities; they also found the distance to major airports was an important predictor of NO_2_ concentrations in the city. In addition to feature ranking, our use of explainable ML enables us to find that a lower proportion of HGVs in road traffic and closer proximity to LHR are associated with greater pollution reductions in most MSOAs; however, different MSOAs can have opposite effects from a short distance to the city center (Figure 4). The local SHAP values for a particular MSOA are discussed in SI §S16 as an example to further demonstrate how to use an explainable ML for site-specific attribution. The results indicate that a small proportion of HGVs, a moderate distance to LHR, and a moderate distance to the city center increased the pollution reduction at this place by 4.3 pp, 1.1 pp, and 0.7 pp, respectively.

HGVs accounted for 3% of London’s on-road vehicle fleet yet disproportionately contributed 13% of road transport NO_*x*_ emissions in 2019.^73,74^ Compared with other types of road traffic, London’s road freight showed a lower initial reduction (∼50%) in HGVs and light goods vehicles in the first week following the lockdown (60% in cars) and a quicker subsequent recovery.^8^ Our results show that where HGVs comprised less than 1.6% of vehicle traffic, the relative pollution reduction increased by up to 4.6 pp (Figure 4a). Moreover, the local SHAP values of this feature are all distinct from zero for the absolute lockdown impact (Figure 4b), indicating that this feature is important for predicting the absolute pollution reduction at all locations. These results highlight the importance of controlling emissions from road freight transport. In particular, studies suggest the shift toward e-commerce is likely to persist beyond the lockdown,^8,10,14^ which potentially lead to further growth in road freight transport in the future.^75^ In addition to the fleet composition features like the HGV proportion in road traffic, the vehicle kilometers traveled (VKT) for all motor vehicles is also highlighted in our study (Figure 3 and SI §S10), which implies the necessity of managing overall traffic demand in addition to reducing emission factors.

**Figure 5:**
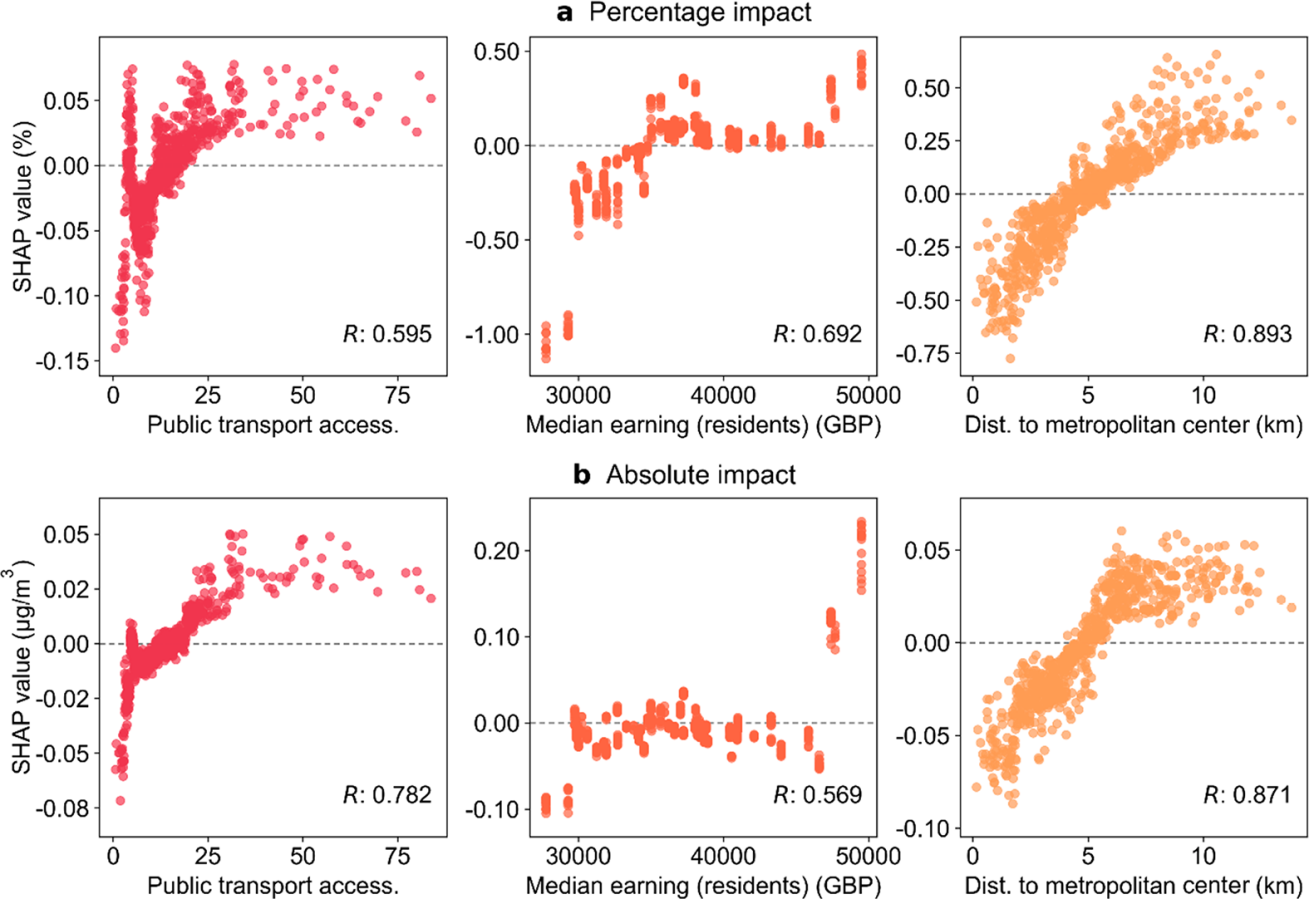
Relationship between public transport accessibility level, median gross annual pay for residents, the distance to the nearest metropolitan center, and the SHAP value for (**a**) the relative lockdown impact (pp) and (**b**) the absolute lockdown impact (μg/m^3^). Each point shows the value of the feature (*x*-axis) and the corresponding SHAP value (*y*-axis) at a particular MSOA. The sign of the SHAP value indicates how the value of that feature contributes to the prediction of the lockdown impact at that MSOA (positive: increasing the pollution reduction; negative: decreasing the pollution reduction). The Pearson correlation (*R*) between the value of the feature and the corresponding SHAP value across different MSOAs is labeled on each plot.

The lockdown affected different people differently. The median gross annual pay for residents is found to be an important factor for the level of lockdown impacts, as shown in Figure 3 and SI §S10. Additionally, our results reveal that the existing health inequalities within the city were exacerbated during the lockdown: areas with lower-income residents, which have also been exposed to higher levels of air pollution before the pandemic,^76^ typically experienced smaller NO_2_ pollution reductions during the lockdown. Areas with median incomes below the 10th percentile experienced a mean decrease in pollution reduction of 0.6 pp, while those with median incomes above the 90th percentile experienced a mean increase in pollution reduction of 0.3 pp (Figure 5). Our results are generally consistent with a previous study,^77^ which showed the affluent residential neighborhoods in London had more reductions in activity than the city average during the lockdown. The divide in activity changes and pollution reductions during the lockdown between earning levels may reveal the divide in occupations. Specifically, people with lower incomes were less likely to work from home during the lockdown, and their jobs also have less potential to adopt teleworking in the future.^8,12,78^ Moreover, we find places with public transport accessibility levels above the median were generally associated with a larger pollution reduction, while those with accessibility levels below the median typically experienced a smaller pollution reduction, both by up to 0.1 pp (Figure 5). Therefore, our results highlight the link between existing social inequalities and the effects of air pollution control policies.

The COVID-19 pandemic and subsequent lockdowns have also seen a dramatic decrease in the global air traffic. Some large improvements in NO_2_ and NO_*x*_ concentrations, respectively of up to 23% and 33%, attributable to the lockdown were found at background monitoring sites around LHR (cf. Figure 2 and SI §S13). Sites closer to the LHR experienced larger pollution reductions; a distance less than 5.9 km to the LHR is estimated to have increased the relative pollution reduction by up to 4.1 pp (Figure 4). As the UK’s busiest airport, LHR served 45% of air passengers in London before the pandemic.^79^ However, LHR reported a significant decrease in passenger demand after the lockdown; by ∼97% in April 2020 compared with the same month in 2019.^80^ Our impact estimates at individual monitoring sites around LHR are generally consistent with several previous studies, which estimated LHR contributed to 4%–31% of NO_*x*_ concentrations^81^ and 4%–36% of NO_2_ concentrations^82,83^ in nearby areas. Notably, our impact estimates are likely a combined effect of emission reductions from both airside activities and on-road traffic associated with passengers, freight, and supporting services.

Furthermore, as most residents were forced to stay close to home, people tended to make shorter and more local trips during the lockdown.^8^ While close proximity to London’s Central Activities Zone is estimated to enhance the reduction in NO_2_ in several cases (Figure 4), the areas that are closer to metropolitan centers were commonly less affected compared with the areas further away (Figure 3). As shown in Figure 5, we find a strong positive correlation between the distance to the nearest metropolitan center and its corresponding SHAP value (correlation coefficient ∼0.9), however, the influence of this feature on the magnitude of lockdown impacts was all within 1 pp. Metropolitan centers have good accessibility and can serve a large catchment with significant employment, service, and leisure functions.^84^ These results imply the potential of alleviating air pollution in the city center by shifting activity to metropolitan centers, however, policymakers should be mindful of the potential deterioration of air quality within and around metropolitan centers following the development. We note that continued remote or hybrid working post-pandemic may^8,12,13^ persisently change mobility patterns compared to pre-pandemic, thereby contributing to a shift in activity away from the city center.^8^

## Implications

Our study shows that the unprecedented decrease in transport activities following the COVID-19 lockdown did not lead to a commensurate reduction in air pollution. The heterogeneous effects observed across air pollutants and spatial locations suggest that reducing transport activities and restricting exhaust emissions, such as through remote working and fleet electrification, alone are not sufficient to address the complexities of improving air quality, particularly for pollution at background locations and pollutants that are largely affected by regional emission sources. Therefore, improving air quality across the city requires a multifaceted set of policies to control emissions across sectors, with coordination among governments and consideration of existing inequalities. Within the transport sector, sustained efforts are necessary such as reducing emissions for freight transport, facilitating and encouraging public transport, and integrating transport planning with land use planning. Meanwhile, proactive actions are necessary to respond to long-term societal trends and to consistently guide cities for health and equality.

## Data Availability

The data that support the findings of this study are openly available at the following URL: 10.5281/zenodo.7774628.

## Supporting Information Available

Data description and processing, graphical summary of research framework and summary of key dates, meteorological normalization method and uncertainty analysis, change point detection method, details on sharp RDD model and derivation of total effect, length of research period of RDD models, timing of response to the lockdown for different air pollutants, mapping method and results, method and results for feature evaluation using total gains and SHAP values, concentration changes in the historical context, estimated effects for different air pollutants, and example of site-specific attribution (PDF)
