# Supplementary material for: Using Explainable Machine Learning to Interpret the Effects of Policies on Air Pollution: COVID-19 Lockdown in London
Source: Environ Sci Technol. 2023 Aug 11;57(46):18271–81. doi: 10.1021/acs.est.2c09596 (PMC10666281; doi:10.1021/acs.est.2c09596)
Supplement: Supplementary file 1 — es2c09596_si_001.pdf [file es2c09596_si_001.pdf]

Supporting Information for

Using explainable machine learning to interpret the effects of policies on air pollution: COVID-19 lockdown in London

Liang Ma, Daniel J. Graham and Marc E. J. Stettler*

*Corresponding author: Marc E. J. Stettler
Email: m.stettler@imperial.ac.uk

This PDF file includes:

Supporting text: 82 pages

Number of Figures: 31

Number of Tables: 18

Supporting Information

S1. Context of the study, data description, and data quality control

S1.1 COVID-19 lockdown in the UK

The UK confirmed its first two COVID-19 cases at the end of January 2020, after which case numbers rose rapidly, to 58 at the end of February, and to over 25,000 by the end of March[1]. Consequently, the UK government advised everyone against nonessential travel and to work from home where possible on 16 March, followed by a compulsory national lockdown on 23 March 2020. Requirements imposed during the lockdown included: (i) requiring people to stay at home, except for limited purposes; (ii) closing certain nonessential businesses and venues, and (iii) prohibiting all gatherings of more than two people in public. The lockdown measures started to ease gradually from 10 May 2020 and activities began to recover. As infections rose again, some restrictions were again imposed in September and a second compulsory national lockdown was implemented on 5 November 2020[1].

Traffic volume and retail footfall in London decreased immediately after the first UK national lockdown, by 65% and 80% respectively[1]. Public transport demand decreased by up to 86% for bus services and up to 97% for London Underground. In response, public transport service provision was reduced by 15% for bus services and up to 59% for London Underground services[1]. Meanwhile, both residential activities and home delivery services increased in London during the lockdown: the share of workers working from home full-time increased from 4% before the pandemic to 54% in March and April 2020, and the proportion of retail sales completed online increased from 20% in 2019 to 33% by May 2020[1].

S1.2 Case study specification

The research area for the analysis of causal air quality impact is defined by the geographical extent of the Greater London Authority (GLA); the area is further classified into central, inner, and outer London, and the region outside the GLA, which are referred to as “subregions” in the paper. Central London is defined by the Central Activities Zone in the London Plan[2]; the boundary of inner and outer London is defined by the Office for National Statistics of the UK[3]. The research area for the feature evaluation is defined by the boundary of the GLA. The start of the first UK national lockdown, 2020-03-23, is defined as the start of the intervention.

S1.3 Data for air quality effect estimation

Data from 2016-01-01 to 2020-12-31 are used. Hourly air pollutant concentrations at monitoring sites are downloaded from the open-source data in the London Air Quality Network (LAQN) and the Air Quality England Network (AQE)[4,5]. The duplication of measurements between these two data sources is checked based on metadata and data coverage. A monitoring site in the AQE is only considered for further analysis if it has no counterparts in the LAQN; a counterpart is identified based on geographical coordinates

(rounded to 5 decimals), opening date, closing date, and site type (roadside/background). For each remaining monitoring site (LAQN/AQE), data coverage within the global research period (2016-01-01 to 2020-12-31) is calculated separately for each air pollutant. The AQE data for pollutant p at site s is further excluded if there is a counterpart in the LAQN that has: (i) the same geographical coordinates (rounded to 5 decimals), opening date, and site type as site s, and (ii) the same data coverage (rounded to 3 decimals) for pollutant p. After merging the data from two monitoring networks, the air quality data is further reviewed based on data quality criteria specific to the case study; a monitoring site is included in the analysis for a particular pollutant only if at least 80% of the daily intervals have valid daily average data on both sides of the start of the lockdown. A daily average is considered valid only if at least 75% of hourly intervals within the day have complete observations. After data merging and quality reviewing, 42 background sites (LAQN: 29; AQE: 13) and 50 roadside sites (LAQN: 37; AQE: 13) are included in the study. The spatial distribution of these sites is illustrated in Figure S1-1 for background sites and Figure S1-2 for roadside sites that have measurements for NO_x, NO_2, or O_3; Figure S1-3 and Figure S1-4 provide the corresponding information on the sites that have measurements for PM_{10} or $PM_{2.5}$.

Hourly temperature (℃), wind speed (m/s), wind direction (°), and atmospheric pressure (hPa) are observations from the Integrated Surface Database (ISD) of the U.S. National Oceanic and Atmospheric Administration (NOAA)[6]. Hourly relative humidity (%) is approximately calculated using the temperature and dew point observations in the ISD[*]. Hourly rainfall (mm) is estimated with the 12-hour and 6-hour total rainfall observations in the ISD by spreading the 6-hour total across the previous 6 hours[†]. In addition, Monin-Obukhov length (m) is considered to account for atmospheric stability. It is calculated based on land surface observations[6] and upper air radiosonde observations[7] with the meteorological pre-processor, AERMET, developed by the U.S. Environmental Protection Agency (EPA)[8]. London Heathrow is used for the land surface data and Herstmonceux West for the upper air radiosonde data.

---

[*]Relative humidity (RH) is approximately calculated with temperature and dew point by [65]: RH ≈ 100*$[(112 - 0.1 * \text{temperature} + \text{dew point})/(112 + 0.9 * \text{temperature})]^{8}$.

[†] The missing values in the 6-hour total rainfall observations are filled by the difference in total rainfall in the previous 12 hours and in the previous 6 hours, if both observations are available.

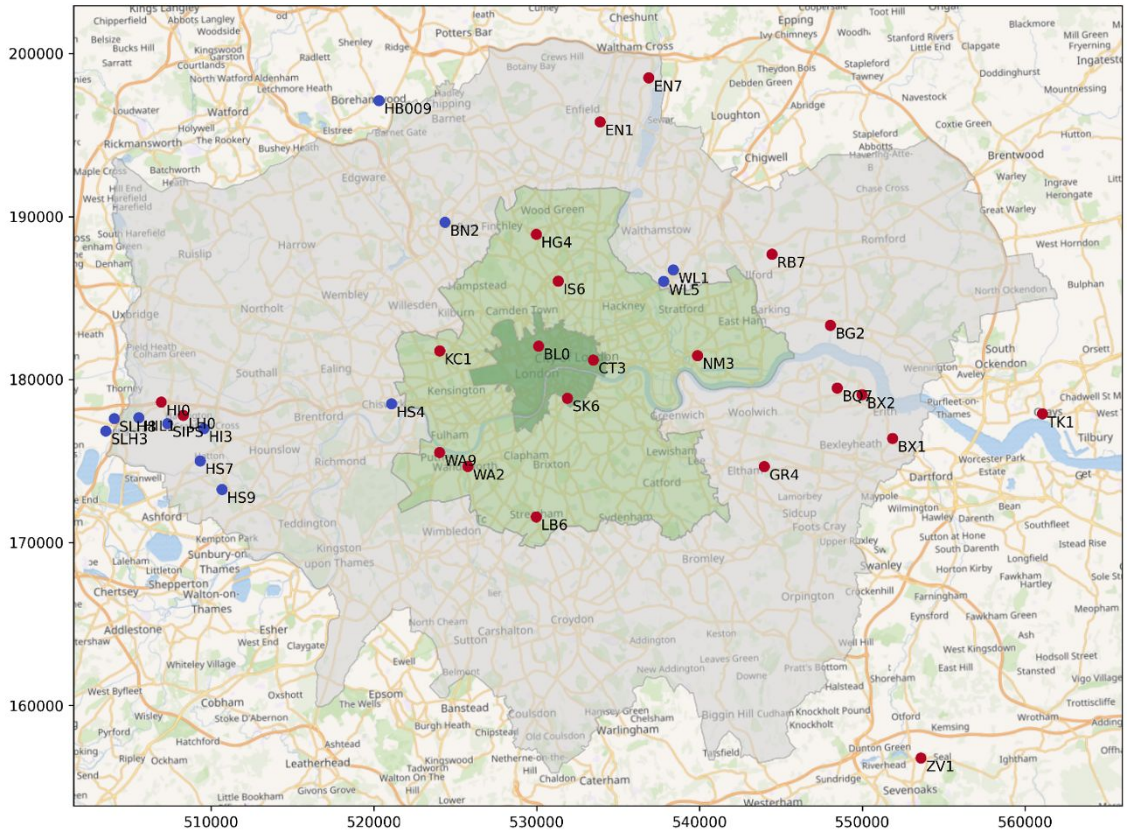

Figure S1-1. Location of background monitoring sites (red: LAQN sites; blue: AQE sites) included in the analysis for NO_2, NO_x, or O_3. The boundary of central London, or the Central Activities Zone, is shaded dark green. The boundary of inner and outer London is shaded light green and grey, respectively. This base map image has been obtained by the authors from the Wikimedia website where it was made available by OpenStreetMap contributors under a CC BY-SA 4.0 licence. It is included within this article on that basis. It is attributed to © OpenStreetMap contributors.

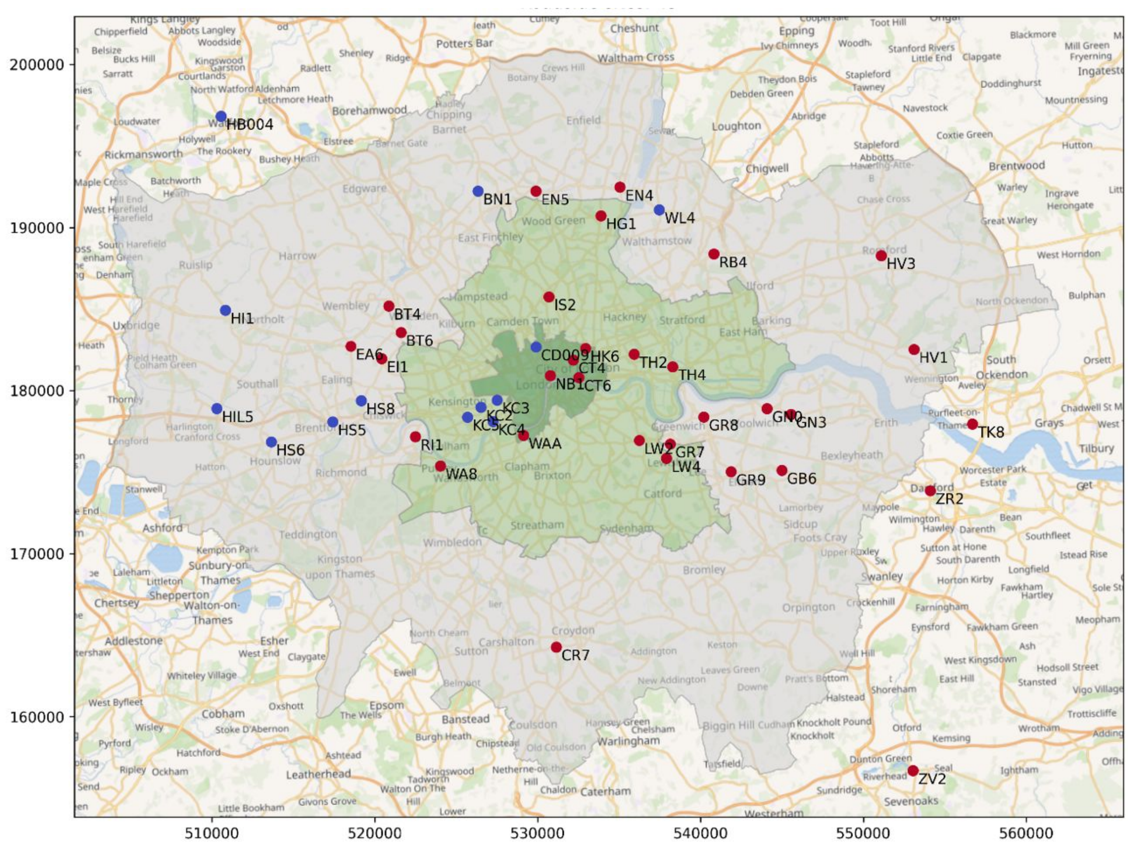

Figure S1-2. Location of road monitoring sites (red: LAQN sites; blue: AQE sites) included in the analysis for NO_2, NO_x, or O_3. The boundary of central London, or the Central Activities Zone, is shaded dark green. The boundary of inner and outer London is shaded light green and grey, respectively. This base map image has been obtained by the authors from the Wikimedia website where it was made available by OpenStreetMap contributors under a CC BY-SA 4.0 licence. It is included within this article on that basis. It is attributed to © OpenStreetMap contributors.

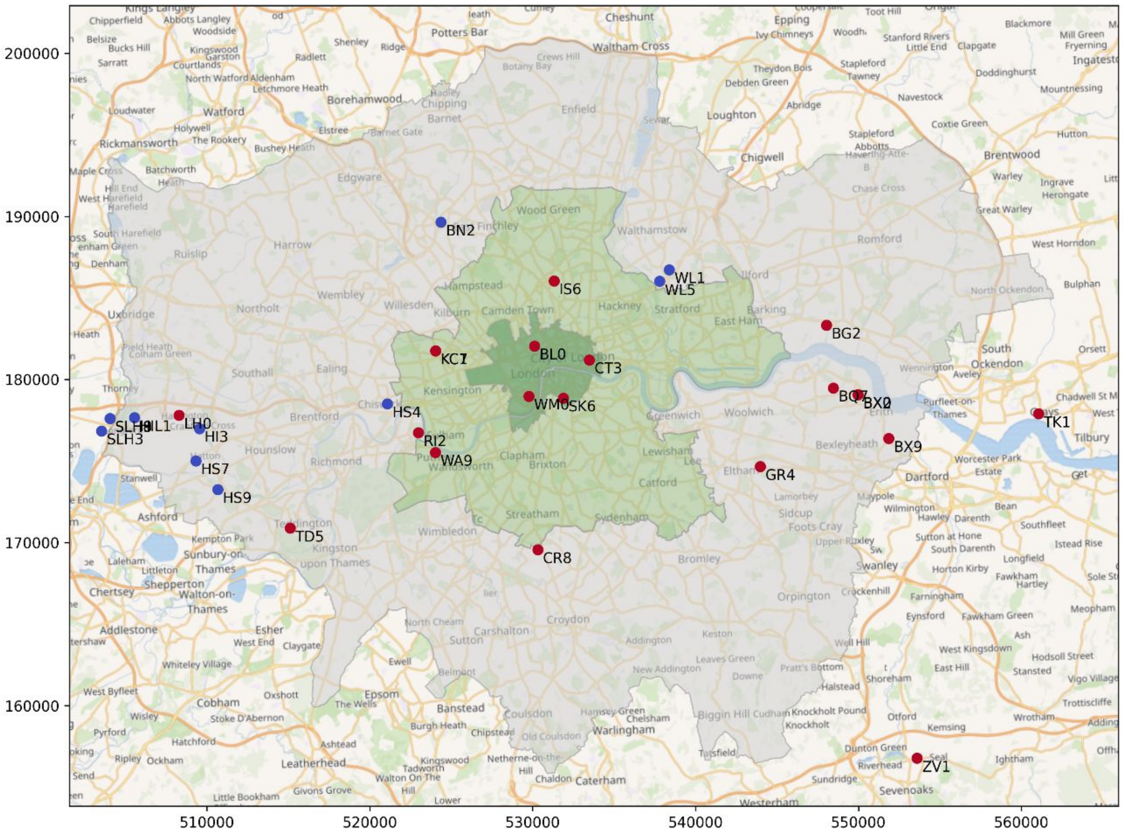

Figure S1-3. Location of background monitoring sites (red: LAQN sites; blue: AQE sites) included in the analysis for PM_{10} or $PM_{2.5}$. The boundary of central London, or the Central Activities Zone, is shaded dark green. The boundary of inner and outer London is shaded light green and grey, respectively. This base map image has been obtained by the authors from the Wikimedia website where it was made available by OpenStreetMap contributors under a CC BY-SA 4.0 licence. It is included within this article on that basis. It is attributed to © OpenStreetMap contributors.

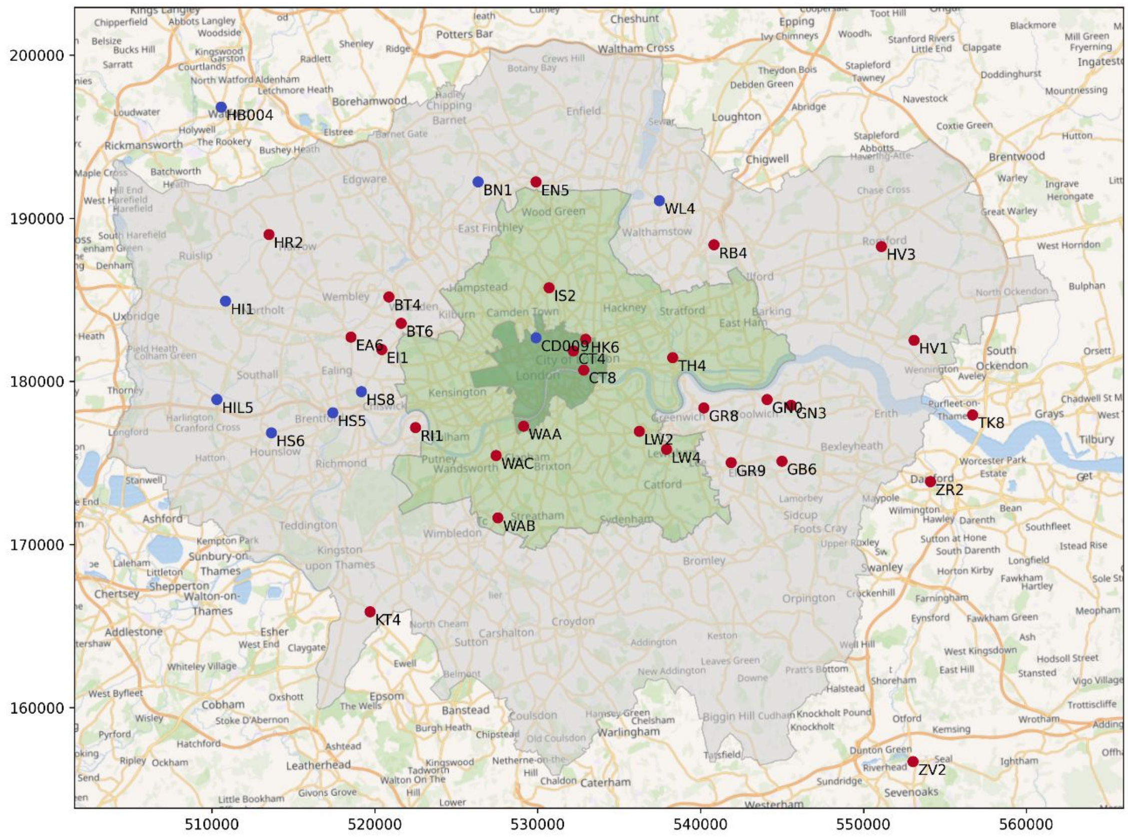

Figure S1-4. Location of roadside monitoring sites (red: LAQN sites; blue: AQE sites) included in the analysis for PM_{10} or $PM_{2.5}$. The boundary of central London, or the Central Activities Zone, is shaded dark green. The boundary of inner and outer London is shaded light green and grey, respectively. This base map image has been obtained by the authors from the Wikimedia website where it was made available by OpenStreetMap contributors under a CC BY-SA 4.0 licence. It is included within this article on that basis. It is attributed to © OpenStreetMap contributors.

S1.4 Data for feature contribution evaluation

The feature evaluation is conducted at the Middle Layer Super Output Area (MSOA) level. The data used for feature evaluation are from various sources. Table S1-1 shows the data for supplementary features, among which the important contributing factors are identified. For the features where the data source updates periodically, the data in 2019 (the year before the lockdown) is used. To allocate the value of features to specific MSOAs, we aggregate the feature value with a mean or median where the original data of a particular feature has more than one value within an MSOA (see details in Table S1-1); for any feature that has a lower spatial resolution, the value is assigned based on the location of the centroid of each MSOA. In addition, the supplementary features are individually screened based on data quality; a feature is included in the analysis only if at least 75% of the MSOAs have valid data. In total, 136 features are examined, of which 124 features meet the data quality criteria and 12 features (in the type of business count by employment size band and industry sector) are excluded for further analysis.

Table S1-2 shows the data used in the mapping model that interpolates the lockdown impact at individual monitoring sites to the unified grids.

Table S1-1. Spatial-temporal resolution and source of variables for spatial interpolation model.

Category	Number of features	Resolution of original data		Source [l]
		Spatial [k]	Temporal	
Distance to different town centres [a] and London Heathrow Airport	5	-	-	Greater London Authority[9]
Population by age group [b]	7	MSOA (983)	Annual	Office for National Statistics[10]
Employment by industry [c, d]	30	MSOA (983)	Annual	Office for National Statistics[11]
Median earnings by resident and workplace [e]	3	Parliamentary constituency (73)	Annual	Office for National Statistics[12,13]
Business counts by employment size band and industry sector [c, f]	65	MSOA (983)	Annual	Office for National Statistics[14]
Business turnover by industry sector [c, g]	9	MSOA (983)	Annual	Office for National Statistics[15]
Public transport accessibility level [h]	1	100-m grid (159,451)	-	Transport for London[16]
Annual average daily flow (AADF) by vehicle type [i]	9	Count point (1826)	Annual	Department of Transport[17]
Motor vehicle traffic by vehicle type [j]	7	Local authority (33)	Annual	Department of Transport[18]

(a) Town centre network is defined in London Plan 2021[2]. Central Activities Zone, metropolitan, major, and district town centres are considered. Features: distance (unit: km) to the nearest town centre of each type.

(b) Age group: Aged 0 to 15, 16 to 64, 65+. Features include the total population (unit: persons), the population in each age group, and the proportion of each age group in the total population.

(c) Industry section: 21 sections (highest level) in the UK Standard Industrial Classification (SIC) hierarchy. Industry sector: Agriculture, Production, Construction, and Service; aggregated based on SIC code.

(d) Features include total employment (unit: persons), employment in each industry section, employment in each industry sector, and the proportion of each industry sector in total employment.

(e) Median of gross annual pay. Unit: GBP. Features include resident, workplace, ratio (workplace/resident).

(f) Employment size band: Micro (0 to 9), Small (10 to 49), Medium-sized (50 to 249), Large (250+). Features include the business count in each subdivision, total business count in each employment size band, total business count in each industry sector, the proportion of each industry sector in each employment size band, and the proportion of each employment size band in each industry sector.

(g) Turnover is estimated by the midpoint of turnover size bands and the business count. Features include total turnover (unit: thousand GBP), turnover in each industry sector, and the proportion of each industry sector in total turnover.

(h) Grids are intersected with the MSOAs. The average value across the grids within each MSOA is used.

(i) Vehicle type: all motor, including car and taxi, heavy goods vehicle (HGV), and light goods vehicle (LGV); pedal cycle. Unit: number of vehicles. For each count point (road link), the proportion of motor vehicle type in all motor counts and the proportion of pedal cycle in total counts (all motor + pedal cycle) are estimated. The road links are intersected with the MSOAs. The median across the road links within each MSOA is used.

(j) Vehicle type: all motor, car, HGV, LGV. Unit: vehicle km. Features include traffic volume in each vehicle type, and the proportion of car, HGV, and LGV in all motor vehicles.
(k) The number of spatial units within the GLA is shown in the bracket.
(l) For the annually updated database, data in the year 2019 is used.

Table S1-2. Spatial-temporal resolution and source of variables for spatial interpolation model.

Variable	Data	Year of data used	Original resolution		Source
			Spatial	Temporal	
Wind speed	Mean wind speed at 10 m (knot)	2019	1-km grid	Annual	Met Office et al.[19]
Sunshine hours	Sunshine duration (hour)	2019	1-km grid	Annual	Met Office et al.[19]
Elevation	Elevation (m)	-	15-arcsec grid [(c)]	-	Danielson & Gesh[20]
Concentrations from chemical transport model (CTM) [(a)]	London Atmospheric Emissions Inventory (LAEI) concentrations ($\mu g/m^3$)	2016	20-m grid	-	LAEI[21]
	Pollution Climate Mapping (PCM) modelled concentrations ($\mu g/m^3$)	2016 [(b)]	1-km grid	Annual	Department for Environment, Food and Rural Affairs[22]

(a) Modelled concentrations use the values from LAEI where possible and those from PCM modelled concentrations otherwise. The LAEI data is downloaded at a spatial resolution of 20 m × 20 m and is averaged up to 1 km to be consistent with the PCM data.
(b) The data in 2016 is used to be consistent with the LAEI data.
(c) Approximately at a spatial resolution of 450 m × 450 m.

S2. Research framework and definition of key dates

This section provides a graphical overview of the research framework employed in our study, along with a table that summaries the definition of key dates used in our research.

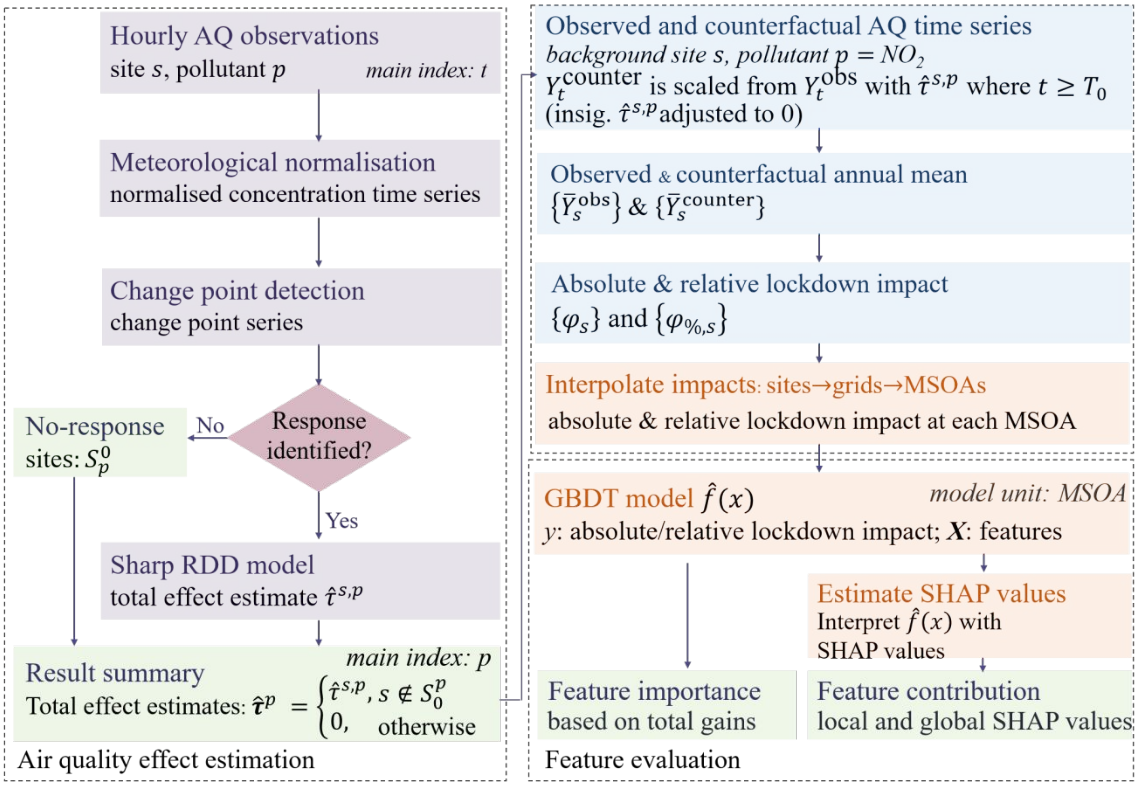

Figure S2-1. Graphical summary of the methodology for air quality effect estimation (left) and important contributing factor identification (right). The output/conclusion is coloured green. The left column (purple) goes through each air pollutant concentration time series. The processes in the right column are either conducted on each monitoring site (blue) or focused on Middle Layer Super Output Areas (orange).

Table S2-1. Summary of the key dates used in the study.

Key date	Definition	Usage	Specification
Start of lockdown (T_0)	The start of the first compulsory national COVID-19 lockdown in the UK<br>The start of the intervention	The threshold in the sharp RDD in time to determine the treatment indicator	2020-03-23
Margin period (MP)	A short period around the start of the lockdown, T_0, for response identification	A normalised concentration time series is considered to have responded to the lockdown if it has detected change point(s) that lie within the MP	3 weeks before and after the lockdown:<br>2020-03-02 to 2020-04-13
Research period of the RDD model	Data period selected for the RDD model estimation. Specific to each monitoring site	Mitigate influences from potential unobservable confounders and unrelated interventions:<br>only the segments of normalised concentration time series near the start of the lockdown are used to estimate the effect	Data-driven, depending on change point detection results at a particular site:<br>the start and end of the research period are respectively specified as the last change point before T_0 and the first change point after T_0 that are outside the MP and satisfying the data quality requirement of the time series segment. (Details in SI §S5.2)
Donut hole	A short period around the start of the lockdown, T_0, to account for any anticipation, adaptation, or delay in response to the lockdown	All data within the donut hole are excluded from the RDD model estimation	3 weeks before and after the lockdown:<br>2020-03-02 to 2020-04-13

Air Quality Effect Estimation

Input:

- Observed concentrations of pollutant p at site s from 2016-01-01 to 2020-12-31.
- Meteorological observations for London from 2016-01-01 to 2020-12-31.

Output:

- Estimated effect of the lockdown on pollutant p at site s, $\hat{\tau}^{s,p}$.

Step 1: Meteorological Normalisation

1.1 Base model:
Build a GBDT model on the observed concentration time series with 7 meteorological variables, 3 seasonality variables, and a time variable.

1.2 Concentration normalisation:
for each time t from 2016-01-01 to 2020-12-31:
  a. Randomly select a row in the original regressor matrix and store the value of the meteorological and seasonality variables in this row;
  b. Predict concentration with the base model using the value of t and values in *step a*;
  c. Repeat *steps a and b* for 400 times;
  d. Take an average across the predicts as the normalised concentration, $Y_t^{s,p}$.

Step 2: Change Point Detection

2.1 Detect structure changes:
- Detect any structure changes on $\{Y_t^{s,p}: t \in$ [2019-01-01, 2020-12-31)$\}$
- Store the detected changes as $\{c_j^{s,p}: j = 1,2,3, \dots n\}$

Step 3: Response Identification

3.1 Check for response to the lockdown:
  a. Create an empty set $J_{s,p}$;
  b. **for** each change point $c_j^{s,p}$ in $\{c_j^{s,p}: j = 1,2,3, \dots n\}$
    if $c_j^{s,p}$ lies within the margin period ($T_0 \pm 3$ weeks):
      add $c_j^{s,p}$ to set $J_{s,p}$ (Response identified);
  c. if $|J_{s,p}| = 0$:
    Add s to set S_p^0; set the effect estimate as zero: $\hat{\tau}^{s,p} = 0$;
    Return $\hat{\tau}^{s,p}$ (End).

Step 4: Research Period Specification

4.1 Initialise:
Set the start and end of the research period, $c_{\text{lb}}^{s,p}$ and $c_{\text{ub}}^{s,p}$, as the last change point in the pre-intervention period and the first change point in the post-intervention period that is outside the MP, respectively, where $c_{\text{lb}}^{s,p}, c_{\text{ub}}^{s,p} \in \{c_j^{s,p}\}$, lb and ub are the lower bound index and upper bound index.

4.2 Check for data quality:
- Check the start point:
  a. Set $\text{lb} = \text{lb} - 1$ until there are ≥25 valid daily average normalised concentrations within the period $[c_{\text{lb}}^{s,p}, T_0 - d)$ where $d = 3$ weeks as the length parameter of the donut hole;
  b. Store $c_{\text{lb}}^{s,p}$.
- Check the end point:
  a. Set $\text{ub} = \text{ub} + 1$ until there are ≥ 25 valid daily average normalised concentrations within the period $[T_0 + d, c_{\text{ub}}^{s,p})$, where $d = 3$ weeks as the length parameter of the donut hole;
  b. Store $c_{\text{ub}}^{s,p}$.

4.3 Exclude data within the donut hole:
Exclude the data within the donut hole ($T_0 \pm 3$ weeks) from the normalised concentrations within the period $[c_{lb}^{s,p}, c_{\text{ub}}^{s,p})$.

Step 5: Sharp RDD model

- Using the remaining data in *step 4.3* to estimate a sharp RDD model.
- Derive $\hat{\tau}^{s,p}$ with the estimated coefficients of the RDD model.
- **Return** $\hat{\tau}^{s,p}$ (End).

Figure S2-2. Pseudo code of the methodology for air quality effect estimation.

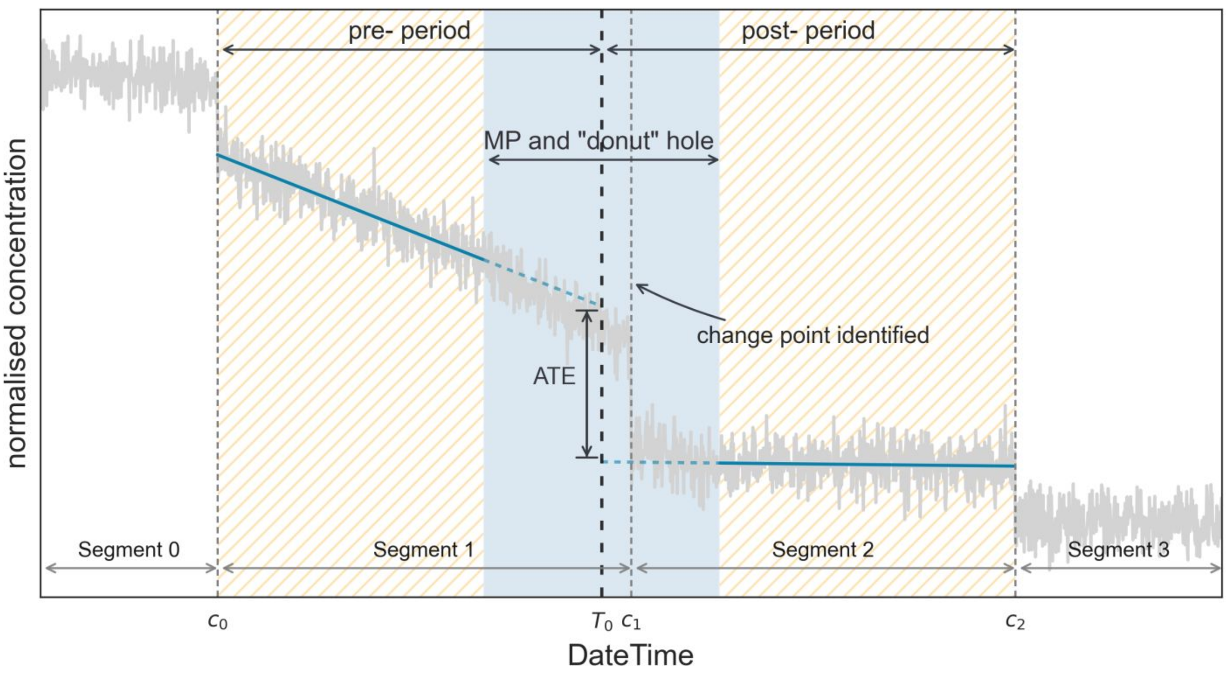

Figure S2-3. Graphical summary of the methodology for air quality effect estimation. The normalised air pollutant concentration time series (grey line) is illustrated with the detected change points c_j (grey; dashed lines). The margin period (MP) and the "donut hole" are shaded around the start of the lockdown T_0 (threshold) (black; dashed line). The MP and the donut hole are of the same length. The data within the orange hatched area are used for RDD model fitting. The average treatment effect (ATE) is given by the difference in intercept at T_0. The intercepts are estimated based on the trend function approximation (blue line) on either side of T_0.

S3. Meteorological normalisation

Meteorological normalisation is used in this paper to remove the variation in the observed air pollutant concentrations that can be explained by weather conditions and seasonality effects. The meteorological normalisation method of this paper follows Ma et al.[23,24] (baseline approach), where more methodological details are discussed. Compared with Ma et al.[23,24], we additionally conduct an uncertainty analysis for the meteorological normalisation process using a block bootstrapping approach and compare the air quality effects estimated based on the bootstrapping replicates with those based on the baseline approach. The introduction of the baseline approach and uncertainty analysis follows.

S3.1 Baseline approach

Base model

Specifically, a non-parametric model, gradient boosting decision trees (GBDT), is specified as the base model to describe the relationship between air pollutant concentrations and selected explanatory variables. The explanatory variables in the GBDT contain meteorological variables and seasonality variables. Meteorological variables include temperature, wind speed, wind direction, atmospheric pressure, relative humidity, rainfall, and Monin-Obukhov length. Seasonality variables are used to approximate the periodical pattern of air pollutant emissions, as highlighted by Hausman and Rapson[25]. The hour of the day (numeric), day of the week (categorical), and day of the year (numeric) are included. A time variable, using Unix time, is additionally considered to represent the long-term trend of concentrations.

GBDT model training

For each GBDT model, four key hyperparameters (*max_depth*, *subsample*, *learning rate*, and *colsample_bytree*) are tuned with Bayesian optimisation to improve model performance. The generalisation performance of the GBDT model is used as the objective function of the Bayesian optimisation; specifically, the model performance is evaluated with an *hv*-blocked cross-validation (CV) modified to incorporate additional considerations when using time series as input data, with the rooted mean squared error (RMSE) as the CV metric. The hyperparameter set that gives the smallest CV RMSE within 100 iterations is used to fit the final GBDT model. The GBDT model training and predicting are implemented with ***lightGBM*** library[26] in python, and hyperparameter tuning with ***scikit-learn*** library[27].

The number of boosted trees, *n_estimator*, is another important hyperparameter in a GBDT model, which controls the size of the ensemble model. Instead of tuning it like other key hyperparameters above, the *n_estimator* parameter of our GBDT model is determined by the early stop function of the ***lightGBM*** library; specifically, we specify the parameter *early_stopping_rounds* = 300 trees, which requires the model to stop training (adding trees) if the CV scores have not improved for 300 trees.

Figure S3-1 summarises the generalisation performance, as indicated by CV, of the final GBDT models using the hyperparameter set selected by Bayesian optimisation. To make a comparison among different air pollutants, the generalisation performance is illustrated by the coefficient of determination (R^2) ‡. The CV R^2 of a GBDT model provides an estimate of the model's ability to predict the output variable (i.e. air pollutant concentrations) on new data. The results in Figure S3-1 show that concentrations of NO_2, NO_x, and O_3 are generally more predictable by meteorological conditions and seasonality patterns compared to PM_{10} and $PM_{2.5}$. It is noted that some final GBDT models for concentrations of NO_2 or NO_x indicate a negative CV R^2. These cases are commonly found at roadside sites in or near central London, and we generally observed a dramatic decrease in air pollution at these places after the lockdown. As these GBDT models are intrinsically air pollution prediction models, they are very likely to have low generalisation performance when air pollution levels are dramatically influenced by factors other than the model's explanatory variables (meteorological and seasonality variables), such as the COVID-19 lockdown in this case.

Furthermore, to illustrate the effect of hyperparameter tuning, the generalisation performance of each final GBDT model is respectively compared with a GBDT model using the default hyperparameters in the ***lightGBM*** library. As indicated in Figure S3-2, the generalisation performance of the GBDT models is improved for all air pollutants, especially for PM_{10} and $PM_{2.5}$ concentrations. In addition, Figure S3-3 illustrates the goodness of fit of the final GBDT model for individual air pollutant concentration time series, where the model is fitted with the complete dataset and R^2 is evaluated on the same dataset for a particular time series. The R^2 shown in Figure S3-3 for the models of NO_x and NO_2 is generally comparable with the results in Grange and Carslaw[30].

‡ In this supporting information, R^2 of a model is defined as $R^2 = 1 - \frac{\sum_{i=1}^{n}(y_i - \hat{y}_i)^2}{\sum_{i=1}^{n}(y_i - \bar{y})^2}$, where y_i and $\hat{y}_i$ are the true value and the predicted value of the dependent variable of unit i, respectively; $\bar{y}$ is the average of y_i across units [66]. The adjusted R^2 of a model is defined as $\bar{R}^2 = 1 - \frac{\sum_{i=1}^{n}(y_i - \hat{y}_i)^2/(n-k-1)}{\sum_{i=1}^{n}(y_i - \bar{y})^2/(n-1)}$, where n is the number of observations in the model input and k is the number of independent variables (excluding the constant) in the model [66].

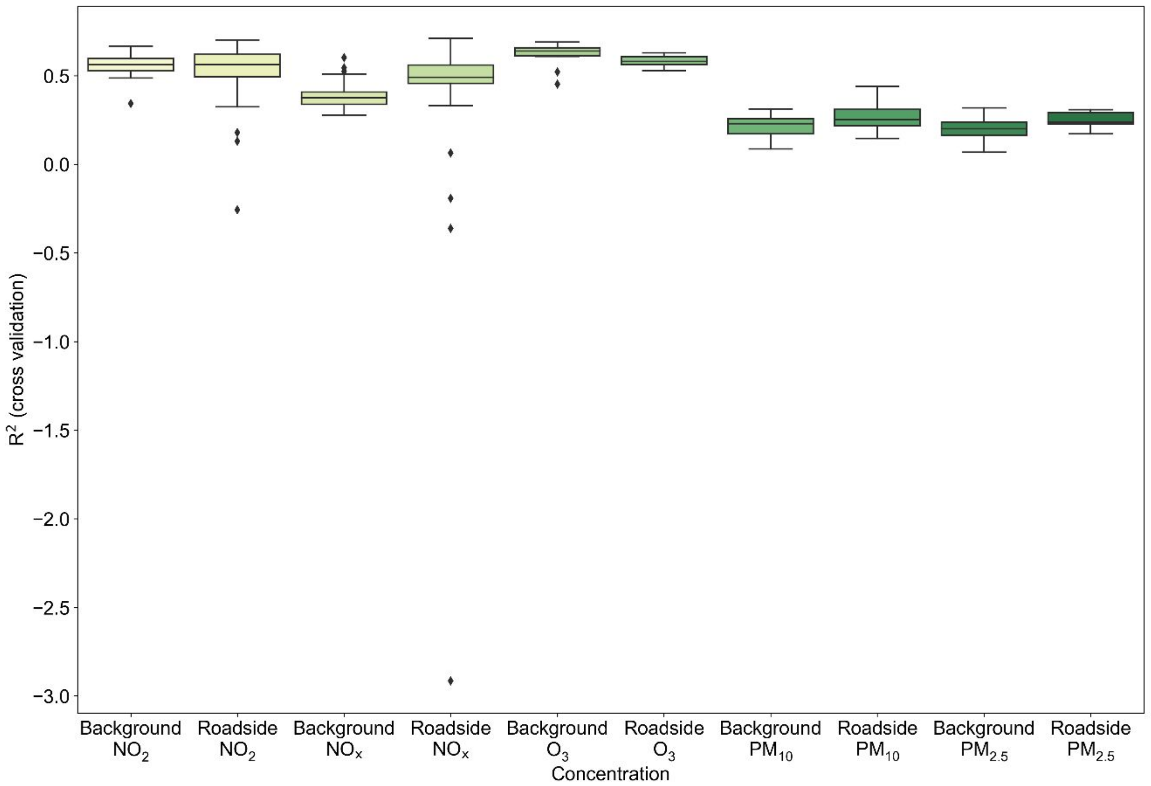

Figure S3-1. Generalisation performance of the final GBDT model built on different air pollutant concentrations. The generalisation performance of the GBDT model that uses the final hyperparameter set is evaluated with a modified *hv*-blocked CV for individual air pollutant concentration time series. A GBDT model is repeatedly fitted with the final hyperparameter set on different training sets in CV and then evaluated on the corresponding test set with R^2. The mean of the test set R^2 is used to determine the CV R^2 of the final GBDT model (y-axis) for a particular air pollutant concentration time series.

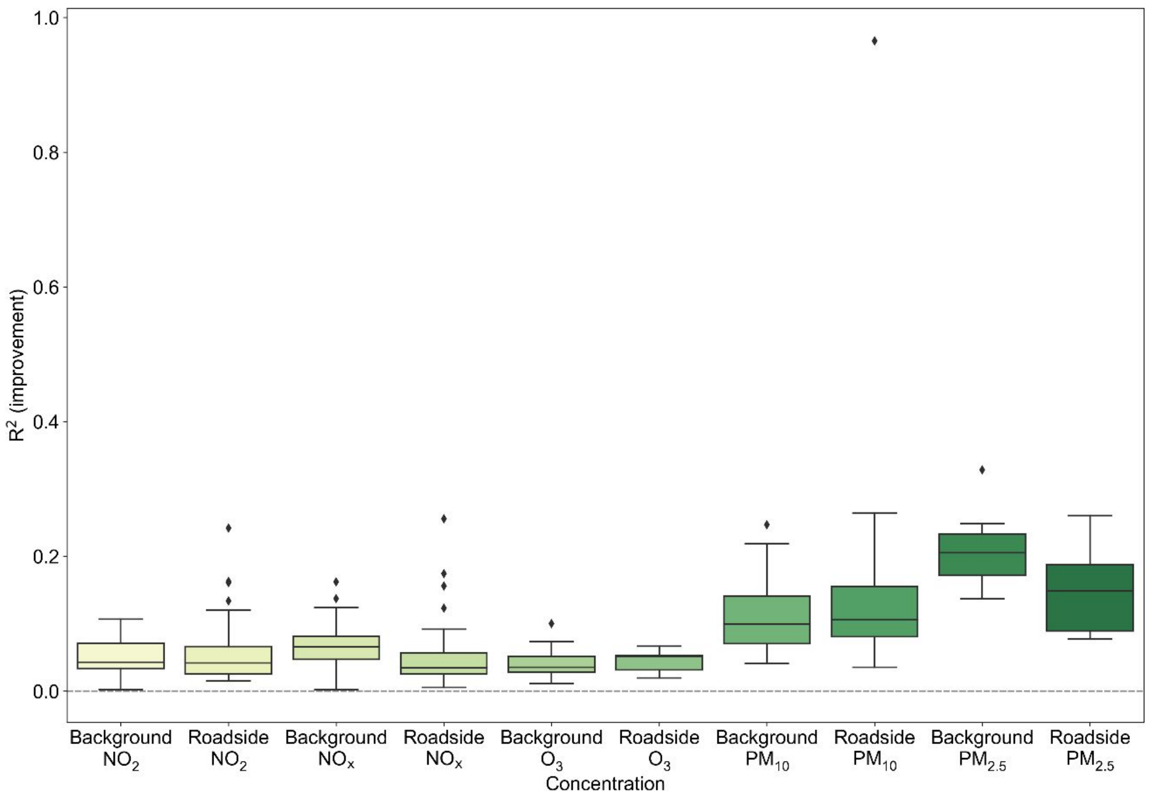

Figure S3-2. Improvement in generalisation performance with hyperparameter tuning for different air pollutant concentrations. The generalisation performance of the GBDT model that uses the optimal hyperparameter set from hyperparameter tuning ('R^2_{tuned}') is compared with that of the model using the default hyperparameters of the lightGBM library in python ('$R^2_{default}$'). The generalisation performance of a particular GBDT model is evaluated with CV R^2. Improvement is calculated as R^2_{tuned} - $R^2_{default}$.

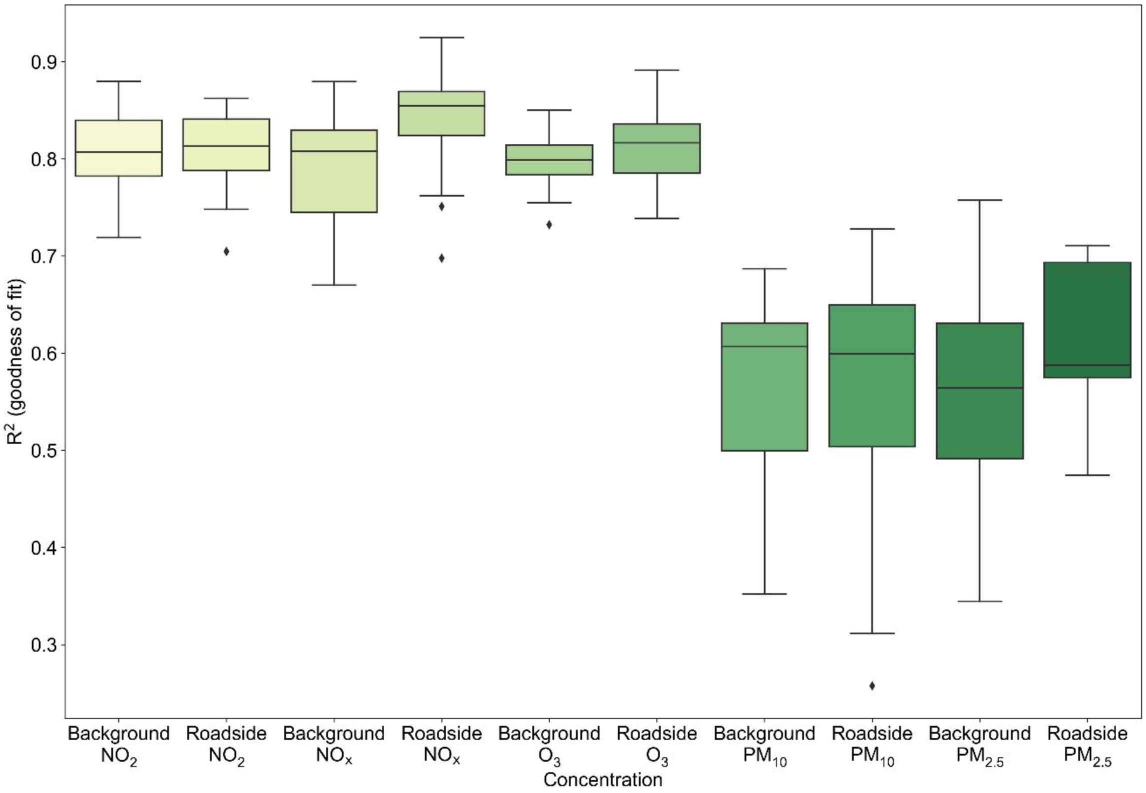

Figure S3-3. Goodness of fit of the final GBDT model built on different air pollutant concentrations. The goodness of fit of the GBDT model using the final hyperparameter set is evaluated with R^2 for individual air pollutant concentration time series. The model is fitted with the complete dataset and R^2 is evaluated on the same dataset to determine the goodness of fit of that model.

Concentration normalisation

After fitting the GBDT model, a normalised concentration at time t is derived by the mean of the concentration predicts; each predict is derived by randomly sampling the original regressor matrix and predicting with the fitted GBDT model:

$$Y_t^{s,p} = \frac{1}{Z}\sum_{i=1}^{Z} \hat{f}_{s,p}(t, \boldsymbol{V}_{\setminus T}^{(i)}) \quad \text{Eq. S3-1}$$

where $Y_t^{s,p}$ is the normalised concentration of air pollutant p at site s at time t; $\hat{f}_{s,p}(.)$ is the fitted GBDT model for air pollutant p at site s; T is the time variable, with t being its value represented by Unix time; $\boldsymbol{V}$ is the original regressor matrix, containing the values of all explanatory variables; $\boldsymbol{V}_{\setminus T}^{(i)}$ is the i-th sample of $\boldsymbol{V}$ with all explanatory variables excluding T; Z is the total number of predictions.

The normalised air pollutant concentration time series is the partial dependence of the time variable T if the sampling and predicting procedure for each time t goes through every sample in $\boldsymbol{V}$ without replacement[23]. The partial dependence of T shows the marginal effect of T on predicting the response variable (air pollutant concentration) by the fitted model[28]. However, considering the large sample size, estimating the partial dependence is computationally expensive and, in this case, we specify Z as a large enough value, here $Z = 400$. In other words, the normalised pollutant concentration used in this paper is the mean of 400 predicted concentrations with random meteorological and seasonality conditions.

An example of air pollutant concentration time series before and after the meteorological normalisation is shown in Figure S3-4.

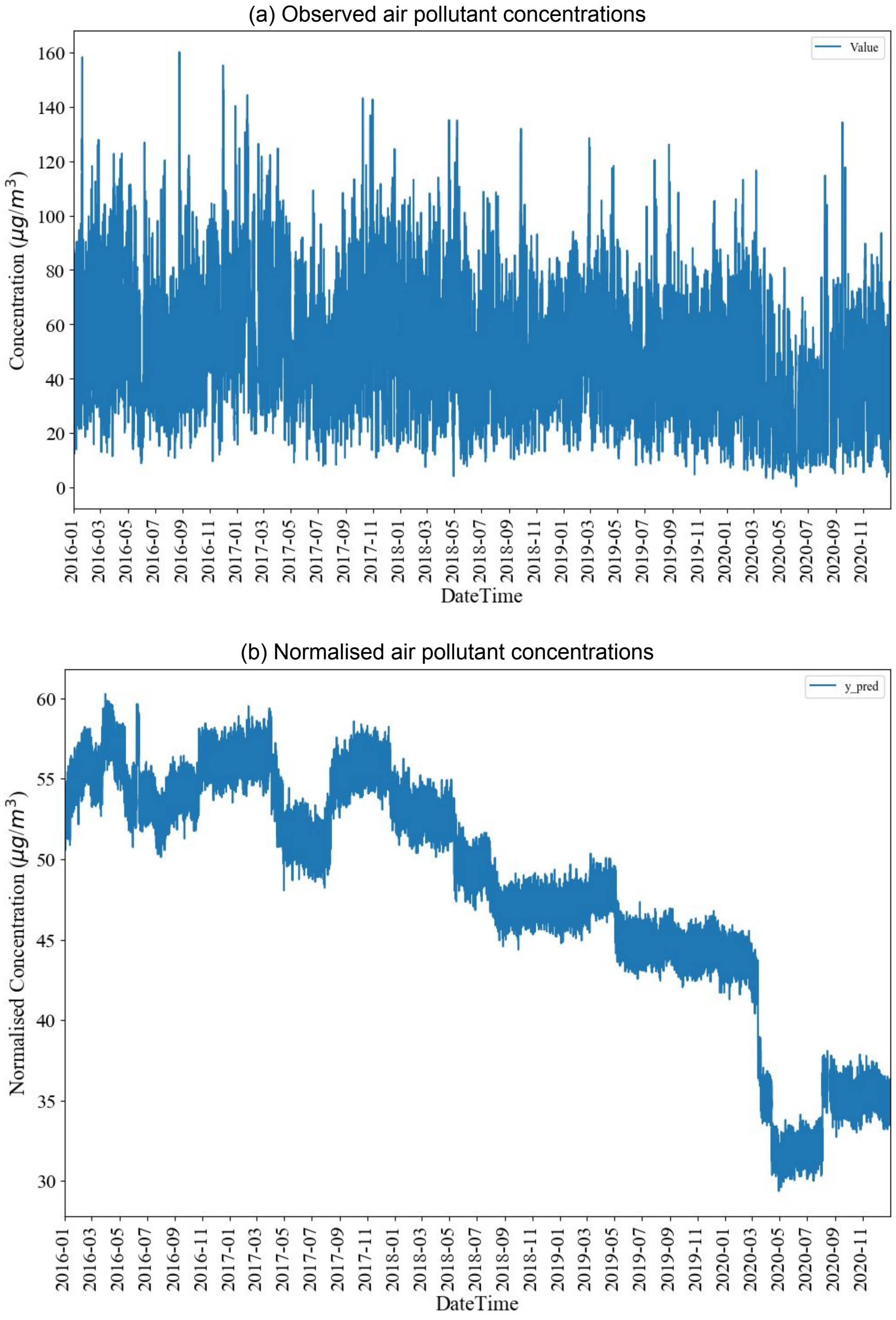

Figure S3-4. Example of air pollutant concentration time series (a) before and (b) after the meteorological normalisation: NO_2 concentrations at roadside site HK6 in the London Air Quality Network.

S3.2 Uncertainty analysis

In this section, the robustness of air quality effect estimates on the meteorological normalisation process is tested using a block bootstrapping approach. Considering the computational cost, we randomly select 25 air pollutant concentration time series analysed in our study (10% of the total cases) for this uncertainty analysis, which are called "test cases" hereafter. Bootstrapping is a popular approach to estimating the uncertainty of a model or a parameter of interest. It is typically done by randomly resampling the original dataset with replacement to construct new samples (replicates) and repeatedly fitting the model (or computing the parameter of interest) with these replicates. The resulting distribution of the estimates of interest can then be used to compute uncertainty measures, such as confidence intervals (CIs)[29].

To test the uncertainty due to meteorological normalisation, we train 50 GBDT models for each test case and use them individually as the base model for normalisation (c.f. §S3.1); in particular, each GBDT model uses the same hyperparameter set as the corresponding baseline model (c.f. §S3.1) but is trained with a randomly sampled input dataset (bootstrapping replicate). We set the number of bootstrapping replicates as 50 to consider the computational cost and follow Grange and Carslaw[30], who used 50 random forest models from a standard bootstrapping process to estimate the CI of the normalised concentration time series for NO_2 at a particular monitoring site in London. However, an underlying assumption of the standard bootstrapping process is that all the observations in the original dataset are independently identically distributed[29], making it inappropriate for time series data. In this case, a more sophisticated approach, such as block bootstrapping, is necessary for a dependent dataset. Ultimately, block bootstrapping first splits the data into different blocks based on its dependency nature and then resamples the data based on blocks instead of individual data points. Examples of applications of block bootstrapping in environmental research include De Foy et al.[31], Peng et al.[32], and Wen et al.[33]. In our study, we use block bootstrapping with block size = 24 hours to resample the original input dataset of the base model for each test case; particularly, we group the original hourly input dataset into daily blocks, and then randomly resample these blocks (of the same size as the original dataset) with replacement for each bootstrapping replicate r, $\forall r \in [1, 50]$. It should be noted that we use the same random seed for each test case to eliminate variation caused by different block choices across different test cases.

We denote the set of fitted GBDT models for pollutant p at site s as $\{\hat{f}^r_{s,p}(\cdot){:}r = 1,2, \ldots,50\}$, where $\hat{f}^r_{s,p}(\cdot)$ is the model trained on replicate r. These models are then individually used as the base model for normalisation (c.f. Eq. S3-1); specifically, 400 predicted concentrations are generated using $\hat{f}^r_{s,p}(\cdot), \forall r \in [1, 50]$, with random meteorological and seasonality conditions for each time t and then averaged for the normalised concentration at that time. Note, that the random meteorological and seasonality condiations are drawn from the original hourly input rather than the bootstrapping replicate. This ensures that we preserve the maximum amount of information from the original dataset while testing the uncertainty in fitting the predictive GBDT model. We denote the normalised concentration time series associated with $\hat{f}^r_{s,p}(\cdot)$

as $\boldsymbol{Y}^{s,\,p,r}$. The mean and 95% percentile interval across the 50 time series are then estimated for each time t and used to construct the central estimate and 95% CI of the normalised concentration time series under the bootstrapping approach, respectively.

An example of comparing normalised concentration time series obtained from the baseline approach and bootstrapping approach is illustrated in Figure S3-5. Together with the results for other test cases, we find that the normalised concentration time series derived from the baseline approach generally follows the same trend as the central estimate from the bootstrapping approach. Additionally, most of the normalised concentrations from the baseline approach are within the 95% CI estimated in the bootstrapping approach, indicating a strong agreement between these two approaches.

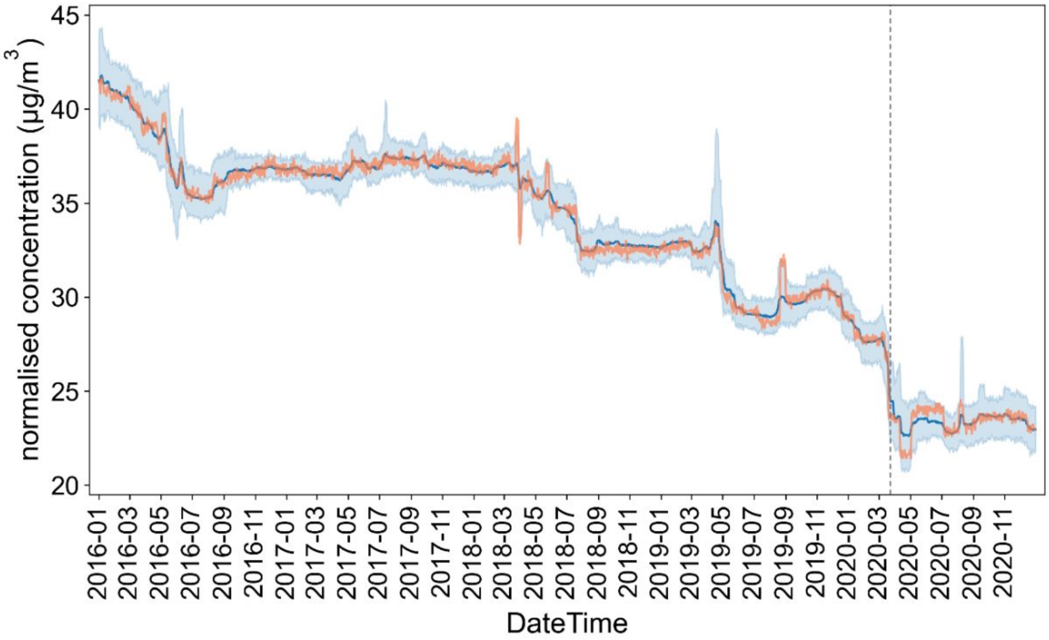

Figure S3-5. Comparison of normalised concentration time series for NO_2 in 2016-2020 at roadside site GR9 (LAQN) using the baseline (orange) and bootstrapping (blue) approaches. The normalised concentration at a given time is estimated by averaging predicted concentrations from random meteorological and seasonality conditions. Predictions in the baseline approach are derived with a single GBDT model trained on the entire dataset, while the bootstrapping approach generates 50 GBDT models using the same hyperparameters as the baseline model but trained on different bootstrapping replicates of the original dataset. The blue central line and shaded area show the mean and 95% CI estimated across the 50 normalised concentration time series generated by these GBDT models.

To further understand the impact of meteorological normalisation on effect estimates, we investigate two methods to quantify uncertainty and compare their results with the baseline effect estimates used in the manuscript. In the first method, we bootstrap the entire air quality effect evaluation process based on

$\{\boldsymbol{Y}^{s,p,r}{:}r = 1,2,\ldots,50\}$. Specifically, we repeatedly perform a change point detection and subsequently fit an RDD model (if the response to the lockdown is identified) to generate an effect estimate $\hat{\tau}^{s,p,r}$ for individual $\boldsymbol{Y}^{s,p,r}$, $\forall r \in [1, 50]$. We reserve $\{\hat{\tau}^{s,p,r}{:}r = 1,2,\ldots,50\}$ for each test case. In the second method, we solely bootstrap the meteorological normalisation step. Particularly, we aggregate the set of normalised concentration time series, $\{\boldsymbol{Y}^{s,p,r}{:}r = 1,2,\ldots,50\}$, in advance and use the central estimate (mean) of these time series as the input to the subsequent change point detection and RDD model. We denote the effect estimate derived from the second method as $\hat{\tau}^{s,p,r^*}$. Note, that we generate the interval estimates of $\hat{\tau}^{s,p,*}$ using a Monte Carlo simulation in Ma et al.[23]; this method is also used to derive the interval estimates of effects in the baseline approach. Finally, we compare $\{\hat{\tau}^{s,p,r}{:}r = 1,2,\ldots,50\}$ and $\hat{\tau}^{s,p,r^*}$ with the baseline estimate $\hat{\tau}^{s,p}$ for each test case.

An example of comparing baseline effect estimates with those from the uncertainty analysis is shown in Figure S3-6. The 95% CI of the baseline effect estimate for NO_2 at site GR9 overlaps with the 95% CI of the effect estimate when we solely bootstrap the meteorological normalisation step. Meanwhile, the baseline estimate also falls within the range of the 50 effect estimates in the first method, where the entire evaluation process is bootstrapped, and is close to the median of these estimates. These findings suggest that our baseline result for this test case is robust to the meteorological normalisation process.

To quantitatively compare $\hat{\tau}^{s,p}$ with $\{\hat{\tau}^{s,p,r}{:}r = 1,2,\ldots,50\}$ for individual test cases, we use the interquartile range (IQR) method to determine whether $\hat{\tau}^{s,p}$ is a potential outlier for $\{\hat{\tau}^{s,p,r}{:}r = 1,2,\ldots,50\}$. The IQR method is commonly used in statistics to identify extreme values for univariate data[34]. In our study, we consider the baseline estimate non-robust to the first uncertainty scenario if the 95% CI of $\hat{\tau}^{s,p}$ falls outside the range of

$$\left[\hat{\tau}^{s,p}_{0.25} - 1.5 * \mathrm{IQR}^{s,p}, \hat{\tau}^{s,p}_{0.75} + 1.5 * \hat{\tau}^{s,p}_{0.75}\right],$$

where $\hat{\tau}^{s,p}_{0.25}$ and $\hat{\tau}^{s,p}_{0.75}$are the first and third quartiles of $\{\hat{\tau}^{s,p,r}{:}r = 1,2,\ldots,50\}$, respectively; $\mathrm{IQR}^{s,p} = \hat{\tau}^{s,p}_{0.75} - \hat{\tau}^{s,p}_{0.25}$ is the corresponding interquartile range. Here, we use quartiles rather than the mean or 95% percentile interval because the number of bootstrapping replicates in our uncertainty analysis is relatively small, and the distribution of effect estimates in the first method can be skewed (c.f. the first panel in Figure S3-6). Our results suggest that the baseline effect estimate of all the test cases is robust to the meteorological normalisation when bootstrapping the entire evaluation process.

Furthermore, for each test case, we compare the central estimate of $\hat{\tau}^{s,p}$ with that of $\hat{\tau}^{s,p,r^*}$ f, i.e. the black dots in the second and third panels in Figure S3-6. Only 5 of the 25 test cases show a change in the magnitude of the effect estimate of more than 0.05. The results indicate that when only bootstrapping the meteorological normalisation step, the majority of the test cases (80%) are also robust to it.

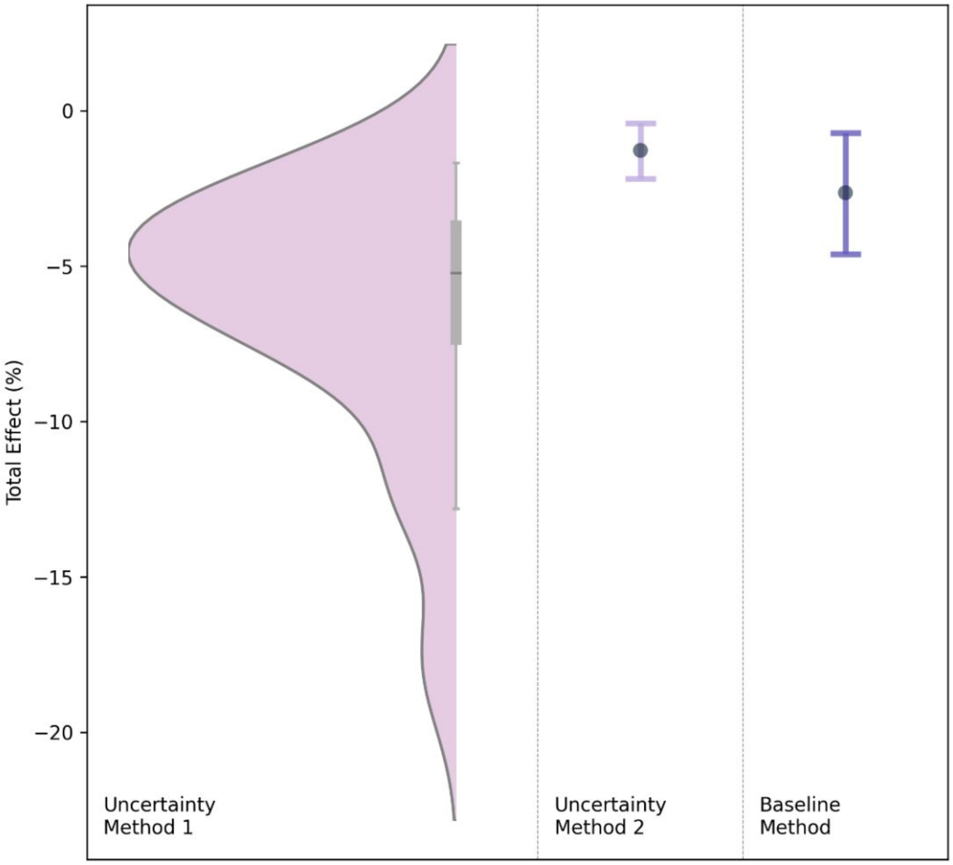

Figure S3-6. Comparing estimated total effects on NO_2 concentrations at roadside site GR9 using bootstrapping approaches and the baseline approach. The first panel illustrates the distribution of the estimated total effects using 50 individual normalised concentration time series obtained through bootstrapping; the pink shaded area shows the kernel density estimation plot; the box plot shows the quartiles of these effect estimates, with whiskers extending to the value within 1.5 times of the interquartile range. The second panel illustrates the estimated total effect obtained by averaging the 50 individual normalised concentration time series in advance; the central estimate (black dot) and 95% CI (uncertainty bars) of the total effect are derived by a Monte Carlo simulation on relevant model coefficients. The third panel shows the baseline total effect estimate; the central estimate (black dot) and the 95% CI (uncertainty bars) of the total effect are derived by a Monte Carlo simulation on relevant model coefficients.

S4. Change point detection

Various change point detection (CPD) algorithms can be used to identify a variety of change points, including changes in the properties of a signal (for example, mean-shift) and changes in linear relationships between variables (structural change)[35]. In this paper, we specify a CPD algorithm to detect structural changes in the trend of normalised air pollutant concentrations, mainly following Ma et al.[24] with some adjustments. A change in the slope of the linear trend and/or an abrupt discontinuity in the normalised concentration time series is identified as a structural change.

S4.1 Model specification

The normalised air pollutant concentration time series is assumed to follow a piecewise linear regression model with n breaks located at $\{c_j^{s,p}: j = 1,2,3,\ldots n\}$:

$$Y_t^{s,p} = \delta_j + \theta_j K(t) + \varepsilon_t, \ \forall t \in [c_j^{s,p}, c_{j+1}^{s,p}), \qquad \text{Eq. S4-1}$$

for $j = 0,1,2,\ldots, n$, where $Y_t^{s,p}$ is the normalised concentration of air pollutant p at site s at time t; $K(t) = t$ is the linear trend function and the value of time t is represented by Unix time; $c_j^{s,p}$ is the location of the j-th change point detected on $Y_t^{s,p}$ and we conventionally define $c_0^{s,p}$and $c_{n+1}^{s,p}$ as the start and the end position of $\{Y_t^{s,p}\}$; δ_j and θ_j are respectively the coefficient of the constant and the time variable in regime $[c_j^{s,p}, c_{j+1}^{s,p})$, and can vary over regimes; ε_t is the error term. The structural change points $\{c_j^{s,p}: j = 1,2,3,\ldots n\}$ are treated as unknown. The aim is to estimate the unknown model coefficients and the unknown change points. The change point estimators are the global minimisers of the sum of the minimal sum of squared residuals in each regime. The model coefficients in each regime are the least-squares estimates. The CPD model specified in Eq. S4-1 can help to locate the change in the slope of the linear trend and/or an abrupt discontinuity in the normalised pollutant concentration time series.

S4.2 Algorithm specification

The hourly normalised concentrations are aggregated to daily averages to conduct the CPD. The CPD is implemented in python with the ***ruptures*** library[35] to apply a dynamic programming algorithm proposed by Bai and Perron[36]. Within the process, the number of breaks is predetermined, and any change points indicating a time series segment of less than 30 days are skipped. A series of CPD processes is conducted by individually setting the number of breaks n, $\forall n \in [1, 20]$. The parameter n is then determined based on the model performance evaluated using the Bayesian Information Criterion (BIC). Specifically, the best candidate model (including the optimal number of breaks) is selected with the *DiffBIC* method proposed by Zhao *et al* [37], which detects the knee point of the resulting BIC curve.

The CPD algorithm specified above aims to identify the most significant change points in the input signal by a certain amount. Consequently, when conducting a CPD on the entire time series, a change in concentrations that is relatively more subtle than the large changes achieved in previous years may be

ignored; for example, the changes in concentrations due to the recovery after the ease of lockdown may be undetectable in this case, as the initial growth in activities in several sectors was slow and gradual[1]. Therefore, to better support the research period specification for the case study of lockdown, the CPD in this paper only focuses on a more recent period; specifically, the CPD is conducted on a more recent subset of normalised concentration time series, from 2019-01-01 to 2020-12-31, rather than the entire dataset (from 2016-01-01 to 2020-12-31) like in Ma et al.[24].

Figure S4-1 shows an example of the CPD results on the normalised pollutant concentration time series.

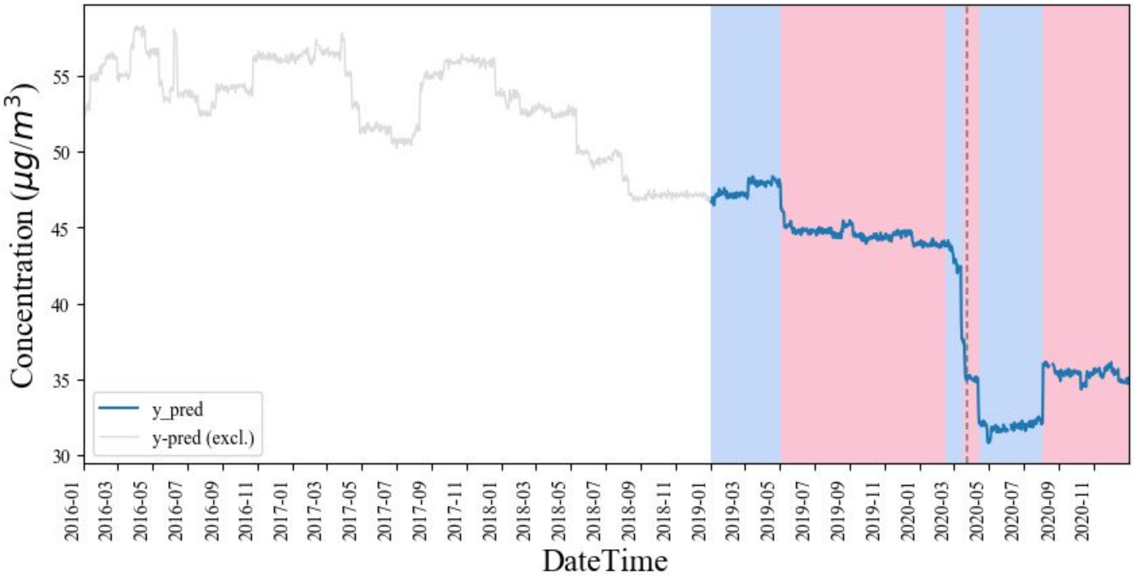

Figure S4-1. Change point detection on the normalised daily average NO_2 concentrations time series at roadside site HK6 in the London Air Quality Network. Change points are detected on a subset of the normalised concentration time series (from 2019-01-01 to 2020-12-31; blue line) and the rest of the normalised concentrations is indicated with a grey line. The start of COVID-19 lockdown is indicated with the grey vertical dashed line.

S5. Sharp RDD model and derivaion of effect estimate

This section details the connection between the steps of change point detection (CPD) and the sharp RDD model in our research framework (c.f. SI §S2). The specification of the sharp RDD model and the derivation of the total effect estimate are also introduced in this section. The methodological details introduced in this section mainly follow Ma et al.[24].

S5.1 Response identification

To justify the use of sharp RDD model, it is necessary to check the discontinuity of the outcome variable at the threshold of the forcing variable[38]. We perform this check based on the results of CPD. Instead of strictly checking at the start of the lockdown (threshold), we introduce a "margin period" (MP) around the threshold to consider the potential uncertainties in previous steps (c.f. SI §S2). A normalised concentration time series is considered to have responded to the lockdown if it has detected change point(s) that lie within the MP. A sharp RDD model is then specified where the monitoring site showed a response. By analysing the timing of the response across monitoring sites (see details in SI §S7), the MP is determined to be 3 weeks before and after the lockdown, i.e. from 2020-03-02 to 2020-04-13.

S5.2 Research period specification

The detected change points are also used to support the research period specification of sharp RDD models. Specifically, to mitigate influences from potential unobservable confounders and unrelated interventions, the normalised concentration time series is truncated into segments based on the detected change points; only the data in the segments that are near the start of the lockdown T_0 are used to estimate the RDD model. The start and end of the research period for the sharp RDD model are respectively specified as the last change point in the pre-intervention period and the first change point in the post-intervention period that is outside the MP and satisfies the data quality requirement of the time series segment. As the lockdown measures started to ease gradually from 2020-05-10 (~7 weeks after lockdown), instead of requiring the pre- and post- intervention segment to be at least 8 weeks like in Ma et al.[24], we ease the data quality requirement; in this paper, both the pre- and post- intervention segments are required to have at least 25 valid daily average normalised concentrations.

Within the research period defined by the detected change points, we specify the sharp RDD model as a *donut RDD* instead of a *regular RDD* to account for any anticipation, adaptation, or delay in response to the lockdown, following Ma et al.[24]. Unlike a regular RDD that estimates the effect with all the data in the research period, a donut RDD excludes the data in the vicinity of the threshold (called the "donut hole")[24]. Therefore, we estimate the trends of the normalised concentrations in Eq. S5-1 by two relatively stable equilibriums, consequently giving a better estimation of the full lockdown effect. Note, that effect estimates under the donut RDD setting would be similar to those from the regular RDD if a transition in effect does not exist or is not obvious[24]. For simplicity, we set the donut hole as a symmetric period and of the same

length as the margin period. In other words, all the data within 3 weeks before and after the lockdown are excluded from the RDD model estimation.

The length of pre- and post- intervention periods specified in the case study are summarised in SI §S6.

S5.3 Model specification

The sharp RDD model specification of this paper follows Ma et al.[23,24]. Normalised hourly concentrations are used to calculate 24-hour averages to reduce noise in time series. The model is based on a sharp RDD in time with the start of the lockdown, T_0, being the threshold:

$$y_t^{s,p} = \delta_0 + \delta_1 W_t + \theta_1 K(t) + \theta_2 K_{\text{post}}(t) + \sum_{l=1}^{L} \alpha_l y_{t-l}^{s,p} + \varepsilon_t \qquad \text{Eq. S5-1}$$

where $y_t^{s,p}$ is the natural logarithm transformation of the average normalised concentration of pollutant p at site s at day t; $W_t = \begin{cases} 1, & t \geq T_0 \\ 0, & t < T_0 \end{cases}$ is the treatment indicator; $K(t) = t$ is the long-term trend function, and $K_{\text{post}}(t) = \begin{cases} t - T_0 + 1, & t \geq T_0 \\ 0, & t < T_0 \end{cases}$ affects the post-intervention trend; both $K(t)$ and $K_{\text{post}}(t)$ are assumed to be linear to be consistent with the CPD model specification (c.f. SI §S4); the value of time t and T_0 is represented by the Unix time if included as variables; $y_{t-l}^{s,p}$ is the lagged dependent variable where l is the order of lag and L is the maximum lag order to be included; ε_t is the error term. While θ_1 controls the long-term trend of $y_t^{s,p}$, θ_2 indicates the change in the trend after the intervention. The coefficient α_l represents the correlation in concentrations on the current day and l days before. The coefficients $\boldsymbol{\alpha}$ represent the autocorrelation features of the daily average normalised concentration time series.

The model is estimated by ordinary least squares with Newey-West standard errors. The main coefficient of interest is δ_1. It can be interpreted as the average treatment effect (ATE) where $t = T_0$, and can be generalised as the population ATE (if assume the treatment effect is constant over time), or the weighted ATE across time (if assume the treatment effect is variable)[23,39]. As the autocorrelation features of the outcome variable are considered in the model, the coefficient δ_1 can be treated as an impact multiplier. The causal air quality impact of lockdown, τ, is then derived with δ_1 and $\boldsymbol{\alpha}$ to calculate the sum of the impact from the current daily period and the stacked impact from the previous (lagged) daily periods[40]. The derivation of the total effect follows.

S5.4 Derivation of air quality effect

Assume an autoregression model of order *L*, AR(*L*), with a binary intervention:

$$y_t = \delta_0 + \delta_1 W_t + \alpha_1 y_{t-1} + \alpha_2 y_{t-2} + \dots + \alpha_L y_{t-L} \qquad \text{Eq. S5-2}$$

where $W_t = \begin{cases} 1, & t \geq T_0 \\ 0, & t < T_0 \end{cases}$ is the intervention indicator.

The corresponding stacking equations can be represented by:

$y_{t-1} = \delta_0 + \delta_1 W_{t-1} + \alpha_1 y_{t-2} + \alpha_2 y_{t-3} + \ldots + \alpha_{L-1} y_{t-L} + \ldots,$

$y_{t-2} = \delta_0 + \delta_1 W_{t-2} + \alpha_1 y_{t-3} + \alpha_2 y_{t-4} + \ldots + \alpha_{L-2} y_{t-L} + \ldots,$

…

By substituting the stacking equations into the main AR(*L*) model, it can be proved that,

the effect from the 1st time lag is $\alpha_1\delta_1$;

the effect from the 2nd time lag is $(\alpha_2 + {\alpha_1}^2)\delta_1$;

the effect from the 3rd time lag is $[\alpha_3 + (2\alpha_1\alpha_2 + {\alpha_1}^3)]\delta_1$;

…

Therefore, suppose the maximum lag order $L = 3$, the total effect of intervention is then

$\tau = \delta_1 + \alpha_1\delta_1 + (\alpha_2 + {\alpha_1}^2)\delta_1 + [\alpha_3 + (2\alpha_1\alpha_2 + {\alpha_1}^3)]\delta_1.$

S6. Research period of sharp RDD models

This section summaries the length of pre- and post- intervention periods specified in the case study. The start and end of the research period for each sharp RDD model are respectively specified as the last change point in the pre-intervention period and the first change point in the post-intervention period that is outside the margin period and satisfies the data quality requirement of the time series segment. Additionally, as we specify the main model as a donut RDD, all the data within 3 weeks before and after the lockdown (i.e. donut hole) are excluded. Methodological details are introduced in SI §S5.

Table S6-1. Summary of the length of research period specified in the sharp RDD model at roadside sites.

Pollutant	Site count	Pre-period (days) [a]				Post-period (days) [a]			
		mean	std	min	max	mean	std	min	max
NO_2	45	146	106	50	447	189	69	71	283
NO_x	42	107	67	50	327	220	69	71	283
O_3	3	179	117	110	314	173	97	98	283
PM_{10}	12	246	86	76	329	161	67	55	254
$PM_{2.5}$	0	-	-	-	-	-	-	-	-

(a) The mean value and standard deviation are rounded to the nearest integer.

Table S6-2. Summary of the length of research period specified in the sharp RDD model at background sites.

Pollutant	Site count	Pre-period (days) [a]				Post-period (days) [a]			
		mean	std	min	max	mean	std	min	max
NO_2	27	110	76	46	366	198	67	58	283
NO_x	23	92	33	49	175	254	39	168	283
O_3	2	241	65	195	287	170	160	57	283
PM_{10}	6	213	108	50	301	222	70	80	254
$PM_{2.5}$	0	-	-	-	-	-	-	-	-

(a) The mean value and standard deviation are rounded to the nearest integer.

S7. Timing of response

As discussed in SI §S5.1, we define a margin period, i.e. a short period around the start of the lockdown, to identify the monitoring sites that had a response to the lockdown. A monitoring site is considered to have responded to the lockdown if the normalised concentration time series has detected change point(s) that lie within the margin period.

The proportion of monitoring sites where at least one change point was detected within the margin period, referred to as the *response ratio* hereafter, is shown in Figure S7-1 for different sizes of the margin period. Detectable changes in air quality were found around the start of the lockdown at various locations. A sharp increase in response ratio generally occurred within 3 weeks on either side of the start of the lockdown, particularly for NO_2, NO_x, and the roadside concentrations of all air pollutants except for $PM_{2.5}$. These results are generally consistent with the real-world evidence that both transport demand and public transport provision in London was significantly reduced by the lockdown[1]; moreover, the most significant decrease in transport demand generally occurred within 2 weeks after the lockdown; by 65%, 97%, and 85% respectively for road traffic, London Underground, and bus demand compared to the levels in 2019[1].

Our results also show that the response ratio has generally reached a temporal plateau for a margin period of 3-5 weeks on either side of the start of the lockdown. Detectable change points were found at 95% and 91% of the monitoring sites for NO_2 and NO_x concentrations respectively if the length of the margin period is set as 5 weeks. The response ratio for the pollutants that are less affected by road transport activities (O_3, PM_{10}, and $PM_{2.5}$) are relatively low compared with NO_2 and NO_x; 59% for O_3, 37% for PM_{10}, and 14% for $PM_{2.5}$ within the 5-week margin period. The roadside concentrations generally had a quicker response and a higher response ratio compared with background concentrations. The results generally reveal the difference in activity changes among different sectors during the lockdown, where the mobility restrictions dramatically reduced London's road traffic, while activities in some other sectors in London were less affected (such as power generation) or even increased (such as residential activities)[1,41].

As the lockdown measures were eased from 2020-05-10 (~7 weeks after the lockdown), the length of the margin period is determined to be 3 weeks for the analysis in the manuscript, with the resulting margin period (and also the "donut hole") from 2020-03-02 to 2020-04-13; a longer margin period may intercept with the change point(s) that were related to the ease of lockdown.

Figure S7-2 further illustrates the response ratios for different air pollutants by distinguishing the periods before and after the lockdown. The results suggest that a large proportion of monitoring sites showed a response in a short period before the lockdown, indicating an anticipation effect. This justifies the need for using a donut RDD instead of a regular RDD (c.f. SI §S5.2) to have a better estimation of the full lockdown effect.

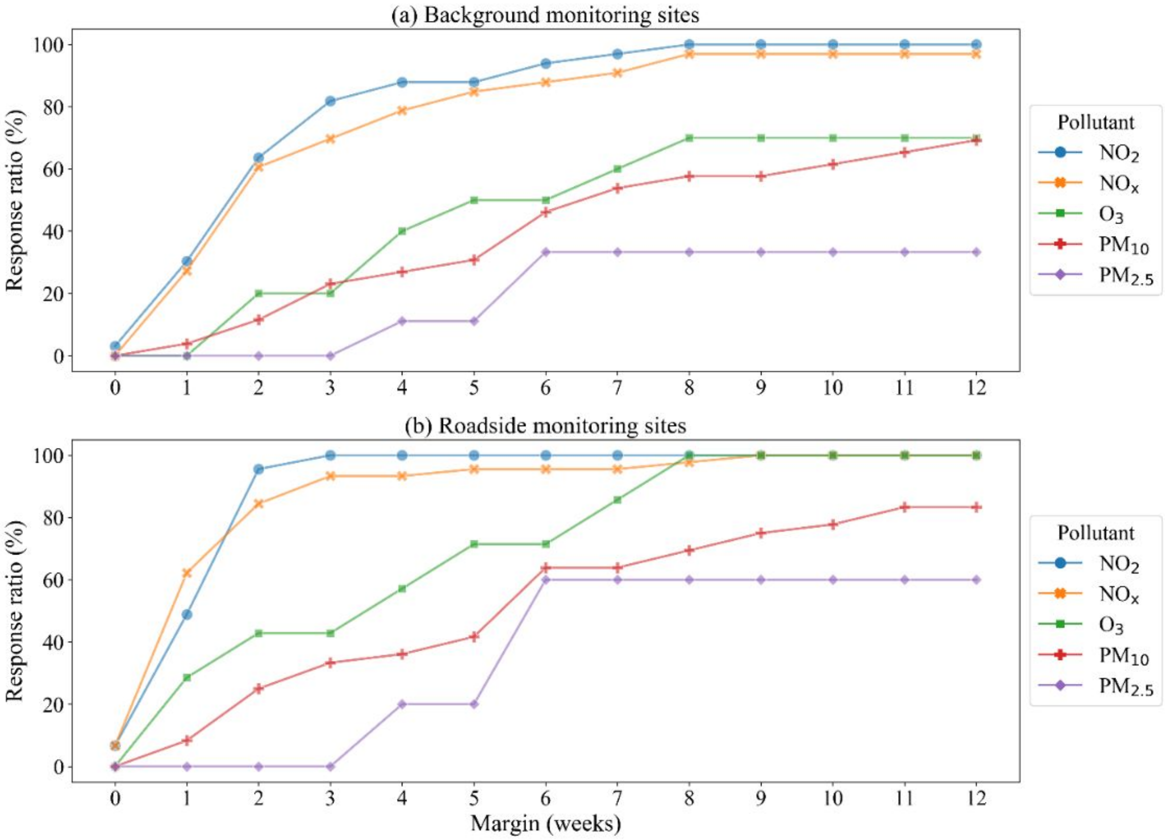

Figure S7-1. Monitoring site response ratio for different margin periods, which is a symmetric period around the start of the lockdown (threshold) whose length in weeks (on either side) is indicated on the x-axis. The response ratio (y-axis) is the proportion of monitoring sites where change point(s) were detected within the margin period.

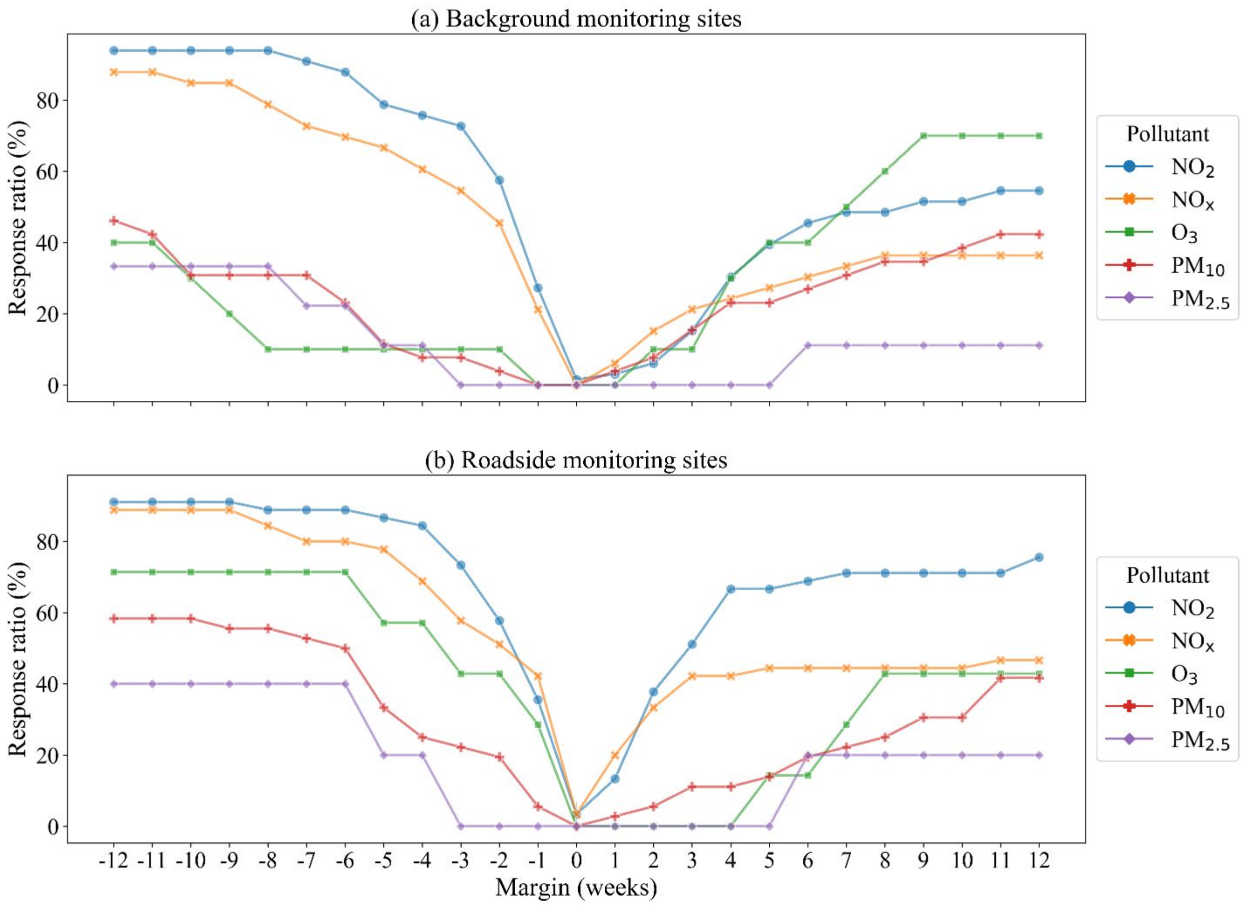

Figure S7-2. Monitoring site response ratio for different periods around the start of the lockdown, whose length in weeks (on one side) is indicated on the x-axis (negative: before the lockdown; positive: after the lockdown). The response ratio (y-axis) is the proportion of monitoring sites where change point(s) were detected within the period.

S8. Methods for mapping lockdown impacts

To estimate the lockdown impacts at individual MSOAs, a mapping method is applied to interpolate the lockdown impacts, in absolute and relative terms, at individual monitoring sites to the unified grids (spatial resolution: 1 km × 1 km), which are then averaged to the MSOA level[§]. The method mainly follows Horálek et al.[42,43], which has been used by the European Environment Agency to generate annual air quality maps and population exposure estimates. In general, it combines a linear regression model and ordinary kriging of the residuals produced from the linear regression ('residual kriging'); the introduction of individual components follows.

S8.1 Linear regression

To interpolate the target variable, a linear regression model is built on the target variable with several supplementary data (c.f. Table S1-2):

$$L_s = c + \sum_1^5 a_i x_{i,s} + \eta_s, \qquad \text{Eq. S8-1}$$

where L_s represents the target variable (lockdown impact in absolute or relative terms) at monitoring site s; $x_{1,s}$, $x_{2,s}$, $x_{3,s}$, and $x_{4,s}$ are supplementary variables, respectively representing the concentration from chemical transport model (CTM), elevation, wind speed, and sunshine hours; the choice of supplementary variables mainly follows Horálek et al.[42], yet the surface solar radiation in Horálek et al.[42] is replaced by sunshine hours subject to data availability; $x_{5,s} = \begin{cases} 0, & x_{1,s} \in \text{LAEI data} \\ 1, & \text{otherwise} \end{cases}$ indicates the data source of $x_{1,s}$, which is additionally used to control for any systematic modelling differences between London Atmospheric Emissions Inventory (LAEI) data and Pollution Climate Mapping (PCM) modelled data; η_s is the regression residual. All of the supplementary data in Eq. S8-1 is pre-processed to be unified to 1-km grids; the values of explanatory variables for each monitoring site are assigned based on the grid cell that contains that site.

A backward elimination approach is additionally applied to further select the variables that can bring useful information to model the target variable[43]. The process begins with all variables in Eq. S8-1 and eliminates one variable at a time from the model. At each step, the variable that makes the least contribution to the model performance is removed. This means that if the model performance is maximised with a particular variable removed, this variable does not support the model with useful information and therefore

---

[§] We also explored an alternative approach by using different target variables in the mapping model, however, its results were less satisfactory in terms of reproducing the spatial distribution of lockdown impacts. The alternative method involved interpolating the annual average concentrations at individual monitoring sites to the unified grids and then to the MSOAs; the lockdown impacts at a particular MSOA are estimated using interpolated air quality levels at this place in the with- and without- lockdown scenarios.

needs to be abandoned. The process runs until the model cannot be improved or no explanatory variable is left. The linear regression model is then re-estimated with the best combination of explanatory variables derived from the backward elimination. The ordinary least squares (OLS) estimate of the coefficients $\{\hat{c}, \hat{a}_1, \hat{a}_2, \ldots, \hat{a}_5\}$ in the final model is stored. The regression residuals at individual monitoring sites, $\{\eta_s\}$, are computed and used as the input of ordinary kriging.

S8.2 Ordinary kriging

Ordinary kriging is the most commonly used geostatistical method. Ultimately, it estimates the value at an unsampled point v_0 as a weighted linear combination of the measurements in the neighbourhood region. Specifically, suppose $Z(\cdot)$ is the variable of interest and $\{v_i: i = 1,2,\ldots,n\}$ as the sample points, ordinary kriging estimates the value at v_0 by $Z(v_0) = \sum_{i=1}^{n} \lambda_i Z(v_i)$, and $\sum_{i=1}^{n} \lambda_i = 1$, where λ_i is the weight associated with v_i. The constraint $\sum_{i=1}^{n} \lambda_i = 1$ is introduced to ensure an unbiased estimate[44]. The weights $\boldsymbol{\lambda}$ are determined by solving a constraint optimisation problem using the method of Lagrange multiplier; specifically, the optimal weights satisfy the following equation system[45,46]:

$$\sum_{i=1}^{n} \lambda_i \gamma(v_i - v_j) + \mu = \gamma(v_j - v_0) \text{ for all sample points } v_j \text{, and}$$

$$\sum_{i=1}^{n} \lambda_i = 1, \qquad \text{Eq. S8-2}$$

where $\gamma(v_i - v_j)$ is the semivariance between the sample point v_i and v_j; $\gamma(v_j - v_0)$ is the semivariance between the sample point v_j and the target point v_0; μ is a Lagrange multiplier.

In Eq. S8-2, the semivariances are derived from a fitted variogram $2\gamma(\cdot)$. The variogram $2\gamma(h)$, or semivariogram $\gamma(h)$, describes the variance between two measurements separated by a distance, that is $2\gamma(h) = 2\gamma(v_i - v_j) = \text{Var}[Z(v_i) - Z(v_j)]$ for all pairs of sample points, where h represents the distance between two points and is commonly binned into several intervals, called lags[44]. If the variogram is only a function of $||h||$ regardless of direction, $2\gamma(h)$ is then called isotropic[44]. The *nugget*, *sill*, and *range* are key parameters in a (semi-)variogram, see an illustrative definition in Figure S8-1. Particularly, the nugget value indicates the measurement error and/or the microscale variation at the locations that are closer than the minimum sampling distance (lag)[46]. The range value indicates the size of the search window; locations farther apart than the range value are not spatially correlated. The sill is the value at which the spatial variance does not change with distance anymore[43]. For use in a kriging model, the empirical variogram needs to be fitted to a theoretical variogram function that characterises the underlying spatial structure, where the spherical model is the most commonly used mathematical function[43].

In our study, an ordinary kriging model is constructed to interpolate the residuals of the linear regression model in Eq. S8-1, $\{\eta_s\}$, to the unified grids. Specifically, the theoretical variogram is fitted to an isotropic spherical model following Horálek et al.[42,43]. Five key parameters in the theoretical variogram, and

consequently also for the corresponding ordinary kriging model, are automatically tuned with Bayesian optimisation; the description and search domain of the tuned parameters are shown in Table S8-1. The generalisation performance of the ordinary kriging model is used as the objective function of parameter tuning, which is evaluated by the rooted mean squared error (RMSE) from a leave-one-out cross-validation (CV). The set of parameters that gives the smallest CV RMSE within 100 iterations is used to fit the final ordinary kriging model. The regression residuals at individual grid cells are then estimated with the final ordinary kriging model and stored as $\{\hat{\eta}_g\}$, where g is the index of the grid cell. The geostatistical model estimation and prediction are implemented in python with the ***SciKit-GStat*** library[47]. The parameter tuning is implemented in python with the ***scikit-learn*** library[27].

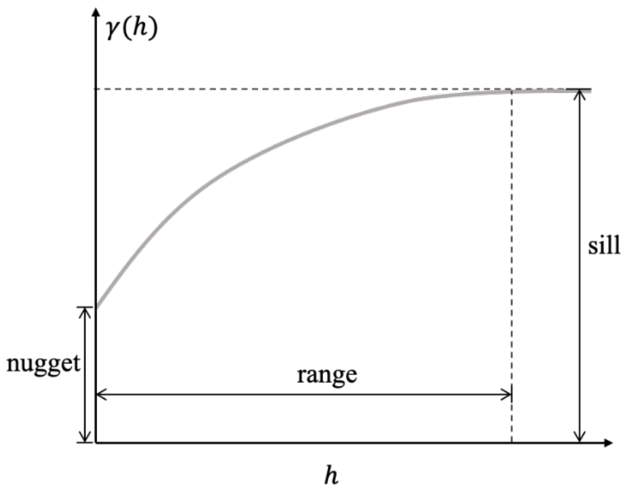

Figure S8-1. Variogram parameters: nugget, range, and sill.

Table S8-1. Description and search domain of key parameters tuned in ordinary kriging.

Parameter [(a)]	Description	Search domain
bin_func [(b, c)]	rules to binning the distance	[‘even’, ‘scott’, ‘fd’, ‘doane’], categorical
n_lags [(c)]	the number of lags to be defined by the binning	[5, 33] [(e)], integer
maxlag [(c)]	maximum distance for the last bin	[0.001, 0.999] [(f)], float
range	effective range [(d)]	[3, 50], float
nugget:sill ratio [(d)]	the ratio of *nugget* value to *sill* value	[0.1, 0.9], float

(a) The name of the *italic* parameters is aligned with those used in the SciKit-GStat library in python.
(b) Binning rules: ‘even’: evenly spaced bins; ‘scott’: derive the number of bins by Scott’s rule; ‘fd’: derive the number of bins by Freedman-Diaconis estimator; ‘doane’: derive the number of bins by Doane’s rule. The label of the binning rules is aligned with those used in the SciKit-GStat library in python.
(c) 'even' method uses two parameters to calculate the bins from the distance matrix: *n_lags* and *maxlag*; 'scott', 'fd', and 'doane' methods only use *maxlag,* where the input *n_lags* would be overwritten.

(d) All models in the SciKit-GStat library use the effective range as an input parameter instead of the range; the effective range is the distance at which 95% of sill is approached.

(e) The upper bound is the total number of sample points.

(f) If 0 < *maxlag* < 1, then the input *maxlag* is relative; the value of *maxlag* (input) * maximum distance will be used as *maxlag* parameter.

S8.3 Combining results

Combining the results from the linear regression model and ordinary kriging, the target variable at individual grid cells is estimated by:

$$\hat{L}_g = \hat{c} + \sum_1^5 \hat{a}_{i,g} x_{i,g} + \hat{\eta}_g, \qquad \text{Eq. S8-3}$$

where $\hat{L}_g$ is the estimated target variable at grid cell g; $\hat{c}$, $\hat{a}_1$,$\hat{a}_2$,..., $\hat{a}_5$ are the estimated coefficients of the linear regression model in Eq. S8-1, where the coefficient of the variables that are removed by the backward elimination equals zero; $x_{i,g}$, $i = 1,2,...,5$ are respectively the explanatory variables in Eq. S8-1 at grid cell g; $\hat{\eta}_g$ is the estimated regression residual based on the ordinary kriging.

S9. Results for mapping lockdown impacts

This section discusses the results of mapping the lockdown impacts to individual MSOAs. The lockdown impact estimates are plotted both at the MSOA and monitoring site levels for comparison, as shown in Figure S9-1. The results suggest that our mapping approach can generally reproduce the spatial distribution of the lockdown impacts.

Moreover, Table S9-1 summarises the model parameters and performance for the mapping approach employed in our study. The results show that the performance of our approach is generally comparable with that of Horálek et al.[42]; specifically, the normalised RMSE (NRMSE) of our approach is comparable with the relative mean uncertainty of the mapping models for NO_2 in their study.

The mapping results are further reviewed to consider the uncertainty in the spatial interpolation process. While no pollution increases were found at the monitoring site level (c.f. Figure 2a in the manuscript), some MSOAs are estimated to experience an increase in annual average NO_2 concentrations after the lockdown. These MSOAs are directly excluded for further analysis, as the estimated pollution increases are all small (relative change: < 0.45%; absolute change: < 0.09 μg/m^3) (see Figure S9-1 (a) and (c)). Furthermore, we also exclude the MSOAs with null monitoring sites within 5 km, as an area that is far from all sample points is more likely to be subject to the uncertainty in spatial interpolation. In this case, the feature evaluation for the relative and absolute lockdown impacts is based on the impact estimates at 701 and 704 MSOAs, respectively, as illustrated in Figure S9-2.

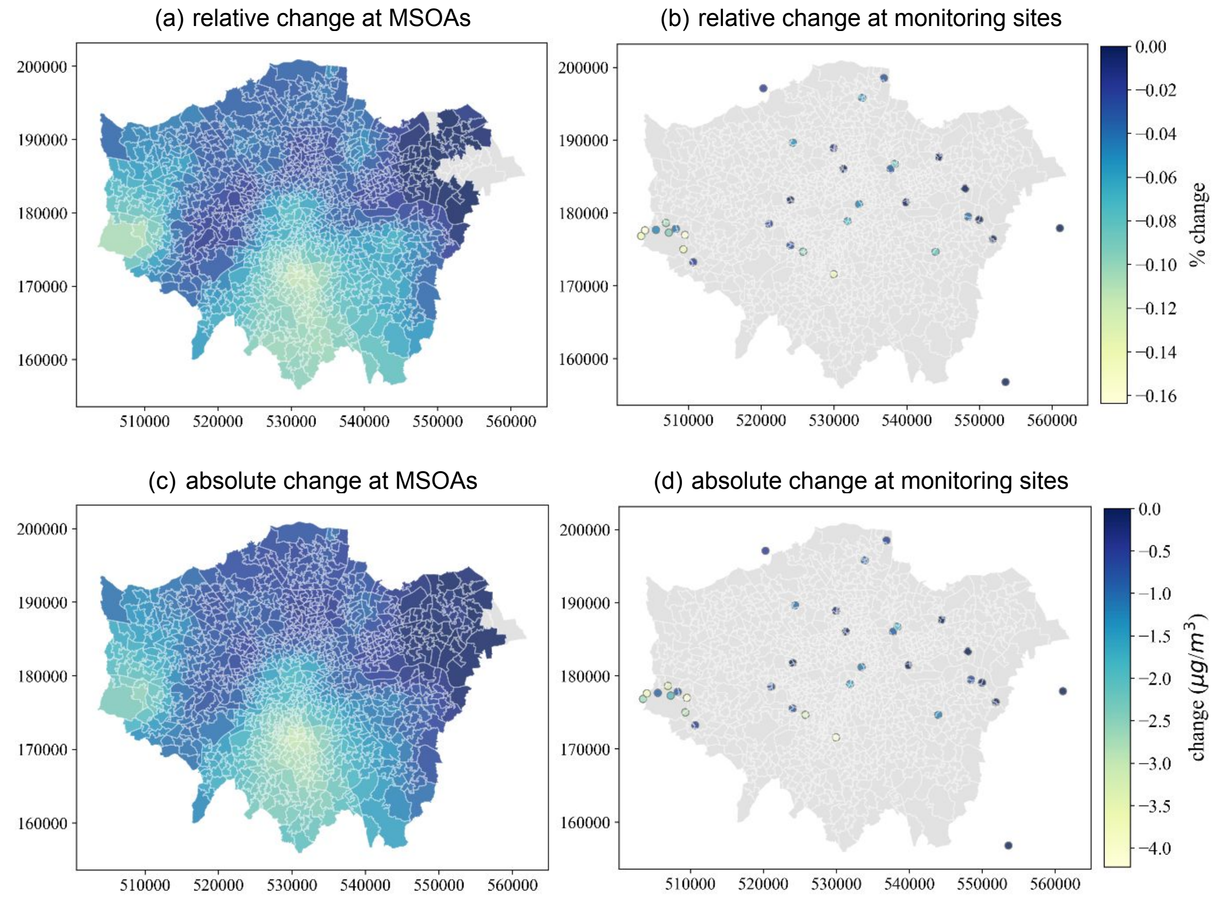

Figure S9-1. Estimated lockdown impacts on the annual average concentrations of NO_2 (above: relative change; below: absolute change). The lockdown impacts at individual monitoring sites (right) are estimated by comparing the NO_2 annual average between the with- and without-lockdown scenarios. The NO_2 annual average at a particular site is estimated using observed concentrations for the with-lockdown scenario and counterfactual concentrations for the without-lockdown scenario; the counterfactual concentrations after the start of the lockdown are scaled from the observed concentrations with the lockdown effect estimate at that site. The lockdown impacts at individual MSOAs (left) are estimated based on spatial interpolation of the impact estimates at monitoring sites. The MSOAs where the estimated lockdown impact showed a contradictory direction of change with the consistent reduction in air pollution at the monitoring site level are excluded and coloured grey.

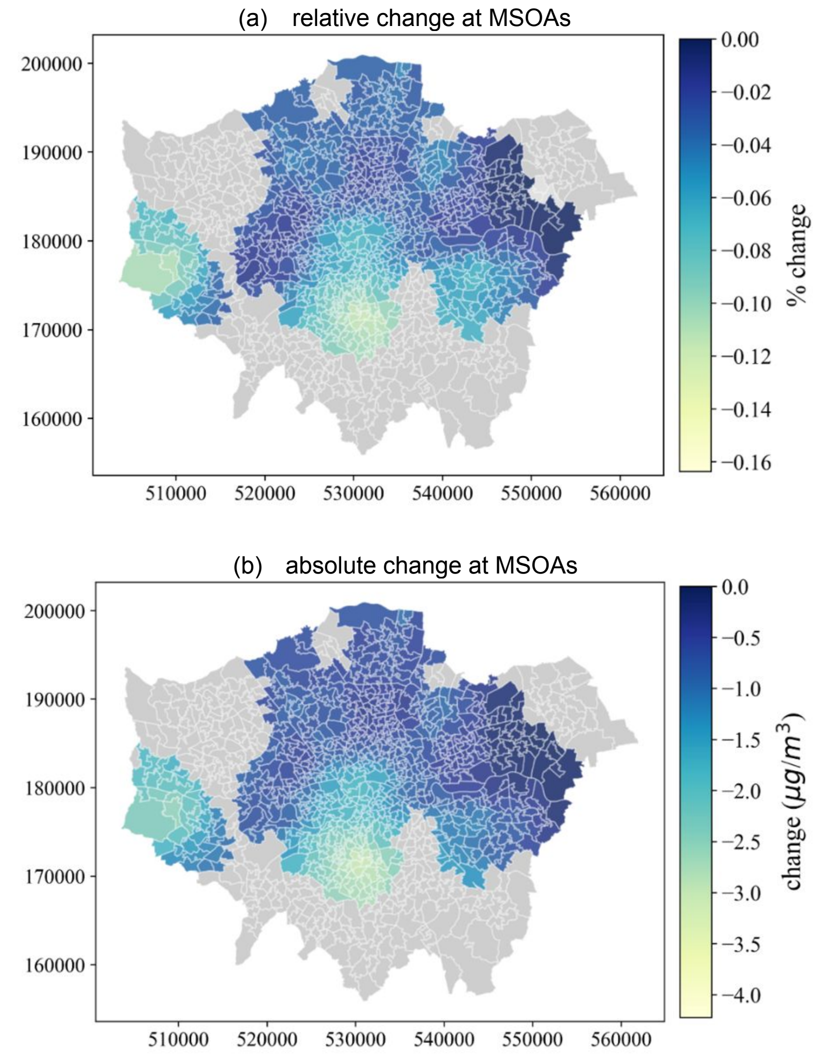

Figure S9-2. Estimated lockdown impacts on the annual average concentrations of NO_2 (above: relative change; below: absolute change) used in feature contribution evaluation. The lockdown impacts at individual MSOAs are estimated based on a spatial interpolation of the impact estimates at individual monitoring sites. The lockdown impact at a particular monitoring site is estimated by comparing the NO_2 annual average between the with- and without-lockdown scenarios, using observed concentrations and counterfactual concentrations respectively. The counterfactual concentrations after the start of the lockdown are scaled from the observed concentrations with the lockdown effect estimate. The MSOAs where the estimated lockdown impact showed a contradictory direction of change (pollution increase/decrease) are excluded. The MSOAs with no monitoring sites within 5 km are excluded. The excluded MSOAs are all coloured grey.

Table S9-1. Parameter and performance statistics of linear regression model and ordinary kriging for mapping the lockdown impact on the NO_2 annual average in 2020.

		Parameters and statistics [(a)]	
		Model for relative change	Model for absolute change
Linear regression	c (constant)	-	-
	x_1 (concentration from CTM)	-0.003***	-0.078**
	x_2 (elevation)	-	-
	x_3 (wind speed)	-0.041**	-0.770*
	x_4 (sunshine hours)	0.000**	0.002*
	x_5 (CTM data source)	-	-
	**adj. R^2**	0.604	0.539
Ordinary kriging of residuals	range	50.000	29.410
	sill	0.001	1.168
	nugget	0.000	0.404
Linear regression + ordinary kriging of residuals [(b)]	**RMSE**	0.041	1.051
	**NRMSE** [(c)]	0.251	0.249

(a) Model parameters that are excluded by backward elimination are marked with '-'. Statistical significance of model parameters: *** Significant at the 1% level; ** Significant at the 5% level; * Significant at the 10% level.

(b) Generalisation performance is evaluated with a leave-one-out cross-validation.

(c) Normalised rooted mean squared error (NRMSE): the RMSE is further normalised by the range of the target variable across the sample points.

S10. Feature evaluation: methods and results

In this section, two methods are introduced for identifying the important contributing factors to predict the magnitude of lockdown impacts at different locations. The first method (§S10.1) relies on a widely used feature importance metric (total gains). The second method (§S10.2) utilises explainable machine learning and has a better interpretation of feature contribution values. The second method is applied to present the results in the manuscript.

S10.1 Measuring feature importance with total gains

After the spatial interpolation, we build a gradient boosting decision trees (GBDT) model on the estimated lockdown impact at different MSOAs, separately for the absolute and relative impacts. Explanatory variables used in the GBDT model include 124 features covering various aspects of individual MSOAs (c.f. Table S1-1). Notably, as the estimated lockdown impacts either show a pollution decrease or a null effect (c.f. Figure 2a in the manuscript), the GBDT model is then fitted on the arithmetic absolute value of the impact estimates to improve explanation in the following steps. In other words, the GBDT model is a predictive model for the magnitude of pollution reduction caused by the lockdown.

For each GBDT model, four key hyperparameters are automatically tuned by Bayesian optimisation with 100 iterations. The description and search domain of the tuned hyperparameters are shown in Table S10-1. The generalisation performance of the GBDT model is used as the objective function of hyperparameter tuning, which is evaluated by rooted mean squared error (RMSE) from a 5-fold cross-validation (CV). The GBDT model training and hyperparameter tuning are implemented in python with the ***lightGBM*** library[26] and ***scikit-learn*** library[27], respectively. Note that the number of boosted trees (*n_estimator*), which is another key hyperparameter, is determined by the early stop function in the ***lightGBM*** library. The feature importance values are given by the ***lightGBM*** library, which represents the total gains of splits that use a particular feature; further details for the variance gain of a node's split can be found in Ke et al.[26]. The values of feature importance are further normalised to sum to 1, i.e. relative importance.

The most important features given by the total gains approach are illustrated in Figure S10-1. The features with relative importance greater than 0.01 are additionally listed in Table S10-2. Notably, as the value of feature importance evaluated by total gains does not imply a practical interpretation, it is difficult to quantitatively evaluate the role of the factors, such as the distance to the nearest metropolitan town centre (Table S10-2), in affecting the magnitude of lockdown impact.

The proportion of heavy goods vehicles (HGVs) in road traffic, the distance to London Heathrow Airport, and the distance to the Central Activities Zone (central London) are highlighted as the most important contributing factors, both to the absolute and relative lockdown impacts. This is consistent with the top features highlighted by the feature importance using SHAP values, as shown in the manuscript and SI §S10.2. The sum of the relative importance of the top two features exceeds 0.65 for both cases, which

implies the importance of controlling emissions from airport-related activities and road freight transport in the future. Other features that are important to both impact indicators include median gross annual pay (residents or workplace) and the employment size for accommodation and food service activities. In addition, two absolute features for road traffic (the traffic volume of HGVs and that of all motor vehicles) are highlighted for absolute lockdown impact, which could imply the importance of managing absolute traffic demand while reducing emission factors. Meanwhile, the distance to the nearest metropolitan town centre is highlighted for relative lockdown impact; however, as mentioned before, it is difficult to quantify how this feature affected the magnitude of lockdown impact.

While the top 5 features given by SHAP values and total gains are similar for both the relative and absolute lockdown impacts, some features become more important when ranked by SHAP values compared with total gains (c.f. Figure 3 in manuscript and SI §S10.2). For example, for the relative lockdown impact, the proportion of the population aged over 65 (labelled as [Popu]_65+_%) and the proportion of light goods vehicles in road traffic before the lockdown (labelled as [TraV]_lcv%) have a higher rank when considering SHAP values; for the absolute lockdown impact, the public transport accessibility level (labelled as [PuT]_BYAI) becomes more important in the ranking of SHAP values. The difference in rank could be related to how different metrics perceive feature importance: while SHAP values focus on the marginal changes in output prediction brought by a feature, total gains focus on the contribution of a given feature to model error reduction[48].

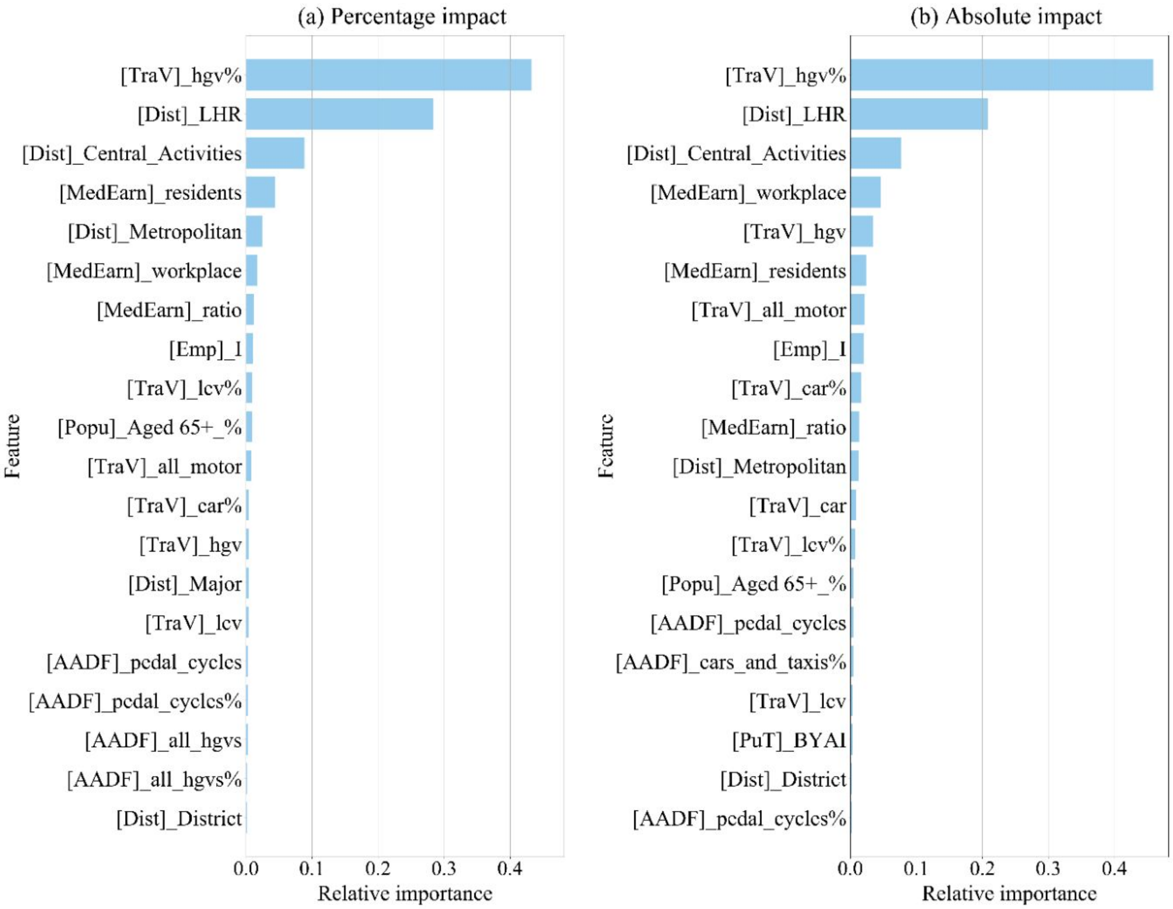

Figure S10-1. Estimated top 20 features in predicting the relative lockdown impact (left) and the absolute lockdown impact (right) on NO_2 annual average concentrations using the total gains approach. A GBDT model is built on the reductions in NO_2 annual average concentrations at different MSOAs with a group of features. Feature importance is estimated by the total gains of splits in the fitted GBDT model that use the feature. The values of feature importance are further normalised to sum to 1, i.e. relative importance (x-axis). Features are sorted by relative importance.

Table S10-1. Description and search domain of key hyperparameters in the GBDT model of lockdown impact.

Hyperparameter [(a)]	Description	Search domain
max_depth	maximum tree depth for each tree	[1, 10], integer
subsample	subsample ratio of training instances for each tree	[0.8, 1], float
learning_rate	controls the step size at each iteration towards the direction of the negative gradient	[0.005, 0.5], float
colsample_bytree	subsample ratio of columns (i.e. variables) when constructing each tree	[0.2, 1], float

(a) The name of hyperparameters is aligned with those used in the lightGBM library in python.

Table S10-2. Important contributing factors to predicting the relative and absolute lockdown impacts on NO_2 annual average concentrations (total gains approach).

	Feature label [(a)]	Feature name	Relative Import. [(b)]
Relative impact	[TraV]_hgv%	Proportion of HGVs in road motor vehicle traffic	0.43
	[Dist]_LHR	Distance to London Heathrow Airport	0.28
	[Dist]_Central_Activities	Distance to Central Activities Zone	0.09
	[MedEarn]_residents	Median of gross annual pay for residents	0.04
	[Dist]_Metropolitan	Distance to the nearest metropolitan town centre	0.02
	[MedEarn]_workplace	Median of gross annual pay for workplace	0.02
	[MedEarn]_ratio	Ratio of median gross annual pay (workplace/residents)	0.01
	[Emp]_I	Employment in Industrial Section I [(c)] (Accommodation & Food Service Activities)	0.01
Absolute impact	[TraV]_hgv%	Proportion of HGVs in road motor vehicle traffic	0.46
	[Dist]_LHR	Distance to London Heathrow Airport	0.21
	[Dist]_Central_Activities	Distance to Central Activities Zone	0.08
	[MedEarn]_workplace	Median of gross annual pay for workplace	0.05
	[TraV]_hgv	Traffic volume of HGV	0.03
	[MedEarn]_residents	Median of gross annual pay for residents	0.02
	[TraV]_all_motor	Traffic volume of all motor vehicles	0.02
	[Emp]_I	Employment in Industrial Section I [(c)] (Accommodation & Food Service Activities)	0.02
	[TraV]_car%	Proportion of car and taxi in road motor vehicle traffic	0.02
	[MedEarn]_ratio	Ratio of median gross annual pay (workplace/residents)	0.01
	[Dist]_Metropolitan	Distance to the nearest metropolitan town centre	0.01

(a) Features with relative importance greater than 0.01; ranked by the value of relative importance.
(b) Feature importance is evaluated based on a fitted GBDT model. The importance of a particular feature is derived by estimating the total gains of splits that use the feature. The values of feature importance are further normalised to sum to 1, i.e. relative importance.
(c) Industrial section is classified based on the UK Standard Industrial Classification hierarchy.

S10.2 Measuring feature importance with SHAP values

In addition to total gains, *permutation importance* and *total splits* are also popular metrics for evaluating feature importance in a fitted tree-based model[48]. While total splits rank features by counting the total number of splits that use a particular feature, permutation importance focuses on the change in model performance when the values of a feature are randomly permutated[48]. Therefore, current popular metrics for feature importance either rely on the frequency of usage in the model (total splits) or focus on the contribution of a particular feature to error reduction (total gains and permutation importance). These metrics enable the estimation of feature ranking and identification of the most important features. However, their results cannot directly suggest how the magnitude of the output variable in the model is affected. To have a better interpretation of the relationship between lockdown impacts and individual features, a more complex approach is necessary to explain the predictive GBDT model built in §S10.1.

Different methods have been recently developed to interpret complex machine learning models[49–52], and many of these methods fall into the class of *additive feature attribution methods*[53]. This class of methods is characterised by using an explanation model g to approximate the original prediction model f, where g is a linear function of binary variables[53]. Specifically, suppose x is an instance/sample/row in the original regressor matrix. For a single prediction $f(x)$, $g(z)$ is represented by[53]:

$$g(z) = \phi_0 + \sum_{i=1}^{M} \phi_i z_i, \qquad \text{Eq. S10-1}$$

where $z \in \{0,1\}^M$, and z_i implies whether feature i is observed or unknown in instance x; $x = h_x(z)$ is a mapping function that maps between the binary state and the original input space; M is the number of original input features; $\phi_i \in \mathbb{R}$. Notably, Eq. S10-1 is a local prediction explanation where $\{\phi_i\}$ is specific to a single instance x (also for prediction $f(x)$).

As indicated in Eq. S10-1, an additive feature attribution method assigns a contribution ϕ_i to each feature, and the sum of $\{\phi_i\}$ approximates the original prediction. Therefore, ϕ_i is in the same unit as the model's output and, consequently, can provide a better interpretation compared with using total gains. Previous studies have shown that among the methods of this class, Shapley values provide a single unique solution to the problem of assigning contributions that guarantees the model g has some desirable properties: local accuracy (efficiency), missingness (null effects and symmetry), and consistency (monotonicity)[53,54].

The Shapley value was originally proposed in the 1950s[55]. It is a key solution method in cooperative game theory to allocate the total payoff generated by a coalition of players to individual players according to their contributions. In the context of model interpretation, the input features can be regarded as the players and the output prediction as the total payoff. The Shapley value of feature i for an instance x can be computed by

$$\phi_i = \sum_{S \subseteq N \setminus \{i\}} \frac{|S|!(|N| - |S| - 1)!}{|N|!} [f_x(x_{S \cup \{i\}}) - f_x(x_S)], \qquad \text{Eq. S10-2}$$

where N is the set of all input features; S is a subset of N, which represents a possible coalition; x_S represents the value of the features in S; $f_x(x_S)$ is the output of model f predicted with x_S, where the complement features x_c are integrated out. As shown in Eq. S10-2, the Shapley value is a weighted average of the feature's marginal contribution across all possible coalitions of the input feature set; the weights are assigned based on the number of features in each coalition and the feature ordering matters[53,54,56].

The main disadvantage of using Shapley values is the computation cost, which increases exponentially with the number of input features. Some computationally efficient algorithms have been proposed to approximate the Shapley value[53,54], and many of them have been compiled into a python library, ***SHAP*** (SHapley Additive exPlanations).

In this paper, we use the ***SHAP*** library in python to approximate the Shapley values and interpret the GBDT models built in §S10.1. Specifically, the algorithm of *TreeExplainer* with interventional feature permutation[54] is applied with the ***SHAP*** library to estimate the contribution of individual features for each prediction output of the model. This algorithm defines the value function in Eq. S10-2 using the marginal distribution, that is $f_x(x_S) = E[f(X)|\mathrm{do}(X_S = x_S)]$, where the values of the hidden features x_c are replaced with a random value drawn from the original input dataset[54,56]; note that Lundberg et al.[54] started to use the do-calculus notation following a causal inference framework to distinguish observational and interventional conditional probabilities, as suggested in Janzing et al.[57]. The estimated contributions of features derived from the ***SHAP*** library are called "SHAP values" in this paper. Figure S10-2 illustrates the relationship among the SHAP value, the marginal distribution, and the prediction output.

The estimated feature contributions using SHAP values are summarised in Figure 3 in the manuscript for explaining the relative lockdown impacts, while Figure S10-3 in the SI illustrates the estimated SHAP values for the absolute lockdown impacts.

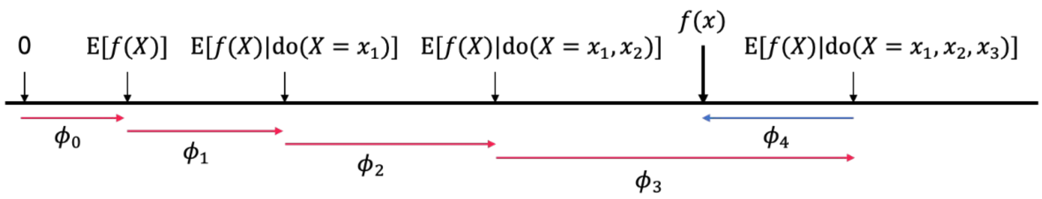

Figure S10-2. Graphical example of the SHAP value of features specific to an instance. The SHAP values are used to explain a particular prediction output, $f(x)$, of a model f. Positive SHAP values are indicated with red arrows and negative SHAP values are indicated with blue arrows. The sum of the SHAP value of all features, $\sum_{i=1}^{4}\phi_i$, indicates the difference between the prediction $f(x)$ and the base value ϕ_0. The base value ϕ_0 is commonly set by the mean prediction across all instances, that is $\mathrm{E}[f(X)]$.

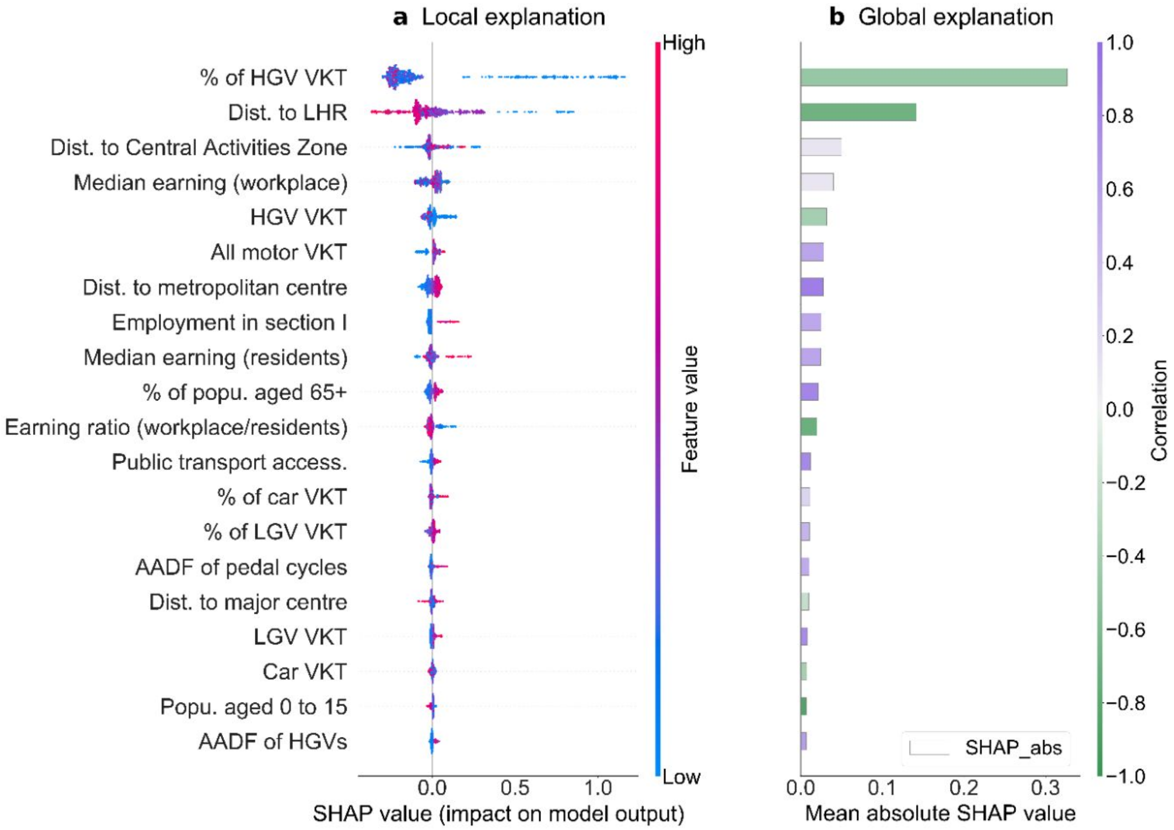

Figure S10-3. Estimated local SHAP values (left) and global feature importance (right) quantifying the contribution of each feature to the absolute reduction (μg/m^3) in NO_2 concentrations due to the COVID-19 lockdown. **a** Each point is a SHAP value specific to a feature and an MSOA; the colour of a point indicates the feature's value for that MSOA; points are spread out in the y-dimension to avoid overlap. **b** The global importance of a particular feature is calculated as the mean absolute SHAP value across all the MSOAs; the colour of bars indicates the Pearson correlation between the value of a feature and the corresponding SHAP value across different MSOAs: a negative correlation (green) indicates that an MSOA with a higher value of that feature is generally associated with a smaller pollution reduction, while a positive correlation (purple) indicates that an MSOA with a higher feature value is generally associated with a larger pollution reduction. Features are sorted by the global feature importance; the top 20 features with the most significant global importance value are plotted.

S11. Putting COVID-19 attributable changes in context

This section discusses the changes in air pollution levels in London around the start of the lockdown and compares them with the detected change points that occurred at other time periods. This enables us to provide an insight into the changes associated with the COVID-19 lockdown in the context of the longer-term trend of air quality in London. The concentration changes are evaluated based on the normalised concentrations, which take account of the influences of meteorological conditions and seasonality effects.

To show the overall trends of London's air pollution levels in recent years, we aggregate the normalised concentration time series in 2016-2020 across different monitoring sites for individual air pollutants, as illustrated in Figure 1 in the manuscript for NO_2 and Figure S11-1 to Figure S11-4 in the SI for other pollutants. The results indicate a noticeable decreasing trend in NO_2 and NO_x concentrations in London since 2016, especially at roadside locations. The concentrations of PM_{10} and $PM_{2.5}$, particularly those at roadside sites, were also reduced in general, though less rapidly than NO_2 and NO_x. Meanwhile, the results also suggest that O_3 concentrations in London have generally increased over this period; however, this might be related to the decreasing trend in NO_x concentrations due to the chemical coupling of these pollutants[58].

Road transport accounts for around 50% of both NO_2 pollution and NO_x emissions in London[59]. Following a dramatic reduction in road traffic led by travel restrictions, an abrupt decrease in concentrations of NO_2 and NO_x was clearly observed around the start of the lockdown, especially at the roadside sites (Figure 1 in the manuscript and Figure S11-1). However, concurrent changes in O_3 (Figure S11-2), PM_{10} (Figure S11-3), and $PM_{2.5}$ (Figure S11-4) concentrations were less noticeable in London. Unlike NO_2 and NO_x, road transport has a relatively smaller contribution to PM in London, accounting for about 26% and 30% of PM_{10} and $PM_{2.5}$ emissions, respectively[21]. The situation for O_3 was more complex, because O_3 is not directly emitted from human activities, but is generated through nonlinear chemical reactions among other pollutants (such as NO_x and volatile organic compounds) in the presence of enough heat and sunligh[60]. Moreover, regional contributions to concentrations of O_3, PM_{10}, and $PM_{2.5}$ are substantial in London[60].

To compare the changes in concentrations around the start of the lockdown with those in previous years, we truncate each normalised concentration time series into segments based on the detected change points and evaluate the concentration change at each change point by comparing the mean normalised concentrations in the segments before and after that point. These concentration changes are then aggregated and compared based on whether the chane point occurred in response to the COVID-19 lockdown or in other time periods. Here, the COVID-related change points are referred to those located within the margin period defined in our study, which was 3 weeks before and after the lockdown (c.f. response identification in SI §S5.1). In cases where a site had multiple COVID-related change points, we calculate the COVID-related change by comparing the mean concentrations between the pre-segment of the first COVID-related change point and the post-segment of the last COVID-related change point. In other

words, we focus on the concentration change indicated by this group of change points, rather than those associated with each individual point.

It is important to note that the concentration changes quantified in this section are based on comparing segment means before and after a certain point in time. In this case, the lockdown-related concentration changes mentioned here can incorporate the longer-term trend and, therefore, are not necessarily indicative of the causal impacts of the lockdown. However, we use these estimates to enable a fair comparison among change points in different time periods. To isolate the causal air quality impacts of the lockdown, we rely on a causal inference model, specifically a sharp RDD in time, in our paper. The results for the estimated causal impacts are discussed in the manuscript and additional details are provided in SI §S12 - §S15.

We summarise the results in Figure S11-5 and Figure S11-6 to compare the concentration changes between the COVID-related changes and those in other time periods. While Figure S11-5 indicates the direction of changes (concentration increase or decrease), Figure S11-6 focuses on the magnitude of these changes. For NO_2 and NO_x, all COVID-related change points consistently indicated a pollution decrease, while a small number of non-COVID-related change points were associated with an increase in air pollution. Moreover, as the COVID-19 lockdown led to unpresedented changes in activities, the magnitude of concentration changes associated with the lockdown was generally larger than those that occurred in other periods; specifically, the median of COVID-related concentration changes was generally greater than the 90th and 72nd percentiles of non-COVID-related changes for NO_2 and NO_x, respectively. Similar to NO_2 and NO_x, changes in O_3 concentrations associated with the COVID-19 lockdown generally showed a more consistent direction of change and greater magnitude compared to those in other periods. Particularly, all change points associated with the COVID-19 lockdown indicated an increase in O_3 concentrations. Moreover, the smallest COVID-related change in O_3 concentrations was greater than the 78th and 50th percentiles of non-COVID-related changes in magnitude for background and roadside concentrations of O_3, respectively. However, in contrast to other pollutants analysed in our study, the concentration changes for PM_{10} were generally smaller in magnitude, and the mean changes were centred around zero for both COVID-related and non-COVID-related change points. In addition, the magnitude of COVID-related changes in PM_{10} concentrations were generally smaller than those that occurred in other periods. Note, that Figure S11-5 and Figure S11-6 do not cover the results for $PM_{2.5}$, as no COVID-related change points were found for this pollutant.

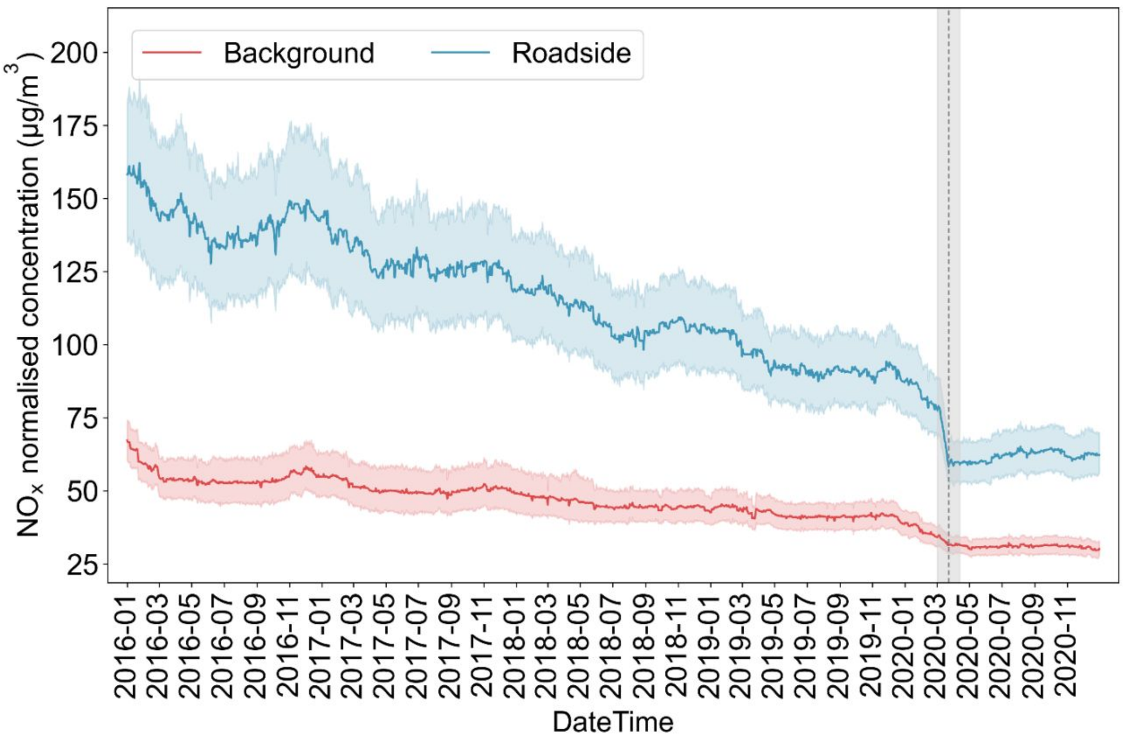

Figure S11-1. Daily average NO_x concentrations in London from 2016 to 2020 by monitoring locations (red: background sites; blue: roadside sites). Concentrations in London and the corresponding 95% CI are estimated by averaging across the concentrations at individual monitoring sites with bootstrapping. Air pollutant concentrations at individual monitoring sites are normalised to remove the influences of meteorological conditions and seasonality effects. The start of the lockdown is indicated with the vertical line. The margin period, which is a symmetric period around the start of the lockdown for response identification, is shaded grey.

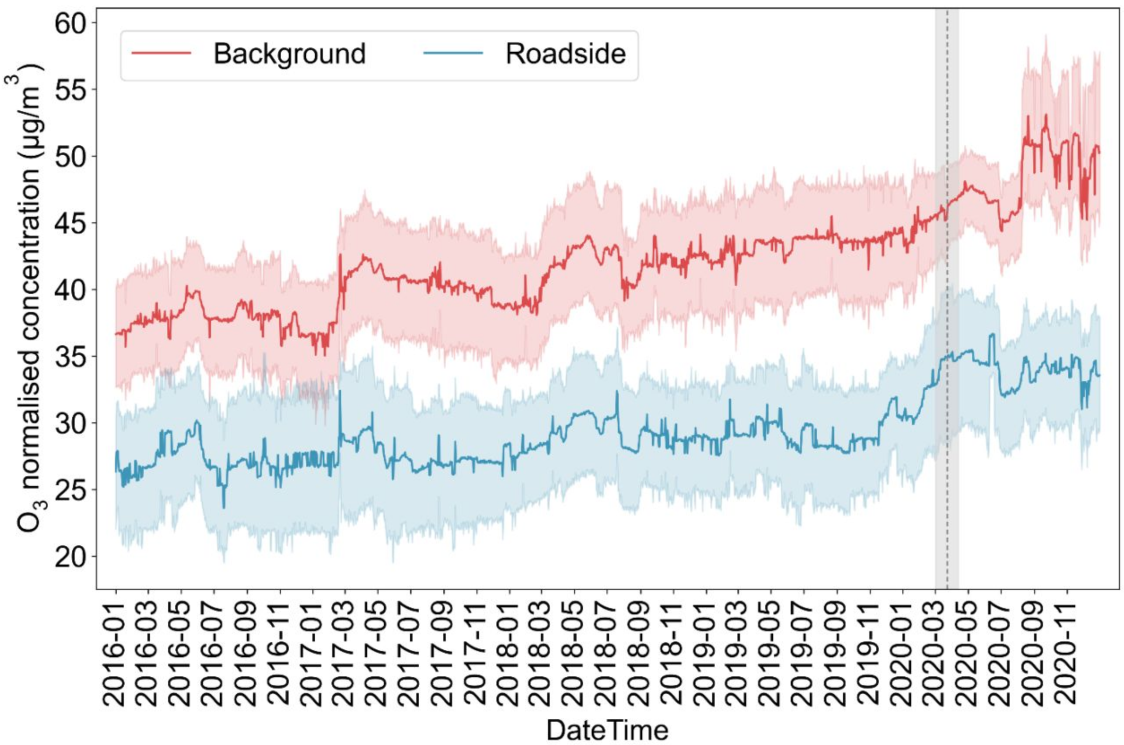

Figure S11-2. Daily average O_3 concentrations in London from 2016 to 2020 by monitoring locations (red: background sites; blue: roadside sites). Concentrations in London and the corresponding 95% CI are estimated by averaging across the concentrations at individual monitoring sites with bootstrapping. Air pollutant concentrations at individual monitoring sites are normalised to remove the influences of meteorological conditions and seasonality effects. The start of the lockdown is indicated with the vertical line. The margin period, which is a symmetric period around the start of the lockdown for response identification, is shaded grey.

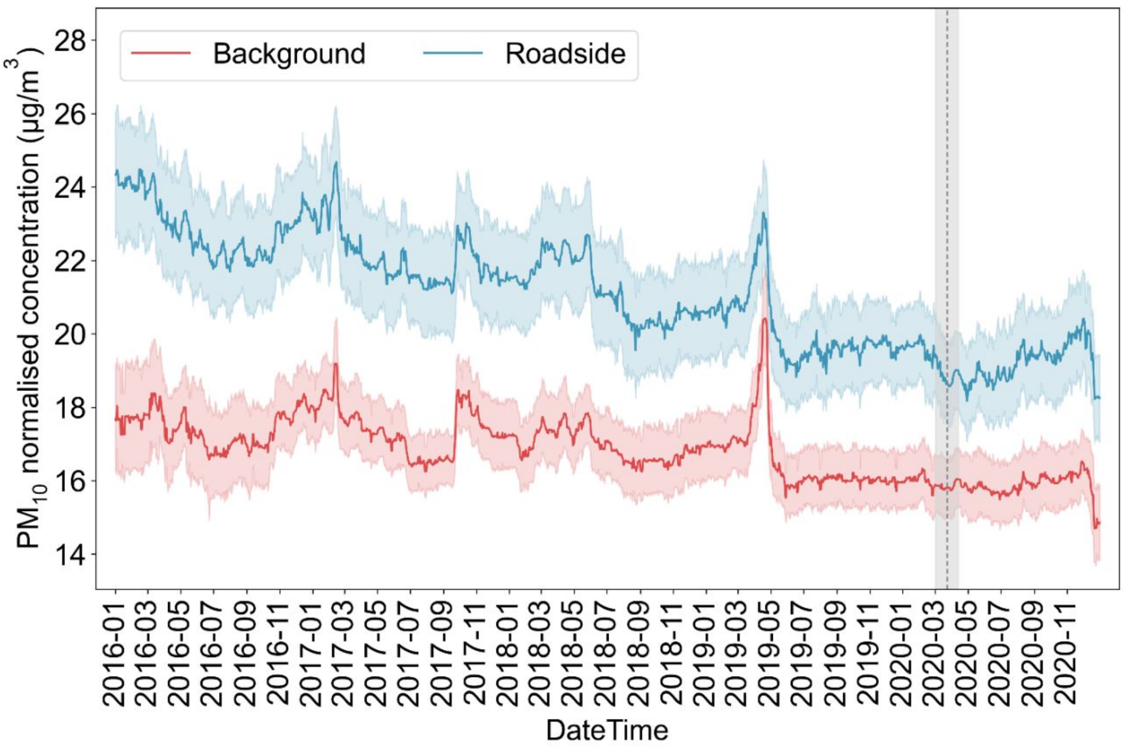

Figure S11-3. Daily average PM_{10} concentrations in London from 2016 to 2020 by monitoring locations (red: background sites; blue: roadside sites). Concentrations in London and the corresponding 95% CI are estimated by averaging across the concentrations at individual monitoring sites with bootstrapping. Air pollutant concentrations at individual monitoring sites are normalised to remove the influences of meteorological conditions and seasonality effects. The start of the lockdown is indicated with the vertical line. The margin period, which is a symmetric period around the start of the lockdown for response identification, is shaded grey.

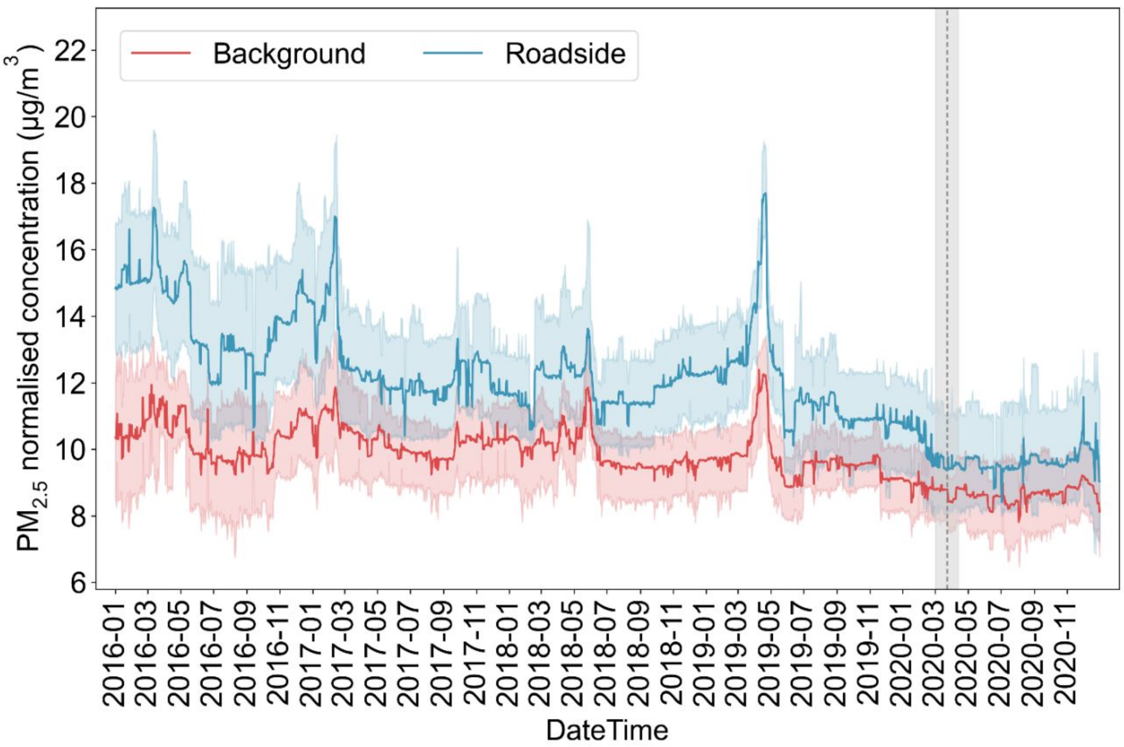

Figure S11-4. Daily average $PM_{2.5}$ concentrations in London from 2016 to 2020 by monitoring locations (red: background sites; blue: roadside sites). Concentrations in London and the corresponding 95% CI are estimated by averaging across the concentrations at individual monitoring sites with bootstrapping. Air pollutant concentrations at individual monitoring sites are normalised to remove the influences of meteorological conditions and seasonality effects. The start of the lockdown is indicated with the vertical line. The margin period, which is a symmetric period around the start of the lockdown for response identification, is shaded grey.

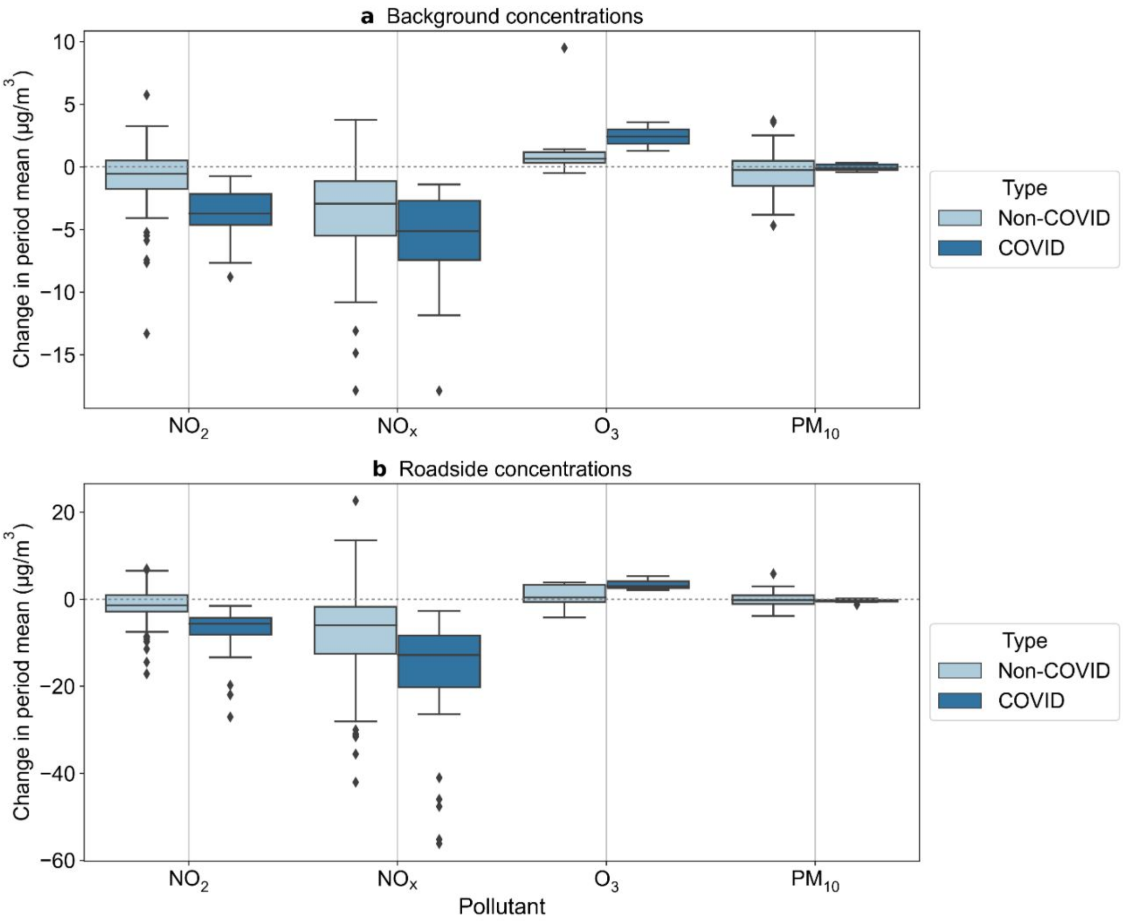

Figure S11-5. Change in concentrations of different air pollutants in London from 2016-01-01 to 2020-12-31. Concentration changes for individual monitoring sites are estimated at individual change points on normalised concentrations, where the influences of meteorological conditions and seasonality effects are removed. The concentration change at a particular change point is determined by segmenting the normalised concentration time series based on detected change points and comparing the mean normalised concentrations between the segments before and after that point. Concentration changes are classified as COVID-related (dark blue) or non-COVID-related (light blue) changes. COVID-related changes are those that occurred within the margin period, which is a symmetric period around the start of the lockdown for response identification.

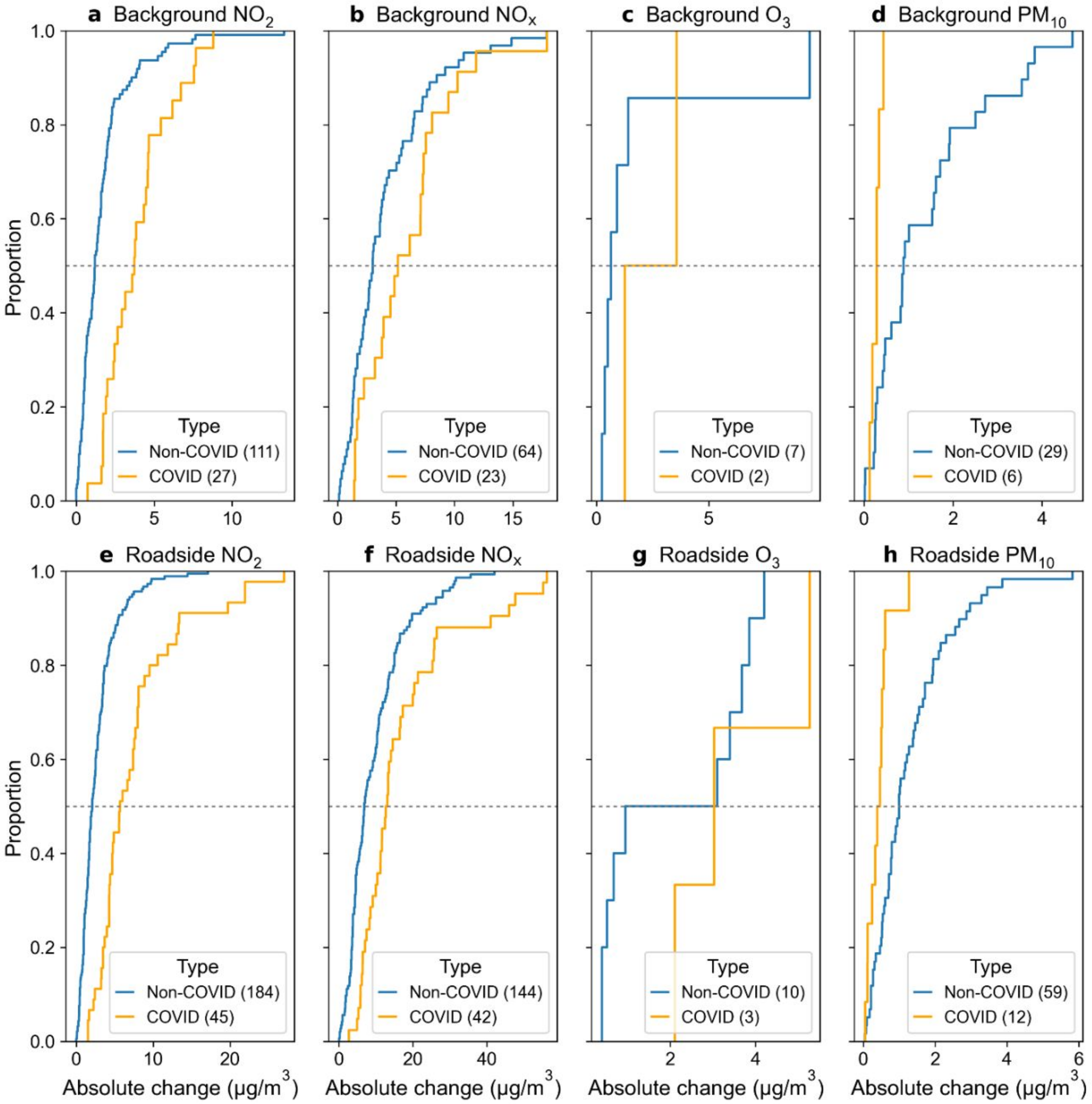

Figure S11-6. Empirical cumulative distribution of the magnitude of concentration changes for different air pollutants in London from 2016-01-01 to 2020-12-31. Concentration changes for individual monitoring sites are estimated at individual change points on normalised concentrations, where the influences of meteorological conditions and seasonality effects are removed. The concentration change at a particular change point is determined by segmenting the normalised concentration time series based on detected change points and comparing the mean normalised concentrations between the segments before and after that point. Concentration changes are aggregated based on COVID-related (dark blue) and non-COVID-related (light blue) changes. COVID-related changes are those that occurred within the margin period, which is a symmetric period around the start of the lockdown for response identification.

S12. Effects on NO_2 concentrations

The estimated effects of the lockdown on NO_2 concentrations at different monitoring sites are illustrated in Figure 2 in the manuscript. These results are summarised in Table S12-1 for the London-wide effects and further summarised in Table S12-2 for individual subregions, including central, inner, and outer London, and the region outside GLA.

The results indicate that all the individual subregions in the analysis experienced a statistically significant average reduction in NO_2 concentrations (roadside or background). The reductions in roadside concentrations of NO_2 were larger than those at background monitoring sites, both at the city level and in individual subregions. In particular, the average reduction in roadside NO_2 concentrations was higher than the corresponding background sites by a factor of 2.8 in both central (22% for roadside; 8% for background) and inner London (14% for roadside; 5% for background). The difference in average reduction between background and roadside concentrations of NO_2 is likely to reflect how the lockdown affected different activities: while roadside concentrations are mainly affected by transport emissions, background concentrations can be affected by various sectors, such as domestic heating, transport, industry, and energy supply emissions sources.

The lockdown consistently caused a significant reduction in NO_2 concentrations at all roadside sites. The average reductions in roadside NO_2 concentrations among different subregions generally decreased with the distance to the city centre; 22% for central London, 14% for inner London, 9% for outer London, and 10% for the sites outside GLA. However, for the background sites, we find a smaller pollution reduction on average and a lower response ratio (5%; 57%) in inner London, compared with central (8%; 100%) and outer London (8%; 95%). In addition, most of the background sites that showed a large decrease in NO_2 concentrations are located around London Heathrow Airport (LHR).

Table S12-1. Summary of the lockdown's effects on NO_2 concentrations across London.

		Background NO_2		Roadside NO_2	
		Total Effect (%) [a, b]	Adj. R^{2} [f]	Total Effect (%) [a, b]	Adj. R^{2} [f]
London	*Response*	*27 of 33 sites*		*45 of 45 sites*	
	std	6.68	0.016	8.81	0.011
	min	-22.83	0.932	-50.04	0.937
	max	0.00	0.998	-2.34	1.000
	mean response [c, e]	-8.80*** [-11.65, -6.22]		-11.74*** [-15.13, -9.18]	
	**regional mean** [d, e]	-7.16*** [-9.97, -4.81]		-11.74*** [-15.13, -9.18]	

(a) The total effect includes the impact from the current period and the stacked impacts from the lagged periods. Interval estimate is simulated with 10,000 Monte Carlo iterations. Standard errors of coefficients are heteroscedasticity and autocorrelation consistent using 7 lags and without small sample correction.

(b) The standard deviation, minimum value, and maximum value are provided with statistically insignificant estimates (at the 10% level) adjusted to zero.

(c) The mean response is the aggregated effect across all sites where the concentrations responded to the intervention.

(d) The regional mean is the aggregated effect across all sites.

(e) The aggregated effect is computed with 1,000 bootstrap resampling iterations. The 95% CI of aggregated effect (in bracket) is the percentile interval of 1,000 bootstrap resampling iterations. Statistical significance: *** Significant at the 1% level; ** Significant at the 5% level; * Significant at the 10% level.

(f) The adjusted R^2 indicates the performance of the RDD model. The standard deviation, minimum value, and maximum value are provided by summarising the model performance across all RDD models.

Table S12-2. Summary of the lockdown's effects on NO_2 concentrations in individual subregions.

		Background NO_2		Roadside NO_2	
		Total Effect (%) [a, b]	Adj. R^{2} [f]	Total Effect (%) [a, b]	Adj. R^{2} [f]
Central London	*Response*	*2 of 2 sites*		*5 of 5 sites*	
	std	1.66	0.002	16.18	0.001
	min	-9.09	0.993	-50.04	0.996
	max	-6.75	0.995	-10.84	1.000
	mean response [c, e]	-7.96*** [-12.68, -3.39]		-21.68*** [-39.97, -9.62]	
	**regional mean** [d, e]	-7.96*** [-12.68, -3.39]		-21.68*** [-39.97, -9.62]	
Inner London	*Response*	*4 of 7 sites*		*12 of 12 sites*	
	std	7.45	0.030	10.34	0.009
	min	-17.45	0.932	-39.10	0.965
	max	-1.60	0.997	-2.81	0.999
	mean response [c, e]	-8.70*** [-16.72, -2.06]		-13.89*** [-21.88, -7.80]	
	**regional mean** [d, e]	-4.97*** [-11.12, -0.50]		-13.89*** [-21.88, -7.80]	
Outer London	*Response*	*18 of 19 sites*		*24 of 24 sites*	
	std	6.47	0.013	4.32	0.013
	min	-22.83	0.955	-17.92	0.937
	max	0.00	0.998	-2.34	0.998
	mean response [c, e]	-8.04*** [-11.51, -4.99]		-8.88*** [-10.98, -6.82]	
	**regional mean** [d, e]	-7.57*** [-10.83, -4.79]		-8.88*** [-10.98, -6.82]	
Outside GLA	*Response*	*3 of 5 sites*		*4 of 4 sites*	
	std	9.67	0.003	3.28	0.004
	min	-20.31	0.989	-12.77	0.985
	max	-2.60	0.995	-6.44	0.996
	mean response [c, e]	-13.82*** [-22.46, -3.55]		-9.69*** [-13.75, -5.63]	
	**regional mean** [d, e]	-8.13** [-16.94, -0.43]		-9.69*** [-13.75, -5.63]	

(a) The total effect includes the impact from the current period and the stacked impacts from the lagged periods. Interval estimate is simulated with 10,000 Monte Carlo iterations. Standard errors of coefficients are heteroscedasticity and autocorrelation consistent (HAC) using 7 lags and without small sample correction.

(b) The standard deviation, minimum value, and maximum value are provided with statistically insignificant estimates (at the 10% level) adjusted to zero.

(c) The mean response is the aggregated effect across all sites where the concentrations responded to the intervention.

(d) The regional mean is the aggregated effect across all sites.

(e) The aggregated effect is computed with 1,000 bootstrap resampling iterations. The 95% CI of aggregated effect (in bracket) is the percentile interval of 1,000 bootstrap resampling iterations. Statistical significance: *** Significant at the 1% level; ** Significant at the 5% level; * Significant at the 10% level.

(f) The adjusted R^2 indicates the performance of the RDD model. The standard deviation, minimum value, and maximum value are provided by summarising the model performance across all RDD models.

S13. Effects on NO_x concentrations

The estimated effects of the lockdown on NO_x concentrations at different monitoring sites are illustrated in Figure S13-1. The results are summarised in Table S13-1 for the London-wide effects and further summarised in Table S13-2 for individual subregions. 83% of the monitoring sites (background: 23/33; roadside: 42/45) within London showed a response in NO_x concentrations to the lockdown. The lockdown changed the daily average background NO_x concentrations by -33% to 0%, with a city-wide mean effect of -6% [-9%, -4%], and the roadside NO_x concentrations by -62% to 0%, with a city-wide mean effect of -11% [-16%, -8%]. All individual subregions showed a statistically significant average reduction in NO_x concentrations, both at roadside and background sites. Compared with background concentrations of NO_x, the lockdown caused a higher average reduction in NO_x at the roadside, which is consistent across all subregions and is particularly obvious in central and inner London.

Comparing the results for roadside NO_x concentrations in different subregions, we find that both central and inner London experienced an average reduction of more than 15% (central: 22%; inner: 15%), while outer London and the region outside the GLA experienced lower average reductions (<10%). The maximum reduction in roadside NO_x concentrations (62%) was at site LAQN_CT4 in central London, which also showed the highest reduction in roadside NO_2 concentrations (c.f. Figure 2 in the manuscript). The second large reduction in roadside NO_x concentrations (42%) was at site AQE_KC3 in inner London. In addition, both the second large reduction in roadside concentrations of NO_2 (AQE_CD009) and NO_x were observed at the boundary between central and inner London. Unlike NO_2, which showed a statistically significant reduction at all roadside sites, the NO_x concentrations at 7 of 45 roadside sites showed an insignificant or null response, and all these sites are located outside central London.

Compared with roadside concentrations, average reductions in background concentrations of NO_x generally showed less variation across different subregions; the lockdown caused an average reduction in background NO_x concentrations of 5%-7% in individual subregions, with inner London showing the smallest average reduction. Furthermore, the response ratio for background NO_x concentrations is also much smaller than for roadside sites (roadside: 93%; background: 70%). Particularly, only 63% and 71% of the background sites in outer and inner London, respectively, showed a response in NO_x concentrations. At individual background sites, only 2 background sites in central London met the data quality criteria, both indicating a pollution reduction of ~6%. The highest reduction in background NO_x concentrations (33%) across the city was at site AQE_HI3, and the second large reduction (23%) was at site AQE_SIPS; both sites are in outer London and near LHR. In addition, the highest reduction in background NO_x concentrations in inner London was at site LAQN_WA2; both the highest reductions in background NO_2 and NO_x concentrations in inner London were at a major town centre in southwest London (NO_2: 17%, LAQN_LB6; NO_x: 17%, LAQN_WA2).

Comparing results for NO_x with those of NO_2, the response ratio for NO_x concentrations was similar for roadside sites, while there was a large difference between these pollutants for background sites, with a mixed situation. Specifically, the response ratio for NO_x was much greater than NO_2 among the background sites in inner London (57% for NO_2; 71% for NO_x) and outside the GLA (60% for NO_2; 80% for NO_x); however, the situation was opposite for background sites in outer London (95% for NO_2; 63% for NO_x). Considering the difference in the regional mean effect between NO_2 and NO_x in the same subregion, NO_2 concentrations (roadside or background) generally showed a higher average reduction than NO_x, by up to 3%. Two exceptions were observed, respectively, at the roadside sites in central and inner London, where the average reduction in NO_x was higher, yet the differences were both small (~1%). As for the estimated effects at individual monitoring sites, the NO_2 concentrations were more statistically significantly reduced, yet the NO_x concentrations experienced a higher maximum reduction (c.f. Figure 2 in the manuscript and Figure S13-1 in this section).

The difference in response ratios for background NO_x and NO_2 concentrations likely reflects complex atmospheric chemical reactions involving NO, NO_2, and O_3. The differences in estimated effects for NO_x and NO_2 at the same location were generally site-specific (see details in the Supporting Information §S14); the difference could have been influenced by atmospheric chemistry, change in vehicle fleet (particularly in the composition of passenger vehicles and freight vehicles), changes in driving speed, changes in domestic and commercial heating, and changes in commercial cooking, which affected the vehicle NO_x emissions factors, the fraction of NO_x emitted as NO_2, and the composition of emissions sources[41,58,61–63].

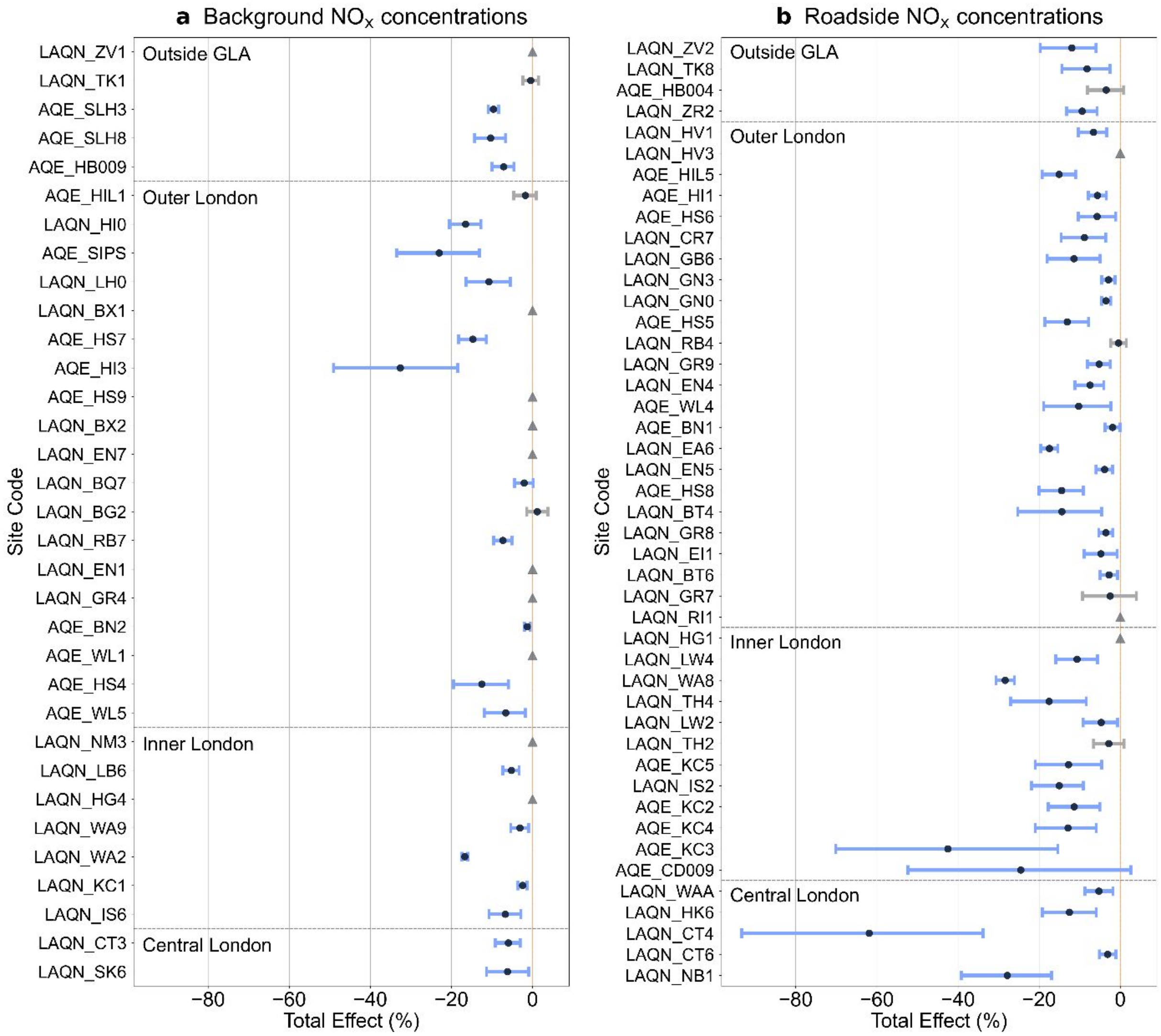

Figure S13-1. Estimated total effects on NO_x concentrations. Central estimates are indicated with black dots. The 95% CIs are illustrated with uncertainty bars (blue: pollution reduction; red: pollution increase). Null responses are denoted by grey triangles. A detected response yet statistically insignificant (at the 10% level) is indicated with grey interval bars. Sites within central London, inner London, outer London, and outside GLA (from bottom to top) are separated by the grey horizontal dashed lines. Sites are sorted by the distance to the centroid of central London. Sites are labelled with data source and site code.

Table S13-1. Summary of the lockdown's effects on NO_x concentrations across London.

		Background NO_x		Roadside NO_x	
		Total Effect (%) [(a, b)]	Adj. R^{2} [(f)]	Total Effect (%) [(a, b)]	Adj. R^{2} [(f)]
London	*Response*	*23 of 33 sites*		*42 of 45 sites*	
	std	7.96	0.066	11.76	0.019
	min	-32.62	0.795	-61.86	0.906
	max	0.00	0.996	0.00	0.999
	mean response [(c, e)]	-8.83*** [-12.85, -5.64]		-11.65*** [-16.09, -8.28]	
	**regional mean** [(d, e)]	-6.05*** [-8.99, -3.57]		-10.90*** [-15.56, -7.70]	

(a) The total effect includes the impact from the current period and the stacked impacts from the lagged periods. Interval estimate is simulated with 10,000 Monte Carlo iterations. Standard errors of coefficients are heteroscedasticity and autocorrelation consistent using 7 lags and without small sample correction.

(b) The standard deviation, minimum value, and maximum value are provided with statistically insignificant estimates (at the 10% level) adjusted to zero.

(c) The mean response is the aggregated effect across all sites where the concentrations responded to the intervention.

(d) The regional mean is the aggregated effect across all sites.

(e) The aggregated effect is computed with 1,000 bootstrap resampling iterations. The 95% CI of aggregated effect (in bracket) is the percentile interval of 1,000 bootstrap resampling iterations. Statistical significance: *** Significant at the 1% level; ** Significant at the 5% level; * Significant at the 10% level.

(f) The adjusted R^2 indicates the performance of the RDD model. The standard deviation, minimum value, and maximum value are provided by summarising the model performance across all RDD models.

Table S13-2. Summary of the lockdown's effects on NO_x concentrations in individual subregions.

		Background NO_x		Roadside NO_x	
		Total Effect (%) (a, b)	Adj. $R^{2\ (f)}$	Total Effect (%) (a, b)	Adj. $R^{2\ (f)}$
Central London	*Response*	*2 of 2 sites*		*5 of 5 sites*	
	std	0.14	0.006	24.23	0.005
	min	-6.15	0.983	-61.86	0.987
	max	-5.95	0.992	-3.12	0.998
	mean response (c, e)	-6.00*** [-9.65, -2.31]		-22.30*** [-46.10, -5.48]	
	**regional mean** (d, e)	-6.00*** [-9.65, -2.31]	-9.65	-22.30*** [-46.10, -5.48]	
Inner London	*Response*	*5 of 7 sites*		*11 of 12 sites*	
	std	5.76	0.081	11.76	0.002
	min	-16.70	0.799	-42.47	0.992
	max	-2.47	0.984	0.00	0.999
	mean response (c, e)	-6.78*** [-11.88, -2.80]		-16.35*** [-25.95, -9.30]	
	**regional mean** (d, e)	-4.93*** [-9.50, -1.34]		-15.06*** [-24.31, -7.68]	
Outer London	*Response*	*12 of 19 sites*		*22 of 24 sites*	
	std	10.02	0.073	5.16	0.022
	min	-32.62	0.795	-17.44	0.906
	max	0.00	0.996	0.00	0.997
	mean response (c, e)	-10.56*** [-17.44, -5.04]		-7.36*** [-10.15, -5.13]	
	**regional mean** (d, e)	-6.63*** [-11.76, -2.85]		-6.74*** [-9.14, -4.42]	
Outside GLA	*Response*	*4 of 5 sites*		*4 of 4 sites*	
	std	4.73	0.026	5.17	0.011
	min	-10.34	0.933	-11.95	0.961
	max	0.00	0.990	0.00	0.987
	mean response (c, e)	-6.84*** [-10.72, -2.57]		-8.27*** [-13.14, -3.88]	
	**regional mean** (d, e)	-5.43** [-9.56, -1.37]		-8.27*** [-13.14, -3.88]	

(a) The total effect includes the impact from the current period and the stacked impacts from the lagged periods. Interval estimate is simulated with 10,000 Monte Carlo iterations. Standard errors of coefficients are HAC
(b) using 7 lags and without small sample correction.
(c) The standard deviation, minimum value, and maximum value are provided with statistically insignificant estimates (at the 10% level) adjusted to zero.
(d) The mean response is the aggregated effect across all sites where the concentrations responded to the intervention.
(e) The regional mean is the aggregated effect across all sites.
(f) The aggregated effect is computed with 1,000 bootstrap resampling iterations. The 95% CI of aggregated effect (in bracket) is the percentile interval of 1,000 bootstrap resampling iterations. Statistical significance: *** Significant at the 1% level; ** Significant at the 5% level; * Significant at the 10% level.
(g) The adjusted R^2 indicates the performance of the RDD model. The standard deviation, minimum value, and maximum value are provided by summarising the model performance across all RDD models.

S14. Effects on O_3 concentrations

The estimated effects of the lockdown on O_3 concentrations at different monitoring sites are illustrated in Figure S14-1. The results are summarised in Table S14-1 for the London-wide effects and further summarised in Table S14-2 for individual subregions. Only 29% of the monitoring sites (background: 2/10; roadside: 3/7) measuring O_3 responded to the lockdown. The effect estimates indicate that the lockdown statistically significantly increased O_3 concentrations at some specific locations, yet all within 1% to 4%. Aggregating the effects at individual monitoring sites, the lockdown had a statistically insignificant effect on O_3 concentrations averaging across London or any subregions in the city. The most significant increase in O_3 concentrations (4%) was observed at the only background site within central London. Compared with NO_2 and NO_x, the effects of lockdown on O_3 concentrations were less significant.

Effect estimates where NO_2, NO_x, and O_3 are simultaneously measured are shown in Table S14-3. Four of the nine background sites showed a concurrent decrease in NO_2 and NO_x, yet no responses in O_3 concentrations were detected. Simultaneous effects were more common on roadside concentrations, and we observe them at all seven roadside monitoring sites. In particular, the lockdown statistically significantly changed the concentrations of all these pollutants at site LAQN_BT4, where NO_2 and NO_x concentrations were decreased by 2% and 14%, respectively, while O_3 concentrations were increased by 1%. The negative correlation in the direction of changes in O_3 (increase) and NO_2 and NO_x (decrease) is consistent with theory and observations[58,64]. In addition, among the 15 sites where the lockdown decreased both NO_2 and NO_x, 4 sites (background: 1 site; roadside: 3 sites) showed a higher reduction in NO_2 concentrations, while 7 sites (background: 3 sites; roadside: 4 sites) showed a higher decrease in NO_x concentrations. The results highlight the spatial heterogeneity of lockdown impacts and the necessity of a site-specific effect attribution, such as by using SHAP values (a local explanation) as in our paper.

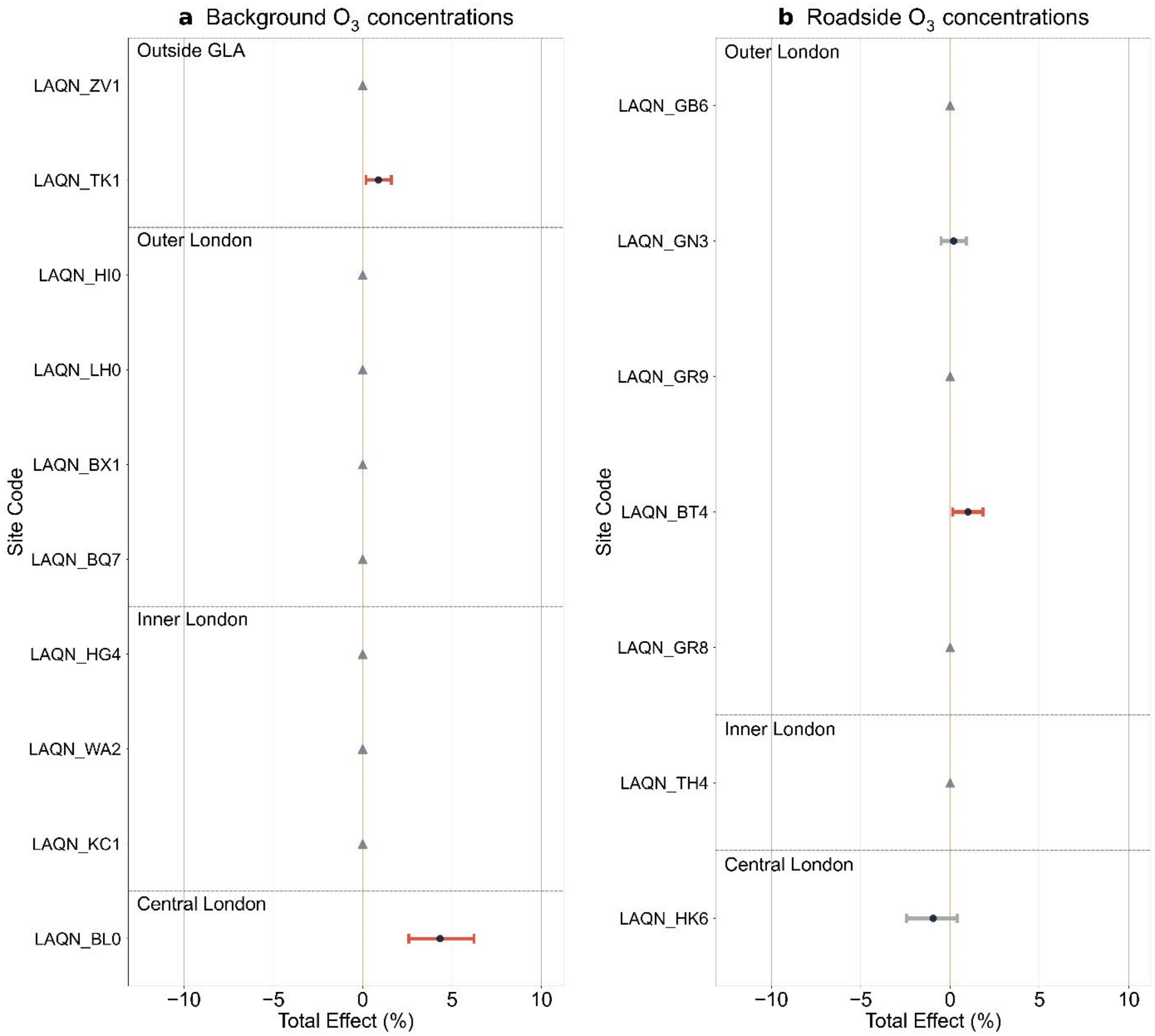

Figure S14-1. Estimated total effects on O_3 concentrations. Central estimates are indicated with black dots. The 95% CIs are illustrated with uncertainty bars (blue: pollution reduction; red: pollution increase). Null responses are denoted by grey triangles. A detected response yet statistically insignificant (at the 10% level) is indicated with grey interval bars. Sites within central London, inner London, outer London, and outside GLA (from bottom to top) are separated by the grey horizontal dashed lines. Sites are sorted by the distance to the centroid of central London. Sites are labelled with data source and site code.

Table S14-1. Summary of the lockdown's effects on O_3 concentrations across London.

		Background O_3		Roadside O_3	
		Total Effect (%) [(a, b)]	Adj. R^{2} [(f)]	Total Effect (%) [(a, b)]	Adj. R^{2} [(f)]
London	*Response*	*2 of 10 sites*		*3 of 7 sites*	
	std	2.44	0.037	0.58	0.011
	min	0.88	0.933	0.00	0.964
	max	4.33	0.984	1.00	0.984
	mean response [(c, e)]	2.60*** [0.52, 5.43]		0.05 [-1.25, 1.12]	
	**regional mean** [(d, e)]	0.52 [0.00, 1.49]		0.04 [-0.57, 0.60]	

(a) The total effect includes the impact from the current period and the stacked impacts from the lagged periods. Interval estimate is simulated with 10,000 Monte Carlo iterations. Standard errors of coefficients are heteroscedasticity and autocorrelation consistent using 7 lags and without small sample correction.
(b) The standard deviation, minimum value, and maximum value are provided with statistically insignificant estimates (at the 10% level) adjusted to zero.
(c) The mean response is the aggregated effect across all sites where the concentrations responded to the intervention.
(d) The regional mean is the aggregated effect across all sites.
(e) The aggregated effect is computed with 1,000 bootstrap resampling iterations. The 95% CI of aggregated effect (in bracket) is the percentile interval of 1,000 bootstrap resampling iterations. Statistical significance: *** Significant at the 1% level; ** Significant at the 5% level; * Significant at the 10% level.
(f) The adjusted R^2 indicates the performance of the RDD model. The standard deviation, minimum value, and maximum value are provided by summarising the model performance across all RDD models.

Table S14-2. Summary of the lockdown's effects on O_3 concentrations in individual subregions.

		Background O_3		Roadside O_3	
		Total Effect (%) [a, b]	Adj. R^2 [f]	Total Effect (%) [a, b]	Adj. R^2 [f]
Central London	*Response*	*1 of 1 site*		*1 of 1 site*	
	std	-	-	-	-
	min	4.33	0.984	0.00	0.964
	max	4.33	0.984	0.00	0.964
	mean response [c, e]	4.33*** [2.59, 6.23] [g]		-0.95 [-2.43, 0.41] [g]	
	**regional mean** [d, e]	4.33*** [2.59, 6.23] [g]		-0.95 [-2.43, 0.41] [g]	
Inner London	*Response*	*0 of 3 sites*		*0 of 1 site*	
	std	-	-	-	-
	min	-	-	-	-
	max	-	-	-	-
	mean response [c, e]	-		-	
	**regional mean** [d, e]	0.00		0.00	
Outer London	*Response*	*0 of 4 sites*		*2 of 5 sites*	
	std	-	-	0.71	0.002
	min	-	-	0.00	0.982
	max	-	-	1.00	0.984
	mean response [c, e]	-		0.60 [-0.29, 1.48]	
	**regional mean** [d, e]	0.00		0.23 [-0.10, 0.75]	
Outside GLA	*Response*	*1 of 2 sites*		*0 site* [h]	
	std	-	-	-	-
	min	0.88	0.933	-	-
	max	0.88	0.933	-	-
	mean response [c, e]	0.88** [0.19, 1.61] [g]		-	
	**regional mean** [d, e]	0.44 [0.00, 1.38]		-	

(a) The total effect includes the impact from the current period and the stacked impacts from the lagged periods. Interval estimate is simulated with 10,000 Monte Carlo iterations. Standard errors of coefficients are HAC using 7 lags and without small sample correction.

(b) The standard deviation, minimum value, and maximum value are provided with statistically insignificant estimates (at the 10% level) adjusted to zero.

(c) The mean response is the aggregated effect across all sites where the concentrations responded to the intervention.

(d) The regional mean is the aggregated effect across all sites.

(e) The aggregated effect is computed with 1,000 bootstrap resampling iterations. The 95% CI of aggregated effect (in bracket) is the percentile interval of 1,000 bootstrap resampling iterations. Statistical significance: *** Significant at the 1% level; ** Significant at the 5% level; * Significant at the 10% level.

(f) The adjusted R^2 indicates the performance of the RDD model. The standard deviation, minimum value, and maximum value are provided by summarising the model performance across all RDD models.

(g) Only one site is in the group. In this case, the central estimate and 95% CI of the aggregated effect are represented by the corresponding metric of the effect estimate at this particular monitoring site.
(h) No sites in the region met the data quality criteria.

Table S14-3. Total effect comparison among O_3, NO_2, and NO_x concentrations at sites where pollutants are simultaneously measured.

Location	Site	Sub-region	O_3 concentrations		NO_2 concentrations		NO_x concentrations	
			Total effect [a, b] (%)	Sig.[c]	Total effect [a, b] (%)	Sig.[c]	Total effect [a, b] (%)	Sig.[c]
Background	LAQN_KC1	Inner	-		-		-2.47	***
	LAQN_WA2	Inner	-		-12.25	***	-16.70	***
	LAQN_HG4	Inner	-		-1.60	**	-	
	LAQN_BQ7	Outer	-		-5.35	***	-2.06	*
	LAQN_BX1	Outer	-		-		-	
	LAQN_LH0	Outer	-		-6.00	***	-10.77	***
	LAQN_HI0	Outer	-		-14.45	***	-16.53	***
	LAQN_TK1	Outside	0.88	**	-		-0.46	
	LAQN_ZV1	Outside	-		-		-	
Roadside	LAQN_HK6	Central	-0.95		-13.72	***	-12.54	***
	LAQN_TH4	Inner	-		-10.70	***	-17.53	***
	LAQN_GR8	Outer	-		-3.73	***	-3.55	***
	LAQN_BT4	Outer	1.00	**	-2.46	**	-14.39	***
	LAQN_GR9	Outer	-		-2.64	***	-5.21	***
	LAQN_GN3	Outer	0.20		-10.87	***	-2.89	***
	LAQN_GB6	Outer	-		-5.97	***	-11.42	***

(a) The total effect includes the impact from the current period and the stacked impacts from the lagged periods. Interval estimate is simulated with 10,000 Monte Carlo iterations. Standard errors of coefficients are HAC using 7 lags and without small sample correction
(b) The site that has no detected change points within the margin period is indicated with '-'.
(c) Statistical significance: *** Significant at the 1% level; ** Significant at the 5% level; * Significant at the 10% level.

S15. Effects on PM concentrations

The estimated effects of the lockdown on PM concentrations at different monitoring sites are illustrated in Figure S15-1 and Figure S15-2 for PM_{10} and $PM_{2.5}$, respectively; the results are summarised in Table S15-1 for the London-wide effects and in Table S15-2 for different subregions. Only 29% of the monitoring sites (background: 6/26; roadside: 12/36) showed a response to the lockdown in PM_{10} concentrations, and there was no response for $PM_{2.5}$. Effect estimates show that the lockdown statistically significantly changed PM concentrations at some specific locations; however, the city-wide mean effects for PM_{10} and $PM_{2.5}$ were all insignificant. As the regional contribution to PM is substantial and more than half of the $PM_{2.5}$ concentrations in London are from regional sources outside London[59], the results for PM imply that a significant decrease in traffic volume and vehicle exhaust emissions may have subtle effects on PM concentrations in London in general.

Specifically, for PM_{10}, the lockdown changed the daily average PM_{10} concentrations (roadside or background) by -0% [-1%, +0%] on average in London, ranging from -5% to +0% at background sites and from -3% to 0% at roadside sites. The roadside sites generally had a higher response ratio for PM_{10} than the background sites, both across London and in particular subregions (except for the area outside GLA). The response ratio of roadside sites in different subregions generally decreased with the distance to the city centre; 75% for central London, 50% for inner London, 27% for outer London, and 0% for the region outside the GLA. However, in most subregions, the lockdown generally had an insignificant average effect on PM_{10} concentrations (roadside or background); only the roadside PM_{10} concentrations in central London were statistically significantly reduced on average, yet with a small mean effect (regional mean: -1% [-3%, -0%]).

Among the sites that showed a response, both the highest reductions in roadside and background concentrations of PM_{10} were in central London, respectively at LAQN_WAA (3%) and LAQN_WM0 (5%). No statistically significant effects were found on PM_{10} concentrations in inner London. Six sites in outer London (2 background sites + 4 roadside sites) showed a statistically significant decrease in PM_{10} concentrations, one of which was at a background site near LHR (site LAQN_LH0; 2%). It is noted that the lockdown statistically significantly increased PM_{10} concentrations at a background site, yet the magnitude was small (< 1%).

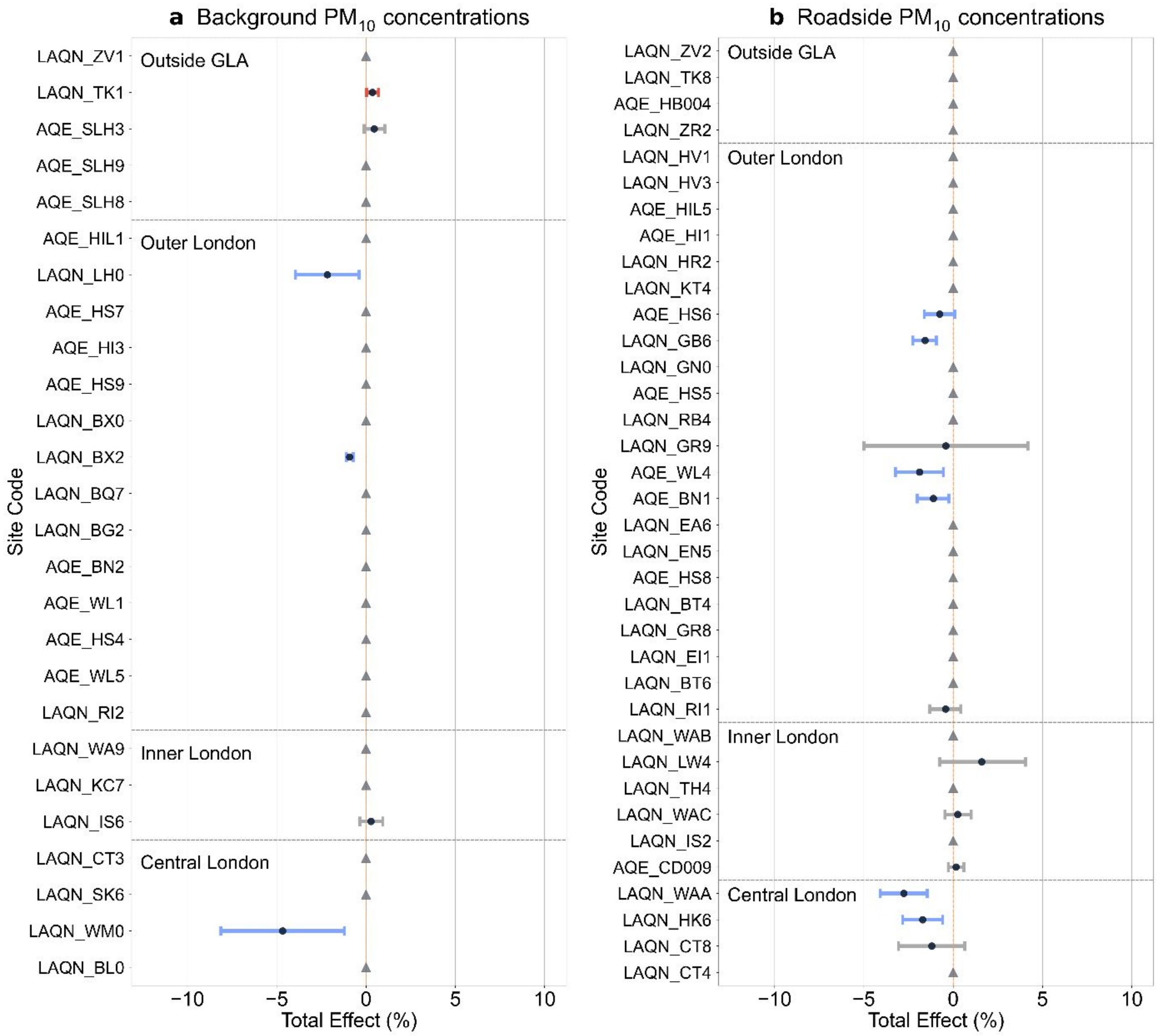

Figure S15-1. Estimated total effects on PM_{10} concentrations. Central estimates are indicated with black dots. The 95% CIs are illustrated with uncertainty bars (blue: pollution reduction; red: pollution increase). Null responses are denoted by grey triangles. A detected response yet statistically insignificant (at the 10% level) is indicated with grey interval bars. Sites within central London, inner London, outer London, and outside GLA (from bottom to top) are separated by the grey horizontal dashed lines. Sites are sorted by the distance to the centroid of central London. Sites are labelled with data source and site code.

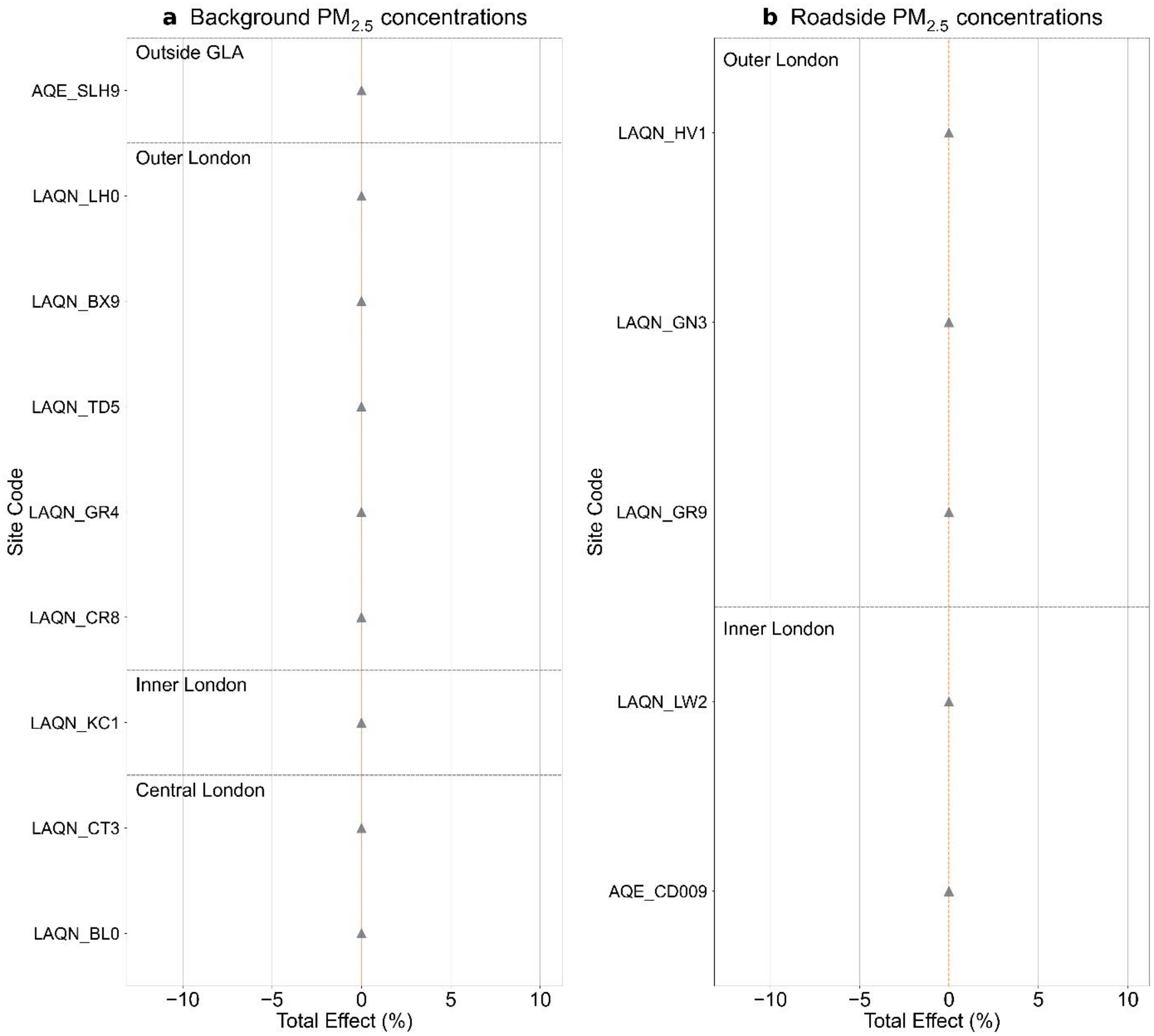

Figure S15-2. Estimated total effects on $PM_{2.5}$ concentrations. Central estimates are indicated with black dots. The 95% CIs are illustrated with uncertainty bars (blue: pollution reduction; red: pollution increase). Null responses are denoted by grey triangles. A detected response yet statistically insignificant (at the 10% level) is indicated with grey interval bars. Sites within central London, inner London, outer London, and outside GLA (from bottom to top) are separated by the grey horizontal dashed lines. Sites are sorted by the distance to the centroid of central London. Sites are labelled with data source and site code.

Table S15-1. Summary of the lockdown's effects on PM concentrations across London.

		Background PM_{10}		Roadside PM_{10}		Background $PM_{2.5}$		Roadside $PM_{2.5}$	
		Total Effect (%) [a, b]	Adj. R^2 [f]	Total Effect (%) [a, b]	Adj. R^2 [f]	Total Effect (%) [a, b]	Adj. R^2 [f]	Total Effect (%) [a, b]	Adj. R^2 [f]
London	*Response*	*6 of 26 sites*		*12 of 36 sites*		*0 of 9 sites*		*0 of 5 sites*	
	std	1.91	0.122	0.97	0.052	-	-	-	-
	min	-4.66	0.621	-2.76	0.816	-	-	-	-
	max	0.36	0.975	0.00	0.992	-	-	-	-
	mean response [c, e]	-1.12 [-3.28, 0.22]		-0.80* [-1.68, 0.14]		-		-	
	**regional mean** [d, e]	-0.26 [-0.80, 0.05]		-0.27 [-0.66, 0.09]		0.00		0.00	

(a) The total effect includes the impact from the current period and the stacked impacts from the lagged periods. Interval estimate is simulated with 10,000 Monte Carlo iterations. Standard errors of coefficients are heteroscedasticity and autocorrelation consistent using 7 lags and without small sample correction.

(b) The standard deviation, minimum value, and maximum value are provided with statistically insignificant estimates (at the 10% level) adjusted to zero.

(c) The mean response is the aggregated effect across all sites where the concentrations responded to the intervention.

(d) The regional mean is the aggregated effect across all sites.

(e) The aggregated effect is computed with 1,000 bootstrap resampling iterations. The 95% CI of aggregated effect (in bracket) is the percentile interval of 1,000 bootstrap resampling iterations. Statistical significance: *** Significant at the 1% level; ** Significant at the 5% level; * Significant at the 10% level.

(f) The adjusted R^2 indicates the performance of the RDD model. The standard deviation, minimum value, and maximum value are provided by summarising the model performance across all RDD models.

Table S15-2. Summary of the lockdown's effects on PM concentrations in individual subregions.

		Background PM_{10}		Roadside PM_{10}		Background $PM_{2.5}$		Roadside $PM_{2.5}$	
		Total Effect (%) [a, b]	Adj. R^2 [f]	Total Effect (%) [a, b]	Adj. R^2 [f]	Total Effect (%) [a, b]	Adj. R^2 [f]	Total Effect (%) [a, b]	Adj. R^2 [f]
Central London	*Response*	*1 of 4 sites*		*3 of 4 sites*		*0 of 2 sites*		*0 site* [h]	
	std	-	-	1.39	0.021	-	-	-	-
	min	-4.66	0.975	-2.76	0.953	-	-	-	-
	max	-4.66	0.975	0.00	0.992	-	-	-	-
	mean response [c, e]	-4.66*** [-8.12, -1.21] [g]		-1.92** [-3.13, -0.53]		-		-	
	**regional mean** [d, e]	-1.15 [-4.04, 0.00]		-1.41** [-2.75, -0.24]		0.00		-	
Inner London	*Response*	*1 of 3 sites*		*3 of 6 sites*		*0 of 1 site*		*0 of 2 sites*	
	std	-	-	0.00	0.026	-	-	-	-
	min	0.00	0.904	0.00	0.937	-	-	-	-
	max	0.00	0.904	0.00	0.988	-	-	-	-
	mean response [c, e]	0.28 [-0.35, 0.93] [g]		0.71 [-0.22, 2.61]		-		-	
	**regional mean** [d, e]	0.10 [-0.11, 0.56]		0.32 [-0.14, 1.21]		0.00		0.00	
Outer London	*Response*	*2 of 14 sites*		*6 of 22 sites*		*0 of 5 sites*		*0 of 3 sites*	
	std	0.88	0.169	0.79	0.050	-	-	-	-
	min	-2.16	0.621	-1.88	0.816	-	-	-	-
	max	-0.91	0.861	0.00	0.965	-	-	-	-
	mean response [c, e]	-1.56*** [-3.32, -0.69]		-1.01 [-2.23, 0.48]		-		-	
	**regional mean** [d, e]	-0.22 [-0.67, 0.00]		-0.28 [-0.71, 0.11]		0.00		0.00	
Outside GLA	*Response*	*2 of 5 sites*		*0 of 4 sites*		*0 of 1 site*		*0 site* [h]	
	std	0.26	0.038	-	-	-	-	-	-
	min	0.00	0.848	-	-	-	-	-	-
	max	0.36	0.903	-	-	-	-	-	-
	mean response [c, e]	0.41** [0.02, 0.86]		-		-		-	
	**regional mean** [d, e]	0.16 [0.00, 0.46]		0.00		0.00		-	

(a) The total effect includes the impact from the current period and the stacked impacts from the lagged periods. Interval estimate is simulated with 10,000 Monte Carlo iterations. Standard errors of coefficients are HAC using 7 lags and without small sample correction.
(b) The standard deviation, minimum value, and maximum value are provided with statistically insignificant estimates (at the 10% level) adjusted to zero.
(c) The mean response is the aggregated effect across all sites where the concentrations responded to the intervention.
(d) The regional mean is the aggregated effect across all sites.
(e) The aggregated effect is computed with 1,000 bootstrap resampling iterations. The 95% CI of aggregated effect (in bracket) is the percentile interval of 1,000 bootstrap resampling iterations. Statistical significance: *** Significant at the 1% level; ** Significant at the 5% level; * Significant at the 10% level.
(f) The adjusted R^2 indicates the performance of the RDD model. The standard deviation, minimum value, and maximum value are provided by summarising the model performance across all RDD models.

(g) Only one site is in the group. In this case, the central estimate and 95% CI of the aggregated effect are represented by the corresponding metric of the effect estimate at this particular monitoring site.

(h) No sites in the region met the data quality criteria.

S16. Example of site-specific attribution

This section provides an example of site-specific attribution with local SHAP values, where the contribution of individual features to predicting pollution reduction is specific to a particular spatial location.

We select the case of Lambeth 030 (MSOA code: E02000647) to provide the example, and the selected MSOA is estimated to have the highest relative pollution reduction across London. The SHAP values specific to this location are illustrated in Figure S16-1 and the SHAP value of the top features shown in this figure is particularly given in Table S16-1. As indicated in Figure S16-1, the mean relative pollution reduction predicted with the fitted GBDT model (c.f. SI §S10) across different MSOAs, which is called *base value*, is 4.23%. The predicted relative pollution reduction in Lambeth 030 is 11.83%, which is higher than the London-wide average. The top features that contributed to bringing the prediction higher than the base value include the proportion of HGVs (labelled as [TraV]_hgv%), the distance to LHR (labelled as [Dist]_LHR), and the distance to the Central Activities Zone (labelled as [Dist]_Central_Activities) (Figure S16-1). Combining with a comparison in feature values between Lambeth 030 and other MSOAs, the local SHAP values in Table S16-1 indicate that a small proportion of HGVs (less than 25% percentile), a moderate distance to LHR (slightly smaller than the median), and a moderate distance to the Central Activities Zone (slightly smaller than the median) contributed to increasing the pollution reduction at this MSOA by 4.28 percentage points (pp), 1.13 pp, and 0.70 pp, respectively.

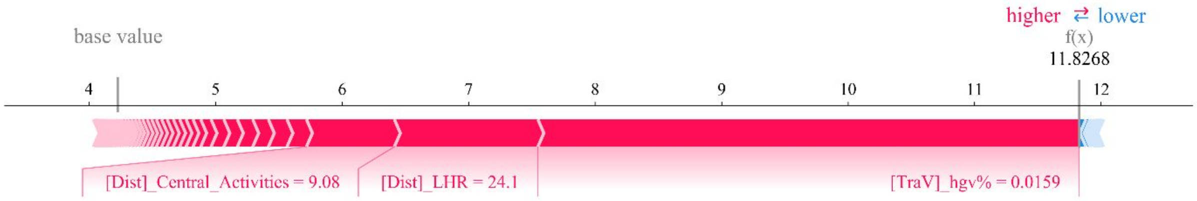

Figure S16-1. Estimated SHAP values for the instance of Lambeth 030 (E02000647). The top features are labelled with the corresponding feature values. The base value is the average prediction of the relative lockdown impact (pp) from the GBDT model across all instances. The prediction specific to this instance is marked above the axis. Higher absolute SHAP values are illustrated with larger arrows. Features are coloured according to their influence on the value of the prediction (red: increasing pollution reduction; blue: decreasing pollution reduction).

Table S16-1. Estimated SHAP value of the top features for the instance of Lambeth 030.

Feature label	Feature name	SHAP value [(a)] (pp)
[TraV]_hgv%	Proportion of HGVs in road motor vehicle traffic	4.28
[Dist]_LHR	Distance to London Heathrow Airport	1.13
[Dist]_Central_Activities	Distance to Central Activities Zone	0.70

(a) SHAP value is in the same unit as the model output.

References

(1) Transport for London. *Travel in London Report 13*; 2020. http://content.tfl.gov.uk/travel-in-london-report-13.pdf (accessed 2021-01-25).

(2) Greater London Authority. *The London Plan*; 2021.

(3) Office for National Statistics. *London Boroughs (December 2018) Map in London*. https://geoportal.statistics.gov.uk/documents/ons::london-boroughs-december-2018-map-in-london/explore (accessed 2021-10-02).

(4) Imperial College London. *London Air Quality Network*. https://www.londonair.org.uk/london/asp/datadownload.asp (accessed 2020-09-29).

(5) Carslaw, D. C.; Ropkins, K. Openair — An R Package for Air Quality Data Analysis. *Environmental Modelling & Software* **2012**, *27–28*, 52–61. https://doi.org/10.1016/J.ENVSOFT.2011.09.008.

(6) NOAA. *Integrated Surface Database*. NOAA National Centers for Environmental Information. https://www.ncdc.noaa.gov/isd (accessed 2020-09-29).

(7) NOAA. *Radiosonde Database*. NOAA Earth System Research Laboratory. https://ruc.noaa.gov/raobs/ (accessed 2020-09-29).

(8) EPA. Meteorological Processors and Accessory Programs. JLE 2019.

(9) Greater London Authority. *Town Centre Boundaries*. https://data.london.gov.uk/dataset/town_centre_boundaries (accessed 2021-04-26).

(10) Office for National Statistics. *Population estimates - small area based by single year of age - England and Wales*. https://www.nomisweb.co.uk/datasets/pestsyoaoa (accessed 2021-04-26).

(11) Office for National Statistics. *Business Register and Employment Survey*. https://www.nomisweb.co.uk/datasets/newbres6pub (accessed 2021-04-30).

(12) Office for National Statistics. *annual survey of hours and earnings - resident analysis*. https://www.nomisweb.co.uk/datasets/asher (accessed 2021-04-26).

(13) Office for National Statistics. *annual survey of hours and earnings - workplace analysis*. https://www.nomisweb.co.uk/datasets/ashe (accessed 2021-04-26).

(14) Office for National Statistics. *UK Business Counts - local units by industry and employment size band*. https://www.nomisweb.co.uk/datasets/idbrlu (accessed 2021-05-01).

(15) Office for National Statistics. *UK Business Counts - enterprises by industry and turnover size*. https://www.nomisweb.co.uk/query/construct/summary.asp?mode=construct&version=0&dataset=199 (accessed 2021-05-01).

(16) Transport for London. Public Transport Access Level. **2021**.

(17) Department for Transport. *Road traffic statistics*. https://roadtraffic.dft.gov.uk/downloads (accessed 2021-04-26).

(18) Department for Transport. *Traffic by local authority*. https://www.gov.uk/government/statistical-data-sets/road-traffic-statistics-tra#traffic-by-local-authority-tra89 (accessed 2021-04-27).

(19) Met Office; Hollis, D.; McCarthy, M.; Kendon, M.; Legg, T.; SImpson, I. HadUK-Grid Gridded and Regional Average Climate Observations for the UK. *Centre for Environmental Data Analysis* **2018**.

(20) Danielson, J. J.; Gesh, D. B. *Global Multi-Resolution Terrain Elevation Data 2010 (GMTED2010): U.S. Geological Survey Open-File Report 2011–1073*; 2011.

(21) LAEI. *London Atmospheric Emissions Inventory (LAEI) 2016*. https://data.london.gov.uk/dataset/london-atmospheric-emissions-inventory--laei--2016 (accessed 2021-07-07).

(22) Department for Environment Food & Rural Affairs. *Modelled background pollution data*. https://uk-air.defra.gov.uk/data/pcm-data (accessed 2021-07-05).

(23) Ma, L.; Graham, D. J.; Stettler, M. E. J. Air Quality Impacts of New Public Transport Provision: A Causal Analysis of the Jubilee Line Extension in London. *Atmos Environ* **2021**, *245*. https://doi.org/10.1016/j.atmosenv.2020.118025.

(24) Ma, L.; Graham, D. J.; Stettler, M. E. J. Has the Ultra Low Emission Zone in London Improved Air Quality? *Environmental Research Letters* **2021**, *16* (12), 124001. https://doi.org/10.1088/1748-9326/ac30c1.

(25) Hausman, C.; Rapson, D. S. Regression Discontinuity in Time: Considerations for Empirical Applications. *Annu Rev Resour Economics* **2018**, *10*, 533–552. https://doi.org/10.1146/annurev-resource-121517-033306.

(26) Ke, G.; Meng, Q.; Finley, T.; Wang, T.; Chen, W.; Ma, W.; Ye, Q.; Liu, T. Y. LightGBM: A Highly Efficient Gradient Boosting Decision Tree. In *Advances in Neural Information Processing Systems*; 2017; Vol. 2017-Decem, pp 3147–3155.

(27) Pedregosa, F.; Grisel, O.; Weiss, R.; Passos, A.; Brucher, M.; Varoquax, G.; Gramfort, A.; Michel, V.; Thirion, B.; Grisel, O.; Blondel, M.; Prettenhofer, P.; Weiss, R.; Dubourg, V.; Brucher, M. Scikit-Learn: Machine Learning in Python. *Journal of Machine Learning Research* **2011**, *12* (85), 2825–2830.

(28) Friedman, J. H. Greedy Function Approximation: A Gradient Boosting Machine. *Ann Stat* **2001**, *29* (5), 1189–1232. https://doi.org/10.1214/aos/1013203451.

(29) Dixon, P. M. Bootstrap Resampling. In *Encyclopedia of Environmetrics*; John Wiley & Sons, Ltd, 2001. https://doi.org/10.1002/9780470057339.vab028.

(30) Grange, S. K.; Carslaw, D. C. Using Meteorological Normalisation to Detect Interventions in Air Quality Time Series. *Science of the Total Environment* **2019**, *653*, 578–588. https://doi.org/10.1016/j.scitotenv.2018.10.344.

(31) de Foy, B.; Heo, J.; Kang, J. Y.; Kim, H.; Schauer, J. J. Source Attribution of Air Pollution Using a Generalized Additive Model and Particle Trajectory Clusters. *Science of the Total Environment* **2021**, *780*, 146458. https://doi.org/10.1016/j.scitotenv.2021.146458.

(32) Peng, H.; Lima, A. R.; Teakles, A.; Jin, J.; Cannon, A. J.; Hsieh, W. W. Evaluating Hourly Air Quality Forecasting in Canada with Nonlinear Updatable Machine Learning Methods. *Air Qual Atmos Health* **2017**, *10* (2), 195–211. https://doi.org/10.1007/s11869-016-0414-3.

(33) Wen, Y.; Wang, H.; Larson, T.; Kelp, M.; Zhang, S.; Wu, Y.; Marshall, J. D. On-Highway Vehicle Emission Factors, and Spatial Patterns, Based on Mobile Monitoring and Absolute Principal Component Score. *Science of the Total Environment* **2019**, *676*, 242–251. https://doi.org/10.1016/j.scitotenv.2019.04.185.

(34) Hubert, M.; Vandervieren, E. An Adjusted Boxplot for Skewed Distributions. *Comput Stat Data Anal* **2008**, *52* (12), 5186–5201. https://doi.org/10.1016/J.CSDA.2007.11.008.

(35) Truong, C.; Oudre, L.; Vayatis, N. Selective Review of Offline Change Point Detection Methods. *Signal Processing*. Elsevier B.V. February 1, 2020, p 107299. https://doi.org/10.1016/j.sigpro.2019.107299.

(36) Bai, J.; Perron, P. Computation and Analysis of Multiple Structural Change Models. *Journal of Applied Econometrics* **2003**, *18* (1), 1–22. https://doi.org/10.1002/jae.659.

(37) Zhao, Q.; Xu, M.; Fränti, P. Knee Point Detection on Bayesian Information Criterion. In *Proceedings - International Conference on Tools with Artificial Intelligence, ICTAI*; 2008; Vol. 2, pp 431–438. https://doi.org/10.1109/ICTAI.2008.154.

(38) Imbens, G. W.; Wooldridge, J. M. Recent Developments in the Econometrics of Program Evaluation. *J Econ Lit* **2009**, *47* (1), 5–86. https://doi.org/10.1257/jel.47.1.5.

(39) Lee, D. S.; Lemieux, T. Regression Discontinuity Designs in Economics. *J Econ Lit* **2010**, *48* (2), 281–355. https://doi.org/10.1257/jel.48.2.281.

(40) Henderson, J. V. Effects of Air Quality Regulation. *American Economic Review* **1996**, *86* (4), 789–813. https://doi.org/10.2307/2118305.

(41) Air Quality Expert Group. *Estimation of Changes in Air Pollution Emissions, Concentrations and Exposure during the COVID-19 Outbreak in the UK*; 2020.

(42) Horálek, J.; Schreiberová, M.; Schneider, P.; Kurfürst, P.; Schovánková, J.; Ďoubalová, J. *European Air Quality Maps for 2016*; 2019.

(43) Horálek, J.; Denby, B.; de Smet, P. A. M.; de Leeuw, F. A. A. M.; Kurfürst, P.; Swart, R.; van Noije, T. *Spatial Mapping of Air Quality for European Scale Assessment ETC/ACC Technical Paper 2006/6*; 2007.

(44) Cressie, N. Spatial Prediction and Ordinary Kriging. *Mathematical Geology 1988 20:4* **1988**, *20* (4), 405–421. https://doi.org/10.1007/BF00892986.

(45) Wackernagel, H. Ordinary Kriging. In *Multivariate Geostatistics*; Springer, Berlin, Heidelberg, 2003; pp 79–88. https://doi.org/10.1007/978-3-662-05294-5_11.

(46) Oliver, M. A.; Webster, R. A Tutorial Guide to Geostatistics: Computing and Modelling Variograms and Kriging. *Catena (Amst)* **2014**, *113*, 56–69. https://doi.org/10.1016/J.CATENA.2013.09.006.

(47) Mälicke, M.; Möller, E.; Schneider, H. D.; Müller, S. Mmaelicke/Scikit-Gstat: A Scipy Flavoured Geostatistical Variogram Analysis Toolbox, 2021. https://doi.org/10.5281/ZENODO.4835779.

(48) Lundberg, S. M.; Erion, G. G.; Lee, S.-I. Consistent Individualized Feature Attribution for Tree Ensembles. **2018**.

(49) Ribeiro, M. T.; Singh, S.; Guestrin, C. "Why Should i Trust You?" Explaining the Predictions of Any Classifier. In *Proceedings of the ACM SIGKDD International Conference on Knowledge Discovery and Data Mining*; ACM: New York, NY, USA, 2016; Vol. 13-17-Augu, pp 1135–1144. https://doi.org/10.1145/2939672.2939778.

(50) Shrikumar, A.; Greenside, P.; Kundaje, A. Learning Important Features through Propagating Activation Differences. In *34th International Conference on Machine Learning, ICML 2017*; International Machine Learning Society (IMLS), 2017; Vol. 7, pp 4844–4866.

(51) Štrumbelj, E.; Kononenko, I. Explaining Prediction Models and Individual Predictions with Feature Contributions. *Knowl Inf Syst* **2014**, *41* (3), 647–665. https://doi.org/10.1007/s10115-013-0679-x.

(52) Bach, S.; Binder, A.; Montavon, G.; Klauschen, F.; Müller, K.-R.; Samek, W. On Pixel-Wise Explanations for Non-Linear Classifier Decisions by Layer-Wise Relevance Propagation. *PLoS One* **2015**, *10* (7). https://doi.org/10.1371/JOURNAL.PONE.0130140.

(53) Lundberg, S. M.; Lee, S. I. A Unified Approach to Interpreting Model Predictions. In *Advances in Neural Information Processing Systems*; 2017; Vol. 2017-Decem, pp 4766–4775.

(54) Lundberg, S. M.; Erion, G.; Chen, H.; DeGrave, A.; Prutkin, J. M.; Nair, B.; Katz, R.; Himmelfarb, J.; Bansal, N.; Lee, S.-I. From Local Explanations to Global Understanding with Explainable AI for Trees. *Nat Mach Intell* **2020**, *2* (1), 56–67. https://doi.org/10.1038/s42256-019-0138-9.

(55) Shapley, L. S. A Value for N-Person Games. In *Contributions to the Theory of Games*; Princeton University Press, 1953; pp 307–317. https://doi.org/10.1515/9781400881970-018/HTML.

(56) Christoph, M. *Interpretable Machine Learning*; 2019. https://doi.org/10.1201/9780367816377-16.

(57) Janzing, D.; Minorics, L.; Blöbaum, P. Feature Relevance Quantification in Explainable AI: A Causal Problem. **2019**.

(58) Clapp, L. J.; Jenkin, M. E. Analysis of the Relationship between Ambient Levels of O3, NO2 and NO as a Function of NOx in the UK. *Atmos Environ* **2001**, *35* (36), 6391–6405. https://doi.org/10.1016/S1352-2310(01)00378-8.

(59) Greater London Authority. *Air Quality In London 2016-2020*; London, 2020. https://www.london.gov.uk/sites/default/files/air_quality_in_london_2016-2020_october2020final.pdf (accessed 2020-10-12).

(60) Air Quality Expert Group. *Ozone in the UK-Recent Trends and Future Projections*; 2021. https://uk-air.defra.gov.uk/assets/documents/reports/cat09/2112200932_Ozone_in_the_UK_Recent_Trends_and_Future_Projections.pdf (accessed 2022-04-29).

(61) Carslaw, D. C. Evidence of an Increasing NO2/NOX Emissions Ratio from Road Traffic Emissions. *Atmos Environ* **2005**, *39* (26), 4793–4802. https://doi.org/10.1016/j.atmosenv.2005.06.023.

(62) Carslaw, D. C.; Farren, N. J.; Vaughan, A. R.; Drysdale, W. S.; Young, S.; Lee, J. D. The Diminishing Importance of Nitrogen Dioxide Emissions from Road Vehicle Exhaust. *Atmos Environ X* **2019**, *1*, 100002. https://doi.org/10.1016/j.aeaoa.2018.100002.

(63) National Atmospheric Emissions Inventory. *Emission Factors*. https://naei.beis.gov.uk/data/emission-factors (accessed 2021-09-07).

(64) Williams, M. L.; Atkinson, R. W.; Anderson, H. R.; Kelly, F. J. Associations between Daily Mortality in London and Combined Oxidant Capacity, Ozone and Nitrogen Dioxide. *Air Qual Atmos Health* **2014**, *7* (4), 407–414. https://doi.org/10.1007/s11869-014-0249-8.

(65) Linsley, R. K.; Kohler, M. A.; Paulhus, J. L. H. *Hydrology for Engineers*, 3rd ed.; McGraw Hill Book Co, 1982.

(66) Wooldridge, J. M. *Introductory Econometrics: A Modern Approach, Fifth Edition*; South-Western Cengange Learning, 2013.
